# Targeting the E Prostanoid Receptor EP4 Mitigates Cardiac Fibrosis Induced by β‐Adrenergic Activation

**DOI:** 10.1002/advs.202413324

**Published:** 2025-02-07

**Authors:** Hu Xu, Xiuhui Mao, Yali Wang, Chunhua Zhu, Bo Liang, Yihang Zhao, Mengfei Zhou, Lan Ye, Mengting Hong, Huishu Shao, Yashuo Wang, Haonan Li, Yinghui Qi, Yongliang Yang, Lihong Chen, Youfei Guan, Xiaoyan Zhang

**Affiliations:** ^1^ Wuhu Hospital East China Normal University Shanghai 200241 China; ^2^ Health Science Center East China Normal University Shanghai 200241 China; ^3^ Advanced Institute for Medical Sciences Dalian Medical University Dalian 116044 China; ^4^ MOE Key Laboratory of Bio‐Intelligent Manufacturing School of Bioengineering Dalian University of Technology Dalian 116024 China; ^5^ Department of Nephrology Pudong New District Punan Hospital Shanghai 200125 China

**Keywords:** cardiac fibroblast, cardiac fibrosis, cardiomyocyte, EP4 receptor, isoproterenol, TGF‐β signaling

## Abstract

Sustained β‐adrenergic activation induces cardiac fibrosis characterized by excessive deposition of extracellular matrix (ECM). Prostaglandin E_2_ (PGE_2_) receptor EP4 is essential for cardiovascular homeostasis. This study aims to investigate the roles of cardiomyocyte (CM) and cardiac fibroblast (CF) EP4 in isoproterenol (ISO)‐induced cardiac fibrosis. By crossing the EP4^f/f^ mice with α‐MyHC‐Cre or S100A4‐Cre mice, this work obtains the CM‐EP4 knockout (EP4^f/f^‐α‐MyHC^Cre+^) or CF‐EP4 knockout (EP4^f/f^‐S100A4^Cre+^) mice. The mice of both genders are subcutaneously injected with ISO (5 mg kg^−1^ day^−1^) for 7 days. Compared to the control mice, both EP4^f/f^‐α‐MyHC^Cre+^ and EP4^f/f^‐S100A4^Cre+^ mice show a significant improvement in cardiac diastolic function and fibrosis as assessed by echocardiography and histological staining, respectively. In the CMs, inhibition of EP4 suppresses ISO‐induced TGF‐β1 expression via blocking the cAMP/PKA pathway. In the CFs, inhibition of EP4 reversed TGF‐β1‐triggers production of ECM via preventing the formation of the TGF‐β1/TGF‐β receptor complex and blocks CF proliferation via suppressing the ERK1/2 pathway. Furthermore, double knockout of the CM‐ and CF‐EP4 or administration of EP4 antagonist, grapiprant, markedly improves ISO‐induced cardiac diastolic dysfunction and fibrosis. Collectively, this study demonstrates that both CM‐EP4 and CF‐EP4 contribute to β‐adrenergic activation‐induced cardiac fibrosis. Targeting EP4 may offer a novel therapeutic approach for cardiac fibrosis.

## Graphical abstract

This study explores the roles of cardiomyocyte‐ and cardiac fibroblast‐specific EP4 in isoproterenol‐induced cardiac fibrosis. The findings reveal that EP4 inhibition improves diastolic function and reduces fibrosis by modulating TGF‐β1 signaling and extracellular matrix protein production. Notably, genetic or pharmacological targeting of EP4 offers a new means to treat β‐adrenergic activation‐induced cardiac fibrosis.

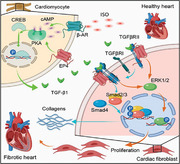

## 1 Introduction

Cardiovascular diseases (CVDs) are major threats of health and causes of death worldwide.^[^ ^1^, ^2^ ^]^ Cardiac fibrosis is a common pathological condition that plays a crucial role in various heart diseases and is characterized by the excessive accumulation of extracellular matrix (ECM), primarily collagens, in the cardiac tissue.^[^ ^3^ ^]^ This pathological remodeling impacts the mechanical, electrical, and vasomotor function of the heart, leading to compromised cardiac output and increased risk of arrhythmias and heart failure.^[^ ^4^ ^]^ Despite its significance, the precise mechanisms driving cardiac fibrosis remain partially understood, posing a significant barrier to develop targeted therapies.^[^ ^5^ ^]^ Recent advances have begun to unravel the complex interplay between cardiac fibroblasts, myocytes, and the ECM in heart disease. These studies have highlighted several key pathways and mediators, such as transforming growth factor‐beta (TGF‐β), angiotensin II and platelet‐derived growth factor (PDGF) that modulate fibroblast function and fibrosis.^[^ ^5^, ^6^ ^]^ Sympathetic nervous system activation is an important compensatory mechanism to support cardiac systolic function, but prolonged overactivation leads to cardiac remodeling, including cardiac hypertrophy and fibrosis.^[^ ^7^ ^]^ Increasing evidence demonstrates that sympathetic hyperactivity activates β‐adrenergic receptors (β‐ARs) in the cardiomyocytes (CMs), triggering acute cardiac inflammation, cardiac injury, myocardial ischemia, and cardiac remodeling.^[^ ^8^ ^]^ Cardiac fibrosis is an adaptive response upon β‐adrenergic activation, while excessive ECM deposition in myocardial interstitium eventually causing cardiac dysfunction, especially reduction of left ventricular diastolic function.^[^ ^7^, ^9^ ^]^ However, there is no significant association between early β‐blocker use and in‐hospital mortality in patients with stress cardiomyopathy, a disease related to elevated catecholamine.^[^ ^10^ ^]^ Therefore, the underlying mechanisms of acute β‐adrenergic activation‐induced cardiac remodeling remain incompletely understood.

Increasing evidence demonstrates that prostaglandin E_2_ (PGE_2_) plays an essential role in cardiac physiology and pathophysiology. Nonsteroidal anti‐inflammatory drugs (NSAIDs) are widely used to ameliorate pain, fever, and inflammation by targeting cyclooxygenases (COX‐1 and COX‐2), which catalyzes arachidonic acid into prostaglandins.^[^ ^11^ ^]^ Although gastrointestinal complications are minimalized, Coxibs, the COX‐2 selective NASIDs, have been found in clinical practice to increase cardiovascular events, including myocardial infarction and hypertension.^[^ ^12^ ^]^ It is generally believed that decreased production of prostacyclin (PGI_2_) and PGE_2_, two predominant PGs, accounts for the side effect of hypertension.^[^ ^13^ ^]^ A series of studies have also reported that inhibition or deletion of COX‐2 and mPGES‐1 (the dominant terminal PGE_2_ synthase) are detrimental to the heart.^[^ ^14^, ^15^, ^16^, ^17^ ^]^ These findings strongly suggest that inhibition of the PGE_2_ signaling contributes to Coxibs’ cardiac side effects and PGE2 plays an important role in cardiac remodeling.

It has been previously reported that PGE_2_ and its four G‐protein coupled receptors (EP1‐4) are essential in cardiac homeostasis. For example, the PGE_2_‐EP1 axis protects cardiomyocytes from doxorubicin (DOX)‐induced ferroptosis by activating the PKC‐Nrf2 signaling.^[^ ^18^ ^]^ Depletion of EP2 significantly impairs macrophage recruitment to the injured myocardium, thereby attenuating cardiac tissue repair.^[^ ^19^ ^]^ Activation of EP3 in macrophages facilitates cardiac healing after myocardial infarction.^[^ ^20^ ^]^ In terms of EP4, it has been found that EP4 exerts both protective and detrimental effects in the ischemic heart disease.^[^ ^21^ ^]^ EP4 was also found to be responsible for PGE_2_‐induced cardiac hypertrophy through the ERK1/2‐Stat3 pathway^[^ ^22^ ^]^ and the EP4 specific antagonist blocked the hypertrophic actions of PGE_2_ by suppressing the expression of ANP and BNP in neonatal cardiac cells.^[^ ^23^ ^]^ In contrast, other studies showed that the EP4 agonist ONO‐0260164 inhibited pressure overload‐induced cardiac fibrosis in a transverse aortic constriction (TAC) model.^[^ ^24^ ^]^ Similarly, it has been recently reported that EP4 in cardiac microvascular endothelial cells inhibits endothelial‐mesenchymal transition (EndMT) to attenuate isoproterenol (ISO)‐induced cardiac fibrosis in rats.^[^ ^25^ ^]^ Although the reason for the discrepancy remains unclear,^[^ ^26^ ^]^ these findings suggest that EP4 is involved in the pathogenesis of cardiac remodeling, especially cardiac fibrosis.

In this study, to characterize the role and mechanism of EP4 in cardiac fibrosis, we crossed the EP4‐flox/flox (EP4^f/f^) mice with α‐MyHC‐Cre mice or S100A4‐Cre mice to generate cardiomyocyte (CM)‐ and cardiac fibroblast (CF)‐specific EP4 gene knockout mice and treated them with subcutaneous injection of ISO for 7 days to induce cardiac fibrosis. We found that both CM‐ and CF‐specific EP4 gene knockout mice exhibited improved cardiac diastolic function and fibrosis. We also provide evidence that pharmacological inhibition of EP4 also markedly attenuates cardiac fibrosis.

## 2 Results

### 2.1 EP4 Expression is Upregulated in ISO‐Treated Mouse Heart

To investigate whether EP4 participates in ISO‐induced cardiac fibrosis, wild‐type mice were subcutaneously injected with ISO for 7 days (Figure S1A, Supporting Information). As expected, ISO treatment resulted in a marked increase in heart weight (HW) and left ventricular mass (LV mass) (Figure S1B–D, Supporting Information), with more severe heart stiffness as revealed by raised early‐to‐atrial wave ratio (E/A ratio) (Figure S1E, Supporting Information). ISO treatment also significantly increased the mRNA levels of collagen I and collagen III (Figure S1F, Supporting Information). Consistent with a previous report,^[^ ^27^ ^]^ the expression of cyclooxygenase‐2 (COX‐2), the inducible enzyme that converts arachidonic acid into prostaglandins, was significantly increased (Figure S1G, Supporting Information). Further studies revealed that ISO specifically enhanced PGE_2_ production in the CMs, rather than in the CFs (Figure S1H,I, Supporting Information). Similarly, the mRNA levels of EP4, one of the PGE_2_ receptor, were markedly increased (Figure S1J, Supporting Information). Furthermore, immunohistochemical staining revealed that the EP4 protein expression was upregulated in both CMs and CFs (Figure S1K, Supporting Information). In addition, we generated a fibroblast‐specific lineage tracing mouse by crossing the Rosa26‐tdTomato mouse with the S100A4‐Cre mouse (Figure S1L, Supporting Information), which further demonstrated that EP4 protein levels were increased in both CMs and CFs following ISO treatment (Figure S1M, Supporting Information). These findings suggest that elevated EP4 expression may play an important role in ISO‐induced cardiac fibrosis.

### 2.2 Deletion of the CM‐EP4 Gene Improves ISO‐Induced Cardiac Diastolic Dysfunction and Fibrosis

Since EP4 was significantly upregulated in the CMs after ISO treatment, we therefore investigated whether CM‐EP4 deficiency affects ISO‐induced cardiac dysfunction and fibrosis. By crossing the EP4‐flox/flox mice (EP4^f/f^) with α‐MyHC‐Cre mice, we successfully generated a CM‐EP4 gene specific knockout mouse line (EP4^f/f^‐α‐MyHC^Cre+^) (Figure S2A, Supporting Information). Both PCR‐based genotyping and immunofluorescence assay demonstrate that the EP4 gene was substantially deleted (Figure S2B,C, Supporting Information).

**Figure 1:**
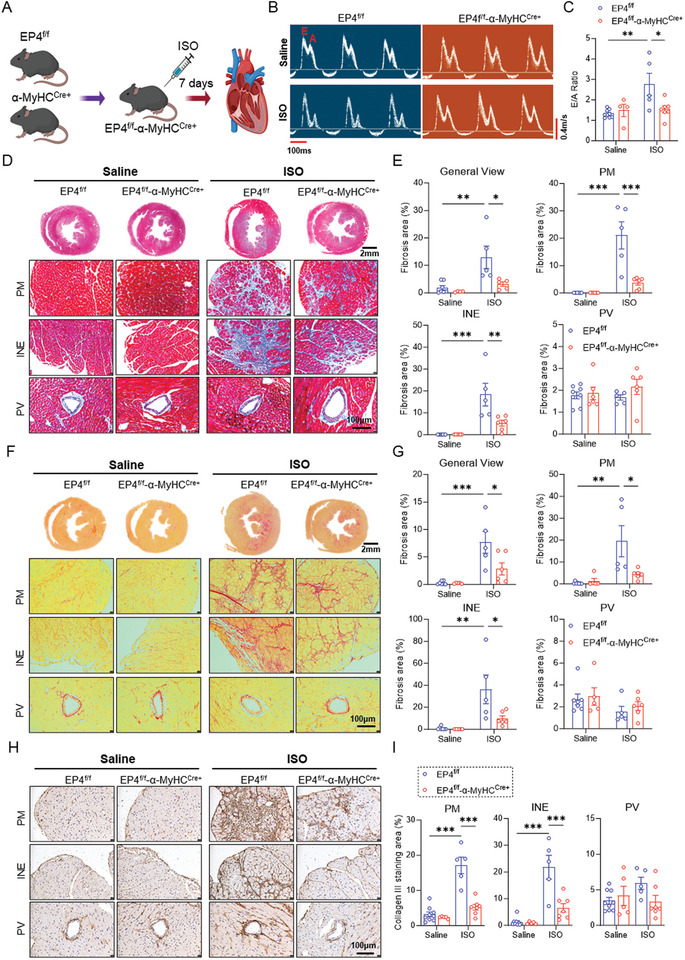
Deletion of the CM‐EP4 improves ISO‐induced cardiac diastolic dysfunction and fibrosis. A) The experimental scheme showing the generation of EP4^f/f^‐α‐MyHC^Cre+^ (CM‐EP4^−/−^) mice and ISO‐induced cardiac fibrosis model. The EP4^f/f^‐α‐MyHC^Cre+^ (CM‐EP4^−/−^) mice and control mice (EP4^f/f^) were generated by crossing the EP4^f/f^ mice with the α‐MyHC^Cre+^ mice. Male mice aged 8–10 weeks were administered subcutaneous injections of ISO daily for 7 consecutive days to induce cardiac fibrosis. B&C. Representative images of peak velocity flow in early diastole (E, m/s) to peak velocity flow in late diastole by atrial contraction (A, m/s) in the EP4^f/f^ and EP4^f/f^‐α‐MyHC^Cre+^ mice injected with saline (top panels) or ISO (bottom panels) for 7 days (B). The ratio of E/A was calculated (C). Transverse scale bar = 100 ms. Vertical scale bar = 0.4 m s^−1^. *n* = 4–9 mice per group. D&E. Representative images of Masson's trichrome staining of the hearts of the EP4^f/f^ and EP4^f/f^‐α‐MyHC^Cre+^ mice receiving saline (left panels) or ISO (right panels) treatment for 7 days. General view, papillary muscle (PM) area, interstitial area near the endocardium (INE), and perivascular (PV) area were shown, respectively. Quantitative analysis of fibrotic areas (blue) was performed by image J software. General view scale bar = 2mm. Enlarged picture scale bar = 100 µm. *n* = 4 to 7 per group. F&G. Representative images of Sirius red staining of the hearts of the EP4^f/f^ and EP4^f/f^‐α‐MyHC^Cre+^ mice treated with saline (left panels) or ISO (right panels) for 7 days. General view, papillary muscle (PM) area, interstitial area near the endocardium (INE), and perivascular (PV) area were shown, respectively. Quantitative analysis of fibrotic areas (red) was performed by image J software. General view scale bar = 2mm. Enlarged picture scale bar = 100 µm. *n* = 4 to 7 per group. H&I. Representative images of immunohistochemical staining of collagen III in heart tissues of the EP4^f/f^ and EP4^f/f^‐α‐MyHC^Cre+^ mice after saline (left panels) or ISO (right panels) treatment for 7 days. Papillary muscle (PM) area, interstitial area near the endocardium (INE), and perivascular (PV) area were shown, respectively. Quantitative analysis of collagen III positive areas was performed by image J software (I). Scale bar = 100 µm. *n* = 5 to 9 per group. Data were presented as mean±SEM. *p < 0.05, **p < 0.01, ***p < 0.001 by two‐way ANOVA followed by the Tukey's multiple comparisons test (C, E, G and I).

After ISO injection for 7 days (**Figure** **1**A), we observed a significant decrease in cardiac diastolic dysfunction as revealed by a reduced E/A ratio in the EP4^f/f^‐α‐MyHC^Cre+^ mice compared to the EP4^f/f^ mice in male gender (Figure 1B,C). Histological examination using Masson trichrome staining and Sirus red staining showed that the ECM accumulation in the papillary muscle (PM) area and the interstitial area near the endocardium (INE) was markedly ameliorated in the EP4^f/f^‐α‐MyHC^Cre+^ mice after ISO treatment. Little ECM was deposited in the perivascular (PV) area, and the amounts were similar between two groups (Figure 1D–G). Consistently, the upregulation of collagen I and collagen III mRNA and protein levels induced by ISO was significantly diminished in the EP4^f/f^‐α‐MyHC^Cre+^ mice, particularly for collagen III in the PM and INE areas (Figure 1H,I; Figure S3A–D, Supporting Information). However, the heart weight/body weight (HW/BW) ratio and heart weight/tibia length (HW/TL) ratio, LV mass, left ventricular anterior wall thickness (LVAW), and left ventricular posterior wall thickness (LVPW) were similar between the EP4^f/f^ and EP4^f/f^‐α‐MyHC^Cre+^ mice (Figure S4A–H, Supporting Information). In addition, the cardiac systolic function, as reflected by ejection fraction (EF), fractional shortening (FS), left ventricular internal diameter (LVID), and left ventricular volume (LV vol) was comparable between two genotypes (Figure S4I–N, Supporting Information). To examine whether there is a gender difference in cardioprotective effect of the CM‐EP4 gene ablation, female EP4^f/f^ and EP4^f/f^‐α‐MyHC^Cre+^ mice were also injected with ISO for 7 days. Similar to the findings observed in male mice, CM‐EP4 gene ablation effectively mitigated ISO‐induced cardiac diastolic dysfunction (Figure S5A,B, Supporting Information) and fibrosis (Figure S5C–F, Supporting Information) in female mice, with minimal impact on cardiac systolic function and hypertrophy (Figure S6A–N, Supporting Information). Collectively, these results demonstrate that deletion of the CM‐EP4 gene ameliorates ISO‐induced cardiac diastolic dysfunction and cardiac fibrosis in both male and female mice.

### 2.3 Deletion of the CF‐EP4 Gene Ameliorates ISO‐Induced Left Ventricular Diastolic Dysfunction and Fibrosis

**Figure 2:**
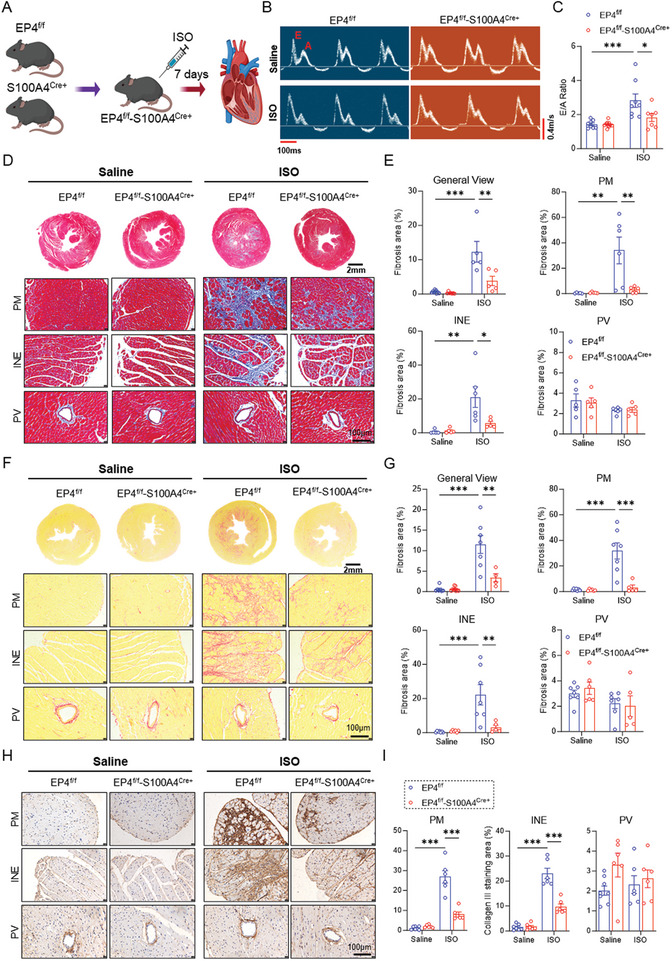
Deletion of the CF‐EP4 ameliorates ISO‐induced left ventricular diastolic dysfunction and fibrosis. A. The experimental scheme showing the generation of EP4^f/f^‐S100A4^Cre+^ (CF‐EP4^−/−^) mice and ISO‐induced cardiac fibrosis model. The EP4^f/f^‐S100A4^Cre+^ mice and control mice (EP4^f/f^) were generated by crossing the EP4^f/f^ mice and S100A4^Cre+^ mice. Male mice aged 8–10 weeks were subjected to subcutaneous injections of ISO for 7 days to induce cardiac fibrosis. B,C) Representative images of peak velocity flow in early diastole (E, m/s) to peak velocity flow in late diastole by atrial contraction (A, m/s) in mice injected with saline (top panels) or ISO (bottom panels) for 7 days (B). The ratio of E/A was calculated to reveal the cardiac diastolic function (C). Transverse scale bar = 100ms. Vertical scale bar = 0.4 m s^−1^. *n* = 7–10 mice per group. D,E) Representative images of Masson's trichrome staining of the hearts of the EP4^f/f^ and EP4^f/f^‐S100A4^Cre+^ mice treated with saline (left panels) or ISO (right panels) for 7 days. General view, papillary muscle (PM) area, interstitial area near the endocardium (INE), and perivascular (PV) area were shown, respectively. Quantitative analysis of fibrotic areas (blue) was performed by image J software. Scale bar = 100 µm. *n* = 5–9 per group. F,G) Representative images of Sirius red staining of the hearts of the EP4^f/f^ and EP4^f/f^‐S100A4^Cre+^ mice treated with saline (left panels) or ISO (right panels) for 7 days. General view, papillary muscle (PM) area, interstitial area near the endocardium (INE), and perivascular (PV) area were shown, respectively. Quantitative analysis of fibrotic areas (red) was performed by image J software. Scale bar = 100 µm. *n* = 5–9 per group. H&I. Representative images of immunohistochemical staining of collagen III in the hearts of the EP4^f/f^ and EP4^f/f^‐S100A4^Cre+^ mice receiving saline (left panels) or ISO (right panels) treatment for 7 days. Papillary muscle (PM) area, interstitial area near the endocardium (INE), and perivascular (PV) area were shown, respectively. Quantitative analysis of collagen III positive areas was performed by image J software. Scale bar = 100 µm. *n* = 5–9 per group. Data were presented as mean±SEM. *p < 0.05, **p < 0.01, ***p < 0.001 by two‐way ANOVA followed by the Tukey's multiple comparisons test (C,E,G,I).

To clarify the role of the CF‐EP4 in ISO‐induced cardiac fibrosis, we crossed the EP4^f/f^ mice with S100A4‐Cre mice to generate a CF‐EP4 gene specific knockout mouse line (EP4^f/f^‐S100A4^Cre+^) (Figure S7A, Supporting Information). By using the specific primer pairs (i.e., F1 and R2) (Figure S7A, Supporting Information), we demonstrated that the EP4^f/f^‐S100A4^Cre+^ mouse exhibited a predicted 370bp fragment of recombined EP4 allele (Figure S7B, Supporting Information). Immunofluorescence analysis showed that EP4 was slightly reduced in the cardiac interstitium of the EP4^f/f^‐S100A4^Cre+^ mouse heart (Figure S7C, Supporting Information). This reduction may be attributed to the relatively small proportion of S100A4‐positive cells present in the heart under basal conditions, as demonstrated by the Rosa26‐tdTomato lineage tracing assay (Figure S7D, Supporting Information). Both male EP4^f/f^ mice and EP4^f/f^‐S100A4^Cre+^ mice were received ISO or saline injection for 7 days (**Figure** **2**A). The results showed that both the HW/BW ratio and HW/TL ratio increased after ISO treatment in both genotypes, with no significant difference observed between the two groups (Figure S8A,B, Supporting Information). Echocardiography measurement showed that although ISO treatment led to a marked increase in LV mass, LVAW, and LVPW in both genotypes, no significant change was found between the two groups of mice (Figure S8C–H, Supporting Information). Also, ISO treatment did not change the systolic function in both EP4^f/f^ and EP4^f/f^‐S100A4^Cre+^ mice, as reflected by EF, FS, LVID, and LV vol (Figure S8I–N, Supporting Information).

Importantly, the decline in left ventricular diastolic function induced by ISO was significantly mitigated in the EP4^f/f^‐S100A4^Cre+^ mice compared to the EP4^f/f^ mice, as evidenced by the E/A ratio (Figure 2B,C). We also assessed ECM contents using Masson trichrome staining (Figure 2D,E) and Sirus red staining in male mice (Figure 2F,G). Consistent with the cardiac function changes, the ECM protein accumulation in the PM and the INE area was significantly attenuated in the EP4^f/f^‐S100A4^Cre+^ mice compared to that in the EP4^f/f^ mice (Figure 2D–G). Further studies revealed that ISO‐induced cardiac collagen I and collagen III mRNA expression was significantly attenuated in the EP4^f/f^‐S100A4^Cre+^ mice compared to that in the EP4^f/f^ mice (Figure S9A,B, Supporting Information). Consistently, immunohistochemical staining revealed that ISO‐induced collagen I and collagen III protein expression was markedly improved in the PM area and INE area in the EP4^f/f^‐S100A4^Cre+^ mice (Figure 2H,I; Figure S9C,D, Supporting Information). Consistent with the results seen in male mice, the ablation of CF‐EP4 gene significantly alleviated ISO‐induced cardiac diastolic dysfunction (Figure S10A,B, Supporting Information) and fibrosis (Figure S10C–F, Supporting Information) in female mice, while exerting little effect on cardiac systolic function and hypertrophy (Figure S11A–N, Supporting Information). Together, these results demonstrate that ablation of the CF‐EP4 gene suppresses ISO‐induced cardiac fibrosis in both genders.

### 2.4 EP4 Promotes ISO‐Induced TGF‐β1 Expression in the CMs via the PKA Pathway

**Figure 3:**
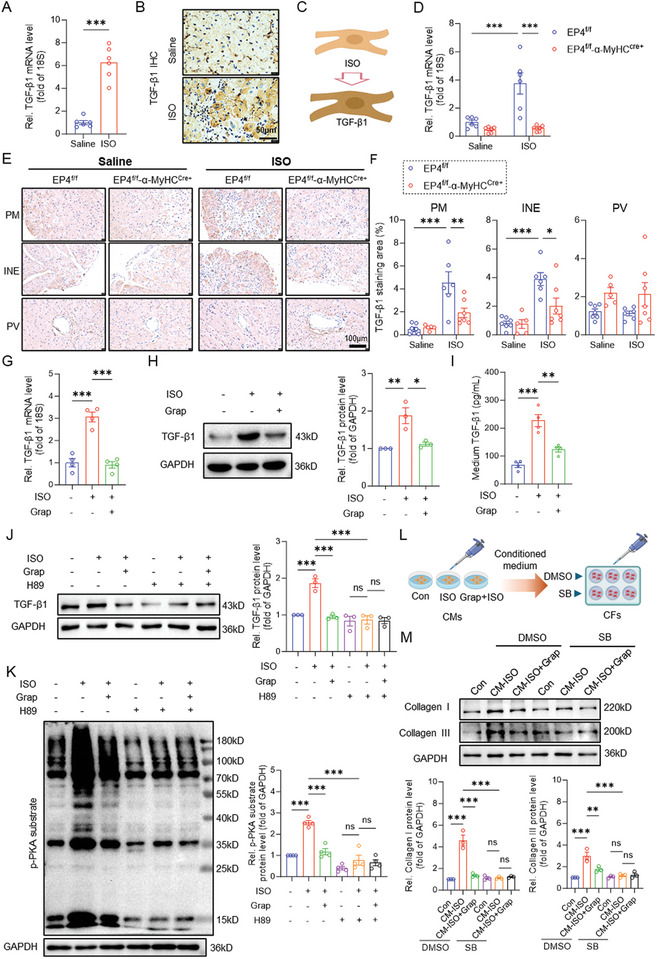
EP4 promotes ISO‐induced TGF‐β1 expression in the CMs via the PKA pathway. A) qRT‐PCR analysis showing upregulation of the mRNA expression of TGF‐β1 in wild‐type mouse heart tissues after ISO treatment for 7 days. *n* = 6 per group. B,C) Representative images of immunohistochemical staining of TGF‐β1 in wild‐type mouse heart tissues after ISO treatment for 7 days (B). Schematic showing of ISO‐induced TGF‐β1 expression in the CMs (C). Scale bar = 50 µm. D) qRT‐PCR analysis of the mRNA expression of TGF‐β1 in the hearts of the EP4^f/f^ and EP4^f/f^‐α‐MyHC^Cre+^ mice receiving ISO treatment for 7 days. *n* = 6 per group. E,F) Representative images of immunohistochemical staining of TGF‐β1 in the hearts of the 8–10 weeks old male EP4^f/f^ and EP4^f/f^‐α‐MyHC^Cre+^ mice receiving saline (left panel) or ISO (right panel) treatment for 7 days. Papillary muscle (PM) area, interstitial area near the endocardium (INE), and perivascular (PV) area were shown, respectively (E). Quantitative analysis of TGF‐β1 positive areas was performed by image J software (F). Scale bar = 100 µm. *n* = 5–7 per group. G) qRT‐PCR analysis the mRNA expression of TGF‐β1 in primary cultured neonatal rat cardiomyocytes (NRCMs) treated with ISO (20 µM) for 24 h. *n* = 4. H) Western blot assay demonstrating that pre‐treatment of NRCMs with grapiprant (1µM) for 30 min markedly reduced ISO‐induced TGF‐β1 protein expression. The quantitative analysis of TGF‐β1 protein level was performed by image J software. *n* = 3. I) Pre‐treatment of the NRCMs with grapiprant (1µM) suppressed ISO‐induced TGF‐β1 protein secretion in the medium, as assessed by an ELISA kit. *n* = 4. J) Western blot assay showing the effect of the PKA inhibitor H89 on ISO‐induced TGF‐β1 protein expression in the NRCMs. The cells were pretreated with H89 (20 µM) and/or grapiprant (1 µM) for 30 min and followed by ISO (20µM) treatment for 24 h. The quantitative analysis of the proteins level was performed by image J software. *n* = 3. K) Western blot analysis showing the effect of grapiprant (1µM) on ISO‐elicited phosphorylated PKA substrate protein levels in the presence or absence of H89. For the p‐PKA substrate blot, protein markers were clearly labeled. *n* = 4. L) The experimental scheme showing that the NRCMs were pretreated with or without grapiprant (1µM) for 30 min and then treated with ISO for 24 h. After ISO treatment, the supernatants were collected as conditional medium. The conditional medium was used to treat the NRCFs, which had received a 30‐min pretreatment with either DMSO or SB431542 (10µM), for 24 h. M) Western blot assay demonstrating the effect of 24‐h incubation of conditional medium from ISO‐exposed NRCMs (CM‐ISO) or ISO‐treated NRCMs pretreated with grapiprant (CM‐ISO+Grap) on the expression of collagen I, collagen III, and TGF‐β1 in the NRCFs receiving 30‐min pretreatment of DMSO or SB431542 (10 µM). The quantitative analysis of the protein levels was performed by image J software. *n* = 3. Data were presented as mean±SEM. *p < 0.05, **p < 0.01, ***p < 0.001 by two‐tailed unpaired *t*‐test (A) or by two‐way ANOVA followed by the Tukey's multiple comparisons test (D,F) or by one‐way ANOVA followed by the Tukey's multiple comparisons test (G–K,M).

Since TGF‐β1 is the master regulator of fibrosis, we first examined TGF‐β1 expression in wild‐type mouse hearts after ISO injection. Quantitative RT‐PCR and immunoblot assays revealed that the expression of TGF‐β1 mRNA and protein was significantly increased after ISO treatment (**Figure** **3**A,B). TGF‐β1 protein was specifically upregulated in the CMs rather than in the CFs (Figure 3B), suggesting that ISO‐induced TGF‐β1 expression mainly occurs in the CMs (Figure 3C). Next, we determined the effect of EP4 on TGF‐β1 expression in the CMs and found that deletion of the EP4 gene in the CMs almost completely abolished ISO‐induced TGF‐β1 expression at both mRNA and protein levels (Figure 3D–F), especially in the PM and INE areas (Figure 3E). Consistently, in vitro studies indicated that ISO treatment markedly increased TGF‐β1 mRNA and protein expression in primary cultured neonatal rat cardiomyocytes (NRCMs), which was significantly blocked by the EP4 antagonist grapiprant and MF498 (Figure 3G,H; Figure S12A,B, Supporting Information). More importantly, administration of ISO notably increased the TGF‐β1 levels in the NRCMs medium, while pretreatment with grapiprant effectively counteracted this effect (Figure 3I). Moreover, we found that H89, a PKA inhibitor, blocked the ISO‐induced TGF‐β1 expression in the CMs (Figure 3J). Besides, we found that deletion of CM‐EP4 suppressed PKA activation as revealed by less phosphorylation of PKA substrates (Figure S12C,D, Supporting Information). These results suggesting that ISO induces TGF‐β1 expression in an PKA‐dependent manner. Furthermore, both H89 and grapiprant treatment significantly attenuated ISO‐induced PKA substrate phosphorylation (Figure 3K). The cAMP‐response element‐binding protein (CREB), a well‐known target of PKA, has been reported to act as a transcription factor for TGF‐β1.^[^ ^28^, ^29^ ^]^ In our study, we observed that the active form of CREB (phosphorylated CREB) was significantly reduced in the isolated CMs of EP4^f/f^‐α‐MyHC^Cre+^ mice (Figure S12C,D, Supporting Information). Additionally, grapiprant remarkably suppressed ISO‐induced CREB activation, and this effect was completely abolished by H89 in the NRCMs (Figure S12E,F, Supporting Information). Moreover, the expression of β‐AR in the CMs remained unaffected by EP4 deletion or inhibition (Figure S13A–D, Supporting Information). These results indicate that EP4 increases TGF‐β1 expression in the CMs via the cAMP/PKA/CREB pathway.

To clarify whether EP4 also regulates TGF‐β1 expression in the CFs, we determined the effect of the EP4 on TGF‐β1 expression both in vivo and in vitro. The results showed that specific deletion of the CF‐EP4 gene had no effect on ISO‐induced cardiac TGF‐β1 expression at both mRNA and protein levels (Figure S14A–C, Supporting Information). The CF‐EP4 gene deficiency failed to suppress ISO‐induced TGF‐β1 expression in the CMs (Figure S14B,C, Supporting Information). Consistent with the in vivo observations, ISO treatment had no effect on TGF‐β1 and collagen I and III expression in the primary cultured neonatal rat cardiac fibroblasts (NRCFs). Pretreatment with the EP4 antagonist grapiprant did not alter the TGF‐β1 expression levels in the NRCFs exposed to ISO (Figure S14D, Supporting Information). However, we found that the expression of collagen I and collagen III was markedly increased in the CFs by the conditional medium from ISO‐treated CMs (CM‐ISO), but not by the medium from CMs treated with ISO and grapiprant simultaneously (CM‐ISO+Grap) (Figure 3L,M). Importantly, pretreatment of the CFs with SB431542, a TGF‐β1 receptor kinase inhibitor, abolished CM‐ISO driven collagen biosynthesis and CM‐ISO+Grap had no further inhibitory effect on the collagen production (Figure 3M). Taken together, these findings demonstrate that ISO induces TGF‐β1 expression in the CMs rather than in the CFs. TGF‐β1 produced by the CMs is the predominant factor that triggers ECM production in the CFs.

### 2.5 Blockade of EP4 Suppresses TGF‐β1‐Mediated Profibrotic Signaling in Cultured CFs

**Figure 4:**
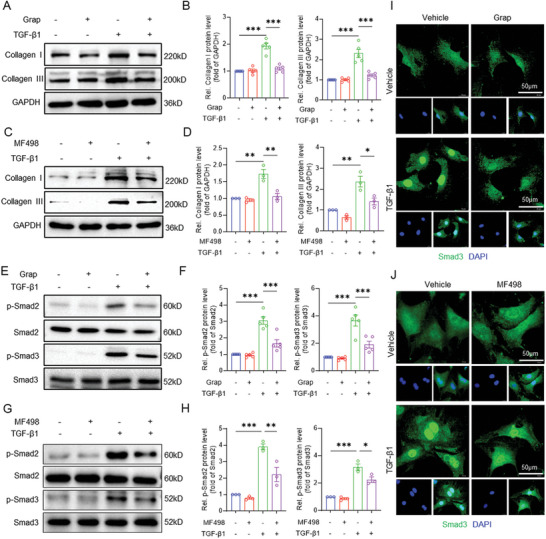
Blockade of EP4 suppresses TGF‐β1 mediated collagen production in the CFs by inhibiting phosphorylation of Smad2/3. A,B) Western blot analysis showing the effect of 24‐h TGF‐β1 (10 ng mL^−1^) treatment on collagen I and collagen III expression in primary cultured neonatal rat cardiac fibroblasts (NRCFs) pretreated with or without grapiprant (1µM) for 30 min (A). Quantitative analysis was performed using image J software (B). *n* = 5. C,D) Western blot analysis demonstrating the effect of 24‐h TGF‐β1 (10 ng mL^−1^) treatment on collagen I and collagen III expression in the NRCFs pretreated with or without MF498 (0.1µM) for 30 min (C). Quantitative analysis was performed using image J software (D). *n* = 3. E,F) Western blot analysis showing the effect of 3‐h TGF‐β1 (10 ng mL^−1^) treatment on phosphorylation levels of Smad2 and Smad3 in the NRCFs pretreated with or without grapiprant (1µM) for 30 min (E). Quantitative analysis was performed using image J software (F). *n* = 5. G,H) Immunoblot analysis demonstrating the effect of 3‐h TGF‐β1 (10 ng mL^−1^) treatment on phosphorylation levels of Smad2 and Smad3 in the NRCFs pretreated with or without MF498 (0.1µM) for 30 min (G). Quantitative analysis was performed using image J software (H). *n* = 5. I,J) Immunofluorescence staining showing the effect of 3‐h TGF‐β1 (10 ng mL^−1^) treatment on Smad3 (green) nuclear translocation in the NRCFs pretreated with or without grapiprant (1µM) (I) or MF498 (0.1µM) (J) for 30 min. DAPI (blue) staining showed the nucleus. Scale bar = 50 µm. Data were presented as mean±SEM. *p < 0.05, **p < 0.01, ***p < 0.001 by one‐way ANOVA followed by the Tukey's multiple comparisons test (B,D,F,H).

To characterize the role of EP4 in the CFs, we examined whether EP4 influences TGF‐β1‐induced fibrotic process. Treatment of TGF‐β1 dramatically increased collagen I and collagen III production in primary cultured NRCFs, which was significantly reversed by pretreatment with the EP4 antagonists, grapiprant and MF498 (**Figure** **4**A–D). Similarly, TGF‐β1‐triggered phosphorylation of Smad2 and Smad3 was remarkably blocked by grapiprant and MF498 (Figure 4E–H). Moreover, immunofluorescence studies showed that TGF‐β1‐triggered Smad3 nuclear translocation was abolished by pretreatment with grapiprant or MF498 (Figure 4I,J). In contrast, activation of EP4 by CAY10580 and PGE1‐OH, two selective EP4 agonists, facilitated TGF‐β1‐induced Smad3 phosphorylation and nuclear translocation (Figure S15A–F, Supporting Information). Collectively, these results indicate that EP4 activates the TGF‐β1‐Smad2/3 pathway to increase ECM production in the CFs.

### 2.6 EP4 Directly Interacts with the TGFBRII in the CFs

**Figure 5:**
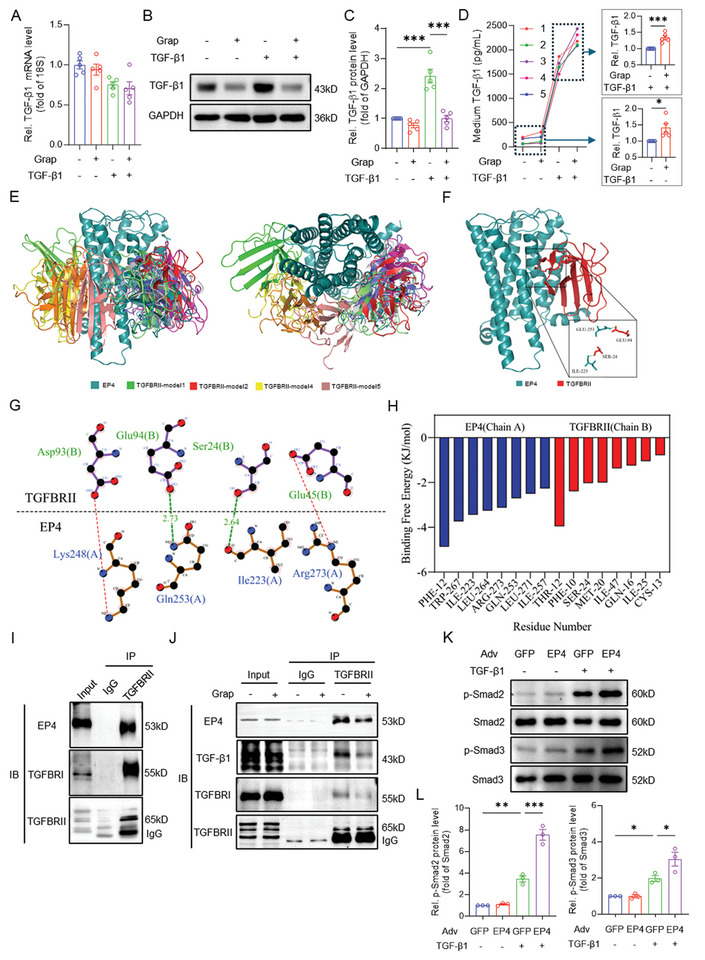
EP4 augments the TGF‐β1 signaling by the formation of the EP4‐TGFBRII complex. A) qRT‐PCR analysis demonstrating the effect of 24‐h TGF‐β1 (10 ng mL^−1^) treatment on the mRNA expression of TGF‐β1 in the NRCFs pretreated with or without grapiprant (1µM) for 30 min. *n* = 3. B,C) Western blot assay showing the effect of 24‐h TGF‐β1 (10 ng mL^−1^) treatment on the protein expression of TGF‐β1 in the NRCFs pretreated with or without grapiprant (1µM) for 30 min (B). Quantitative analysis was performed using image J software (C). *n* = 5. D. ELISA measurement of TGF‐β1 levels in the medium of cultured NRCFs. The cells were pretreated with or without grapiprant (1µM) for 30 min and then treated with TGF‐β1 (10 ng mL^−1^) for 24 h. The relative contents of TGF‐β1 in the medium were calculated under basal and TGF‐β1‐treated conditions. *n* = 5. E–H) The top ten binding modes of the EP4‐TGFBRII complex. The left image is the front view and the right image is the top view (E). The snug‐fit‐in model of last frame of the EP4‐TGFBRII complex model 2 was also highlighted. The cyan and red cartoon structures represent EP4 and TGFBRII protein. The green dash lines represent possible hydrogen bonding (F). 2D interactions of the EP4‐TGFBRII complex. The green dot lines connect the amino acid residues that undergo putative hydrogen bonding. The red dot lines represent hydrophobic effects such as salt bridging (G). The analysis of binding free energy contribution (MM/PBSA model) from the key residues of the EP4‐TGFBRII complex. The blue bars represent the binding free energy contribution of EP4 residues and the red bars represent the binding free energy contribution of TGFBRII residues (H). I) Immunoprecipitation (IP) assay showing that EP4 and TGFBRII have a physical interaction. The NRCFs were harvested in modified RIPA buffer and cell lysates were immunoprecipitated with a mouse anti‐TGFBRII antibody. The resultant immunoprecipitated proteins were blotted with a rabbit anti‐EP4 antibody, rabbit anti‐TGFBRI antibody and mouse anti‐TGFBRII antibody. J) IP assay showing that the physical interaction of EP4 and TGFBRII is attenuated by the EP4 antagonist grapiprant. The NRCFs were pretreated with grapiprant (1µM) for 30 min, followed by the treatment with TGF‐β1 (10 ng mL^−1^) for 3 h. The cells were harvested in modified RIPA buffer and cell lysates were immunoprecipitated with a mouse anti‐TGFBRII antibody. The resultant immunoprecipitated proteins were blotted with a rabbit anti‐EP4, anti‐TGF‐β1, and TGFBRI antibody, and mouse anti‐TGFBRII antibody. K,L) Western blot analysis demonstrating the effect of 3‐h TGF‐β1 (10 ng mL^−1^) treatment on the levels of total and phosphorylated Smad2 and Smad3 in the NRCFs infected with GFP‐adenoviruses or EP4‐adenoviruses (25MOI) for 24 h (K). Quantitative analysis was performed using image J software (L). *n* = 3. Data were presented as mean±SEM. *p < 0.05, **p < 0.01, ***p < 0.001 by one‐way ANOVA followed by the Tukey's multiple comparisons test (A,C,L) or by two‐tailed unpaired *t*‐test (D).

To explore the underlying mechanism by which EP4 antagonism blocks TGF‐β1‐induced Smad2/3 activation, we first checked the classic signaling pathways of EP4. We found that neither forskolin nor H‐89 affected TGF‐β1‐induced phosphorylation of Smad2 and Smad3 (Figure S16A, Supporting Information). Pretreatment with the MAPK pathway inhibitors (i.e., SP600125 for JNK, Adezmapimod for p38, and PD98059 for ERK1/2) or the AKT inhibitor MK‐2206 also unaffected TGF‐β1‐induced Smad2 and Smad3 phosphorylation (Figure S16B,C, Supporting Information). These results suggest that the inhibitory effect of EP4 antagonism on TGF‐β1‐mediated Smad2/3 activation is not achieved through its classical pathways such as the PKA, MAPK, and AKT signaling. Although TGF‐β1 and grapiprant along or in combination did not change the mRNA levels of TGF‐β1 (**Figure** **5**A), grapiprant treatment not only decreased TGF‐β1 protein level in cell lysate, but also increased TGF‐β1 contents in the supernatant (Figure 5B–D). These findings suggest that EP4 might physically interact with the TGF‐β1 receptor TGFBRII to facilitate the binding of TGF‐β1 to TGFBRII, thus enhancing Smad2/3 activation and profibrotic process. The binding simulations of the TGFBRII extracellular structural domain (PDBid: 1M9Z) with the EP4 transmembrane structural domain (PDBid: 5YWY) was based on Zdock. As shown in Figure 5E and Table S3, Supporting Information, the top ten binding modes of TGFBRII bound to EP4 were analyzed by clustering and region which contained more aggregated models (model 2.3.6.7.9.10). With a significant clustering and the highest scores, model 2 in region was performed as the initial structure for molecular dynamics simulation. During 50ns simulations, the Gln‐253 and Ile‐223 residues of EP4 were found to undergo hydrogen bonding with the Glu‐94 and Ser‐24 residues of TGFBRII, respectively (Figure 5F,G). These interactions serve to reinforce the stability of the EP4‐TGFBRII complex. It is notable that the binding free energy of the complex was −39.64 kcal mol^−1^ (MM/GBSA model). To further investigate the key amino acid residues involved in hydrogen bonding, energy decomposition analysis was conducted. This revealed that the Phe‐12 residue of EP4 had the lowest binding energy contribution (−4.87 kcal mol^−1−1^). The Ile‐223 residue of EP4 formed a hydrogen bond with Ser‐24 residue of TGFBRII (−2.03 kcal mol^−1^), with a minimum binding energy of −3.45 kcal mol^−1^, which exerted stronger binding effect in the binding process. The Arg‐273 residue (−3.13 kcal mol^−1^) and Gln‐253 residue (−2.71 kcal mol^−1^) of EP4 also exhibited a lower binding energy contribution, which played a significant role in complex binding (Figure 5H). The direct interaction between EP4 and TGFBRII receptors was further supported by the Co‐immunoprecipitation (Co‐IP) assay (Figure 5I). In addition, grapiprant suppressed the formation of the EP4‐TGF‐β1/TGFBRII/TGFBRI complex and the binding of TGF‐β1 to TGFBRII (Figure 5J; Figure S17, Supporting Information). Furthermore, adenovirus‐mediated EP4 overexpression markedly enhanced TGF‐β1‐elicited Smad2 and Smad3 phosphorylation in the CFs (Figure 5K,L). In addition, we also examined the expression of TGFBRII in the heart tissues of the EP4^f/f^‐S100A4^Cre+^ mice and in the NRCFs treated with the EP4 antagonist grapiprant. The results demonstrated that EP4 deletion or inhibition does not influence the expression of TGFBRII in the CFs (Figure S18A–D, Supporting Information). Taken together, these data provide evidence that EP4 promotes the TGF‐β1‐Smad2/3 signaling by directly binding to TGFBRII to facilitate the fibrotic process in the CFs.

### 2.7 Blockade of EP4 Attenuates the CF Proliferation in ISO‐Treated Mice and TGF‐β1‐Treated Cells

**Figure 6:**
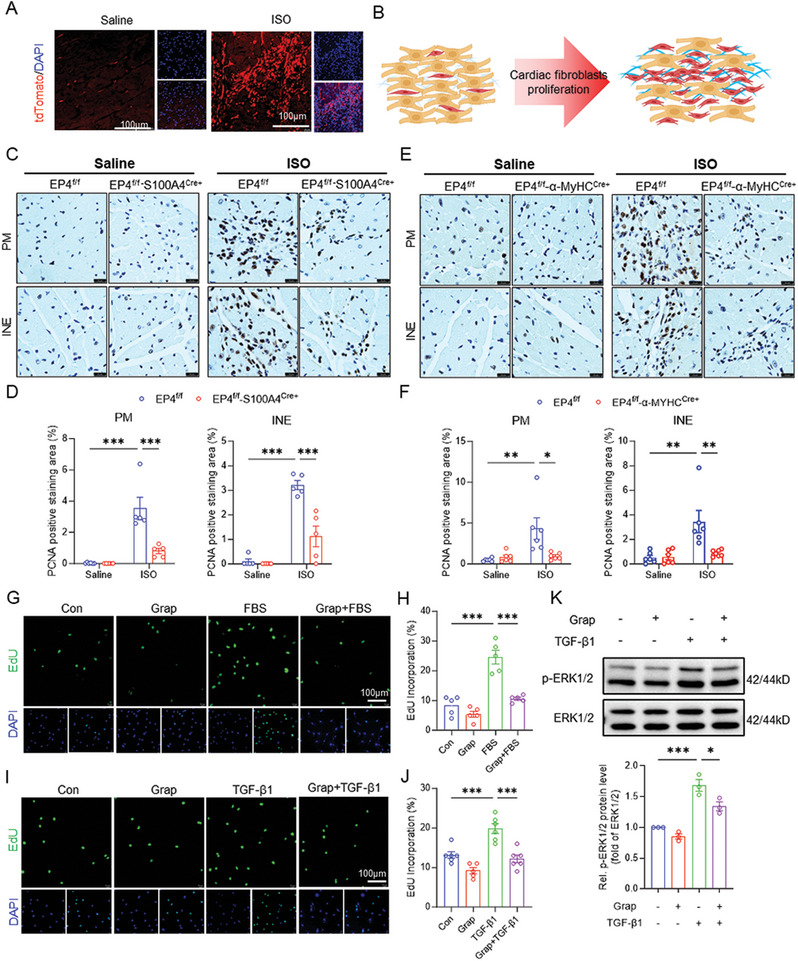
Inactivation of EP4 limits CFs proliferation in vivo and in vitro. A,B) Immunofluorescence analysis of the tdTomato‐positive CFs in the hearts of the tdTomato^f/+^‐S100A4^Cre+^ mice receiving ISO treatment for 7days. Heart sections were stained with DAPI to visualize the nuclei. Scale bar = 100 µm (A). Schematic presentation of ISO‐induced CF proliferation (B). C,D) Representative images of immunohistochemical staining of PCNA in the hearts of the 8–10 weeks old male EP4^f/f^ and EP4^f/f^‐S100A4^Cre+^ mice treated with saline (left panel) or ISO (right panel) for 7 days. PM, papillary muscle area; INE, interstitial area near the endocardium (C). Quantitative analysis of the PCNA‐positive areas by image J software (D). Scale bar = 20 µm. *n* = 5–6 per group. E,F) Representative images of immunohistochemical staining of PCNA in the hearts of the 8–10 weeks old male EP4^f/f^ and EP4^f/f^‐α‐MyHC^Cre+^ mice receiving saline (left panel) or ISO (right panel) treatment for 7 days. PM, papillary muscle area; INE, interstitial area near the endocardium (E). Quantitative analysis of the PCNA‐positive areas by image J software (F). Scale bar = 20µm. *n* = 6 per group. G,H) EdU incorporation analysis showing the effect of grapiprant (1µM) on CF proliferation triggered by FBS (10%) (G). The percentage of EdU‐positive cells (green) relative to DAPI‐stained cells (blue) was calculated (H). Scale bar = 100µm. *n* = 5. I,J) EdU incorporation analysis showing the effect of grapiprant (1µM) on CF proliferation triggered by TGF‐β1 (10 ng mL^−1^) (I). The percentage of EdU‐positive cells (green) relative to DAPI‐stained cells (blue) was calculated (J). Scale bar = 100µm. *n* = 5. K) Western blot analysis of the phosphorylated ERK1/2 and total ERK1/2 in primary cultured neonatal rat cardiac fibroblasts (NRCFs) pretreated with grapiprant (1µM) for 30 min, followed by TGF‐β1 (10 ng mL^−1^) treatment for 30min. Quantitative analysis was performed using image J software. *n* = 3. Data were presented as mean±SEM. *p < 0.05, **p < 0.01, ***p < 0.001 by two‐way ANOVA followed by the Tukey's multiple comparisons test (D,F) or by one‐way ANOVA followed by the Tukey's multiple comparisons test (H,J,K).

By using the Rosa26‐tdTomato lineage tracing mouse, we measured the change in CF cell numbers after ISO treatment. We found that the tdTomato positive cells were substantially increased in the fibrosis area following ISO treatment for 7 days, indicating that the CF population is markedly expanded in response to ISO treatment (**Figure** **6**A,B). We then performed a proliferating cell nuclear antigen (PCNA) immunohistochemical assay to measure the CF proliferation. As expected, PCNA‐positive CFs were dramatically increased in ISO‐treated EP4^f/f^ mouse heart tissue. However, ISO‐induced CF proliferation was remarkably attenuated in both EP4^f/f^‐S100A4^Cre+^ and EP4^f/f^‐α‐MyHC^Cre+^ mice (Figure 6C–F). To further determine the role of EP4 in CF proliferation, we used EdU incorporation assay in cultured NRCFs. The results showed that treatment with both fetal bovine serum (FBS) and TGF‐β1 promoted NRCFs’ proliferation, while pharmacological blockade of EP4 by grapiprant and MF498 both markedly inhibited the proliferation of NRCFs (Figure 6G–J; Figure S19A–D, Supporting Information). Furthermore, we found that pretreatment with both EP4 antagonists inhibited TGF‐β1‐evoked ERK1/2 phosphorylation, which contributes to TGF‐β1‐induced cell proliferation (Figure 6K; Figure S19E, Supporting Information).^[^ ^30^ ^]^ Collectively, these results demonstrate that blocking EP4 in the CFs can suppress TGF‐β1‐induced proliferation both in vivo and in vitro possibly via inhibiting ERK1/2 activation.

### 2.8 Double Inactivation of the CF‐EP4 and CM‐EP4 Improves ISO‐Induced Cardiac Diastolic Dysfunction and Fibrosis

**Figure 7:**
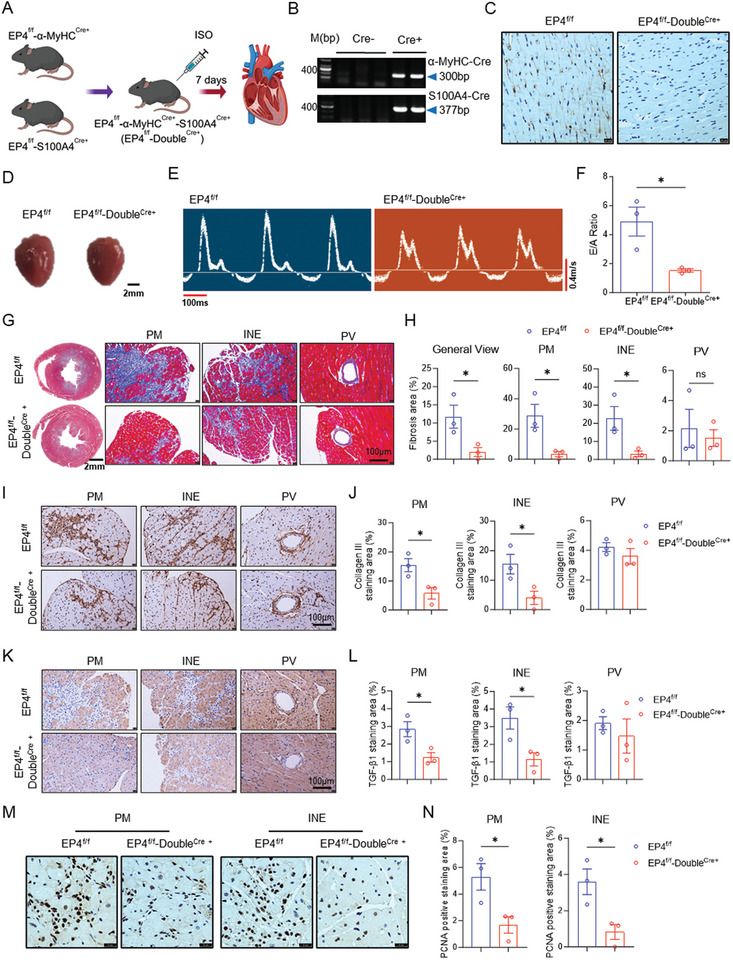
Double knockout of the EP4 gene in both CMs and CFs improves ISO‐induced cardiac diastolic dysfunction and fibrosis. A) The experimental protocol for the generation of the double knockout mice with EP4 deficiency in both CMs and CFs (EP4^f/f^‐Double^Cre+^) mice and ISO‐induced cardiac fibrosis model. The EP4^f/f^‐Double^Cre+^ mice were generated by crossing the EP4^f/f^‐α‐MyHC^Cre+^ mice and EP4^f/f^‐ S100A4^Cre+^ mice. The littermate mice without α‐MyHC‐Cre or S100A4‐Cre (EP4^f/f^) were used as control mice. Male mice aged 8–10 weeks were subcutaneously injected ISO for 7 days to induce cardiac fibrosis. B) PCR‐based genotyping showing the S100A4‐Cre positive and α‐MyHC‐Cre positive bands. C) Representative immunohistochemical staining showing the absence of the EP4 protein in the hearts of the EP4^f/f^‐Double^Cre+^ mice. Scale bar = 20µm. D) Representative photographs showing the hearts of the EP4^f/f^ and EP4^f/f^‐Double^Cre+^ mice receiving ISO treatment for 7 days. Scale bar = 2mm. E,F) Representative echocardiography of peak velocity flow in early diastole (E, m/s) to peak velocity flow in late diastole by atrial contraction (A, m/s) in the EP4^f/f^ mice and EP4^f/f^‐Double^Cre+^ mice injected saline or ISO for 7 days (E). The ratio of E/A was calculated (F). Transverse scale bar = 100ms. Vertical scale bar = 0.4 m s^−1^. *n* = 3 mice per group. G,H) Representative images of Masson's trichrome staining of the hearts of the EP4^f/f^ and EP4^f/f^‐Double^Cre+^ mice receiving ISO injection for 7 days. General view, papillary muscle (PM) area, interstitial area near the endocardium (INE), and perivascular (PV) area were shown, respectively (G). Quantitative analysis of fibrotic areas (blue) was performed by image J software. General view scale bar = 2 mm. Enlarged picture scale bar = 100µm. *n* = 3 per group. I,J) Representative immunohistochemical staining of collagen III in the hearts of the EP4^f/f^ and EP4^f/f^‐Double^Cre+^ mice after ISO injection for 7 days. General view, PM area, INE area, and PV area were shown, respectively (I). Quantitative analysis of collagen III‐positive area was performed by image J software (J). Scale bar = 100µm. *n* = 3 per group. K,L) Representative immunohistochemical staining of TGF‐β1 in the hearts of the EP4^f/f^ and EP4^f/f^‐Double^Cre+^ mice after ISO treatment for 7 days. General view, PM area, INE area, and PV area were shown, respectively (K). Quantitative analysis of TGF‐β1 positive area was performed by image J software (L). Scale bar = 100µm. *n* = 3 per group. M,N) Representative immunohistochemical staining of PCNA in the hearts of the EP4^f/f^ and EP4^f/f^‐Double^Cre+^ mice receiving ISO treatment for 7days. General view, PM area, INE area, and PV area were shown, respectively (M). Quantitative analysis of PCNA‐positive area was performed by image J software (N). Scale bar = 100µm. *n* = 3 per group. Data were presented as mean±SEM. *p < 0.05 by two‐tailed unpaired t test (F,H,J,L,N).

As shown above, deletion of either CF‐EP4 or CM‐EP4 attenuates ISO‐induced cardiac diastolic dysfunction and fibrosis. We further examined whether simultaneous inactivation of both CF‐EP4 and CM‐EP4 has similar beneficial effect. By crossing the EP4^f/f^‐S100A4^Cre+^ mice with the EP4^f/f^‐α‐MyHC^Cre+^ mice, we generated the CF‐EP4 and CM‐EP4 double knockout (EP4^f/f^‐Double^Cre+^) mice (**Figure** **7**A). PCR‐based genotyping and immunohistochemical assays showed that the EP4 gene was deleted in both cardiomyocyte and cardiac fibroblast (Figure 7B,C). The control mice (EP4^f/f^) and EP4^f/f^‐Double^Cre+^ mice were then injected with ISO for 7 days to determine the difference in cardiac function and cardiac fibrosis between two groups of mice. The results showed that the heart weight of the EP4^f/f^‐Double^Cre+^ mice was slightly lighter than that of the EP4^f/f^ mice (Figure 7D; Figure S20A,B, Supporting Information). No significant difference in LV mass, LVAW, and LVPW was observed between EP4^f/f^ and EP4^f/f^‐Double^Cre+^ mice (Figure S20C–H, Supporting Information). Unlike the EP4^f/f^‐S100A4^Cre+^ mice and EP4^f/f^‐α‐MyHC^Cre+^ mice, echocardiographic measurements of cardiac systolic function revealed that the EP4^f/f^‐Double^Cre+^ mice exhibited increased EF and FS, but decreased LVID and LV Vol compared to the EP4^f/f^ mice (Figure S20I–N, Supporting Information). These results suggest that the CF‐EP4 and CM‐EP4 may have synergistic effect on ISO‐induced cardiac damage. Importantly, ISO‐induced cardiac diastolic dysfunction was dramatically ameliorated in the EP4^f/f^‐Double^Cre+^ mice compared to the EP4^f/f^ mice, as reflected by the nearly normal E/A ratio in the EP4^f/f^‐Double^Cre+^ mice (Figure 7E,F). Further investigation using Masson trichrome staining and Sirus red staining revealed that the ECM deposition in the PM and INE areas was significantly decreased in the EP4^f/f^‐Double^Cre+^ mice compared to the EP4^f/f^ mice after ISO treatment (Figure 7G,H; Figure S21A,B, Supporting Information). Similarly, the expression levels of collagen III in the PM and INE areas of the EP4^f/f^‐Double^Cre+^ mice were significantly less than that in the EP4^f/f^ mice (Figure 7I,J). Like the EP4^f/f^‐α‐MyHC^Cre+^ mice, the EP4^f/f^‐Double^Cre+^ mice exhibited a marked reduction in ISO‐induced TGF‐β1 expression in the CMs (Figure 7K,L). Consistent with the findings in both EP4^f/f^‐S100A4^Cre+^ mice and EP4^f/f^‐α‐MyHC^Cre+^ mice, the EP4^f/f^‐Double^Cre+^ mice exhibited a significant decrease in PCNA‐positive cells in the PM and INE areas following ISO treatment (Figure 7M,N). Additionally, the expression levels of β‐AR and TGFBRII were similar between the EP4^f/f^ mice and EP4^f/f^‐Double^Cre+^ mice (Figure S21C–F, Supporting Information). Collectively, these data demonstrate that simultaneous deletion of CF‐EP4 and CM‐EP4 can improve ISO‐induced cardiac diastolic dysfunction and fibrosis.

### 2.9 Pharmacological Blockade of EP4 Improves ISO‐Induced Cardiac Fibrosis

**Figure 8:**
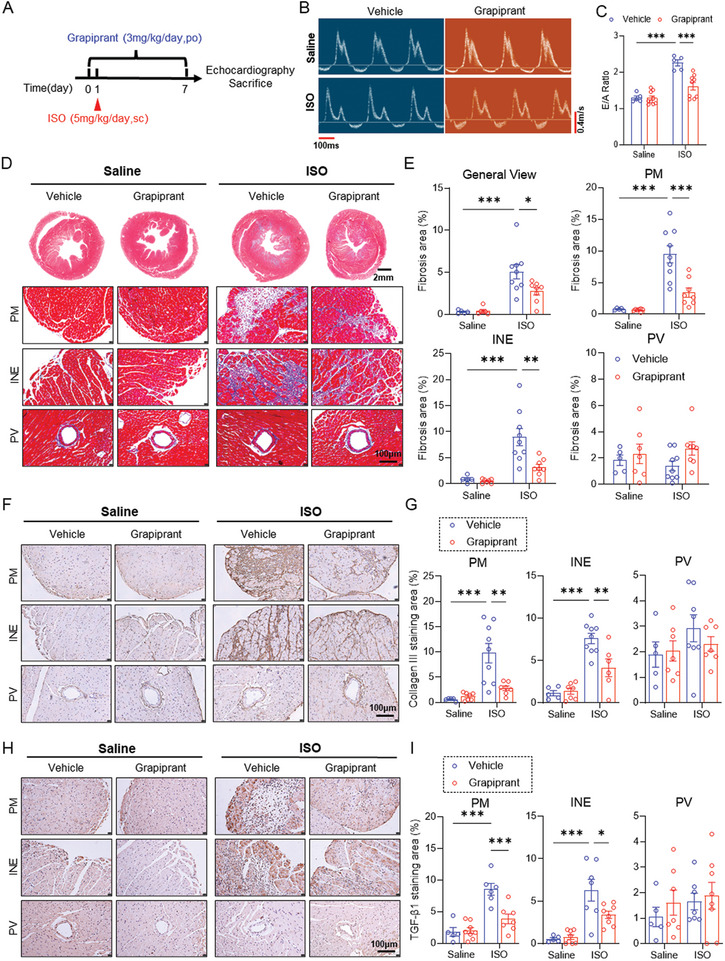
Grapiprant treatment improves ISO‐induced cardiac diastolic dysfunction and cardiac fibrosis in vivo. A) The schematic showing of the experimental protocol for testing the effect of grapiprant on ISO‐induced cardiac fibrosis in mice. The 8–10 weeks old male mice were treated with grapiprant (3 mg kg^−1^ day^−1^, po) or vehicle from day 0 to day 7 and ISO (5 mg kg^−1^ day^−1^) from day 1 to day 7. At the end of the experiment, the mice were performed echocardiography and then sacrificed. B,C) Representative echocardiography showing the effect of grapiprant treatment on peak velocity flow in early diastole (E, m/s) and late diastole by atrial contraction (A, m/s) in mice with cardiac fibrosis (B). Saline: top panel, ISO: bottom panel. The ratio of E/A was calculated (C). Transverse scale bar = 100ms. Vertical scale bar = 0.4 m s^−1^. *n* = 5–9 mice per group. D,E) Masson's trichrome staining of the hearts of ISO‐treated mice receiving grapiprant treatment. General view, PM area, INE area, and PV area were shown, respectively. Saline: left panel, ISO: right panel. (D). Quantitative analysis of fibrotic area (blue) was performed by image J software (E). General view scale bar = 2 mm. Enlarged picture scale bar = 100µm. *n* = 5 to 9 per group. F,G) Representative immunohistochemical staining of collagen III in the hearts of ISO‐treated mice receiving grapiprant treatment. The PM area, INE area, and PV area were shown, respectively. Saline: left panel, ISO: right panel. (F). Quantitative analysis of collagen III‐positive area was performed by image J software (G). Scale bar = 100µm. *n* = 5–9 per group. H,I) Representative immunohistochemical staining of TGF‐β1 in the hearts of ISO‐treated mice receiving grapiprant treatment. The PM area, INE area, and PV area were showing, respectively. Saline: left panel, ISO: right panel. (H). Quantitative analysis of TGF‐β1‐positive area was performed by image J software (I). Scale bar = 100µm. *n* = 5 to 8 per group. Data were presented as mean±SEM. *p < 0.05, **p < 0.01, ***p<0.001 by two‐way ANOVA followed by the Tukey's multiple comparisons test (C, E, G and I).

To test whether pharmacological targeting on EP4 ameliorates ISO‐induced cardiac fibrosis in vivo, we pretreated mice with the EP4 antagonist grapiprant 1 day prior to ISO and continued to treat the mice along with ISO for 7 days (**Figure** **8**A). The results showed that administration of grapiprant attenuated ISO‐induced cardiac hypertrophy (Figure S22A–H, Supporting Information). Echocardiographic measurement revealed that grapiprant treatment significantly improved cardiac diastolic function, with the systolic function unchanged (Figure 8B,C; Figure S22I–N, Supporting Information). Histology analyses using Masson trichrome staining and Sirus red staining showed that ECM accumulation in the PM and INE areas was significantly decreased in grapiprant‐treated mice receiving ISO injection for 7 days (Figure 8D,E; Figure S23A,B, Supporting Information). Consistently, grapiprant treatment significantly reduced ISO‐induced collagen I and collagen III mRNA expression in the heart (Figure S23C,D, Supporting Information) and protein expression in the PM and INE areas (Figure 8F,G; Figure S23E,F, Supporting Information). Additionally, the ISO‐induced upregulation of TGF‐β1 was also reduced by grapiprant treatment at both mRNA and protein level (Figure 8H,I; Figure S23G, Supporting Information). Taken together, these findings provide clear evidence that pharmacological blockade of cardiac EP4 represents a potential approach to treat cardiac fibrosis.

## 3 Discussion

In the present study, we demonstrate that deletion of EP4 in the CMs or CFs ameliorates β‐adrenergic activation‐induced cardiac fibrosis through distinct mechanisms. We found that inactivation of EP4 in the CMs inhibits ISO‐induced TGF‐β1 expression by suppressing the cAMP/PKA pathway, thereby reducing the paracrine action on the CFs. Targeted deletion of EP4 in the CFs attenuates the TGF‐β1 signaling by disrupting the physical interaction between EP4 and TGFBRII, thus inhibiting the proliferation and ECM production of the CFs. Importantly, double knockout of the EP4 gene in both CMs and CFs and pharmacological blockade of EP4 effectively ameliorates ISO‐induced cardiac diastolic dysfunction and cardiac fibrosis. These findings demonstrate that EP4 may represent an attractive target for the treatment of cardiac fibrosis.

Cardiac fibrosis is a common pathological process in various myocardial diseases, characterized by excessive ECM accumulation in the myocardial interstitium.^[^ ^3^, ^31^ ^]^ Consistent with previous reports, the present study confirmed that low dose of ISO‐triggered sustained β‐adrenergic activation induces ECM production mainly in the PM and INE areas rather than in the PV area, which leads to myocardial stiffness and cardiac diastolic dysfunction.^[^ ^32^ ^]^ Knocking out EP4 gene in the CFs and CMs or both, and administration of the EP4 antagonist markedly reduce ISO‐induced ECM deposition and cardiac diastolic dysfunction in vivo. These results indicate that EP4 in both cardiomyocyte and cardiac fibroblast play an important role in the pathogenesis of cardiac fibrosis. Targeting either cardiomyocyte or cardiac fibroblast EP4 may help improve cardiac fibrosis and related heart dysfunction.

It is well documented that sustained β‐adrenergic activation by ISO leads to increased production of an array of cytokines, including TGF‐β1 and other pro‐inflammatory factors.^[^ ^33^, ^34^, ^35^ ^]^ In the present study, we found that TGF‐β1 is prominently expressed in the CMs and markedly upregulated by ISO treatment. Deletion of the CM‐EP4 rather than the CF‐EP4 suppressed ISO‐induced upregulation of TGF‐β1 expression in vivo. In vitro studies further revealed that ISO induced TGF‐β1 expression in primary cultured CMs, but not in primary cultured CFs. In addition, ISO treatment had no effect on collagens synthesis in cultured CFs. However, we found that the supernatant of the CMs treated with ISO dramatically induced collagen production in the CFs, which was blocked by SB431542, a TGF‐β receptor kinase inhibitor.^[^ ^36^ ^]^ These in vivo and in vitro findings demonstrate that in response to ISO treatment, the CMs are the primary source of TGF‐β1, which triggers the TGF‐β‐Smad pathway to promote fibrotic process in the CFs via a paracrine mechanism.

During cardiac fibrosis, proliferated CFs act as an important contributor.^[^ ^37^, ^38^ ^]^ It has been previously reported that cardiac fibrosis in mice with hypertrophic cardiomyopathy is mediated by non‐myocyte proliferation and requires TGF‐β.^[^ ^6^ ^]^ Indeed, there is an abundant CFs in the myocardial interstitial area after ISO treatment, accompanied by a significant upregulation of TGF‐β1 in the CMs. In the present study, we provided evidence that specific deletion of either CM‐EP4 or CF‐EP4 decreased the PCNA‐positive CFs in the myocardial interstitium possibly via decreasing TGF‐β1 production in the CMs or blocking TGF‐β1 signaling in the CFs, respectively. Consistently, in vitro experiments also showed that TGF‐β1 treatment significantly enhanced proliferation of primary cultured CFs, which was markedly blocked by pretreatment with the EP4 antagonists. Since phosphorylation of ERK1/2 is associated with TGF‐β1‐induced cardiac fibroblast proliferation,^[^ ^30^ ^]^ and EP4 also exerts proliferative effect through the ERK1/2 pathway,^[^ ^39^ ^]^ these results suggest that blockade of EP4 may abolish TGF‐β1‐induced proliferation in the CFs via suppressing the ERK1/2 signaling. This is consistent with the observations that EP4 antagonist E7046 significantly inhibited M2 macrophage‐mediated hepatic stellate cells autophagy and improved liver fibrosis in NAFLD mice via suppressing ERK1/2 pathway.^[^ ^40^ ^]^

Unlike the anti‐fibrotic action of EP4 reported in transverse aortic constriction (TAC)‐induced and myocardial infarction‐associated cardiac fibrosis,^[^ ^24^, ^41^ ^]^ we found EP4 exerts pro‐fibrotic effect in ISO‐induced cardiac fibrosis model. The reason for this discrepancy is not clear and may be due to the difference in underlying mechanisms of various disease models. By targeting on the β‐AR, ISO appears to activate the cAMP‐PKA pathway to exert its biological role in the heart. As previously reported, ISO induced TGF‐β1 expression mainly through the cAMP‐PKA‐CREB signaling pathway.^[^ ^28^ ^]^ In our study, we found that H89, a selective PKA inhibitor, completely blocked ISO‐mediated increase in TGF‐β1 in primary cultured CMs. Similarly, the inhibitory effect of the EP4 antagonists on ISO‐induced TGF‐β1 upregulation was significantly diminished in the presence of H89. Because the cAMP‐PKA pathway is the major mechanism mediating the biological effect of EP4, these findings suggest a crosstalk between ISO‐ and EP4‐elicited signaling pathways. Blockade of EP4 is capable of diminishing ISO‐induced activation of the cAMP‐PKA pathway, thereby attenuating ISO‐induced TGF‐β1 expression in the CMs. CREB is an important downstream target of the PKA signaling pathway, and it has been reported to function as a transcription factor for TGF‐β1.^[^ ^28^, ^29^ ^]^ Consistantly, our findings showed that blockade of EP4 suppressed CREB activation, suggesting that inactivation of EP4 suppresses the cAMP/PKA/CREB pathway, thereby reducing TGF‐β1 expression. However, other transcription factors, such as NF‐κB (nuclear factor kappa B), SP1 (Specificity Protein 1), and EGR1 (early growth response 1), may also play a role in EP4‐regulated TGF‐β1 expression.^[^ ^42^, ^43^, ^44^ ^]^ Further studies are required to explore this aspect in greater detail.

Similar to the findings observed in the CMs, the EP4 antagonists also efficiently attenuated TGF‐β1‐induced Smad2/3 phosphorylation and ECM production in the CFs. To test whether the cAMP‐PKA pathway affects the TGF‐β1‐induced fibrotic process in the CFs, we treated primary cultured CFs with the PKA inhibitor H89 and the activator forskolin in the presence of TGF‐β1. We found that neither inhibition of PKA by H89 nor activation of PKA by forskolin altered TGF‐β1‐elicited Smad2/3 phosphorylation, suggesting that EP4 promotes TGF‐β1‐induced Smad2/3 phosphorylation by activating its upstream tyrosine kinase receptor TGFBRII, rather than through the cAMP‐PKA pathway.^[^ ^45^ ^]^ Indeed, the autodocking analysis and Co‐IP assay revealed a direct physical interaction between EP4 and TGFBRII, which was attenuated by the EP4 antagonist. Based on these findings, we speculate that EP4 may act as a scaffold for TGFBRII, thereby promoting the interaction with TGFBRI to enhance the downstream Smad2/3 signaling.^[^ ^46^ ^]^ Blockade of EP4, therefore, can limit TGF‐β1‐elicited pro‐fibrotic process via interrupting the formation of the EP4/TGFBRII complex in the CFs.

In addition to the TGF‐β/Smad signaling pathway, other classical fibrosis‐related pathways may also play significant roles in the process of cardiac fibrosis. For instance, the Wnt/β‐catenin signaling pathway has been implicated in cardiac fibrosis through the promotion of myofibroblast activation and ECM deposition.^[^ ^47^ ^]^ The renin‐angiotensin‐aldosterone system (RAAS) is another well‐established pathway contributing to fibrosis by activating pro‐fibrotic mediators such as angiotensin II and aldosterone, which can enhance ECM production and fibroblast proliferation.^[^ ^48^ ^]^ Furthermore, the inflammatory NF‐κB pathway is known to mediate fibrosis by regulating the expression of pro‐inflammatory cytokines and chemokines, which can further amplify fibro‐inflammatory responses.^[^ ^49^ ^]^ Given the multifaceted roles of EP4 in cardiovascular diseases, it is plausible that EP4 may influence cardiac fibrosis not only through the TGF‐β/Smad pathway but also by interacting with these additional signaling pathways.^[^ ^50^, ^51^, ^52^ ^]^ Exploring these potential interactions could provide deeper insights into the complex mechanisms of EP4‐mediated fibrogenesis and offer new perspectives for therapeutic strategies.

Besides, it has been known that the interaction between the CMs and CFs is a crucial process for maintaining cardiac homeostasis and facilitating remodeling.^[^ ^53^ ^]^ In our study, we found that the CM‐derived TGF‐β1 exerts a paracrine effect by promoting CF proliferation and activation through targeting the TGF‐β1 receptor on the CFs. Additionally, we observed that ISO treatment stimulated PGE_2_ production in the CMs, while it had no significant effect on PGE_2_ levels in the CFs. This finding suggests that the interaction between the CMs and CFs is not solely dependent on TGF‐β1 but also mediated by other small molecules, such as PGE_2_. On the other hand, the CFs not only act as recipient cells, responding to the paracrine signals from the CMs, but they may also play an active role in influencing CM function and survival. In support, a recent study reported that the CFs are able to modify the cardiac microenvironment and release extracellular substances.^[^ ^54^ ^]^

Our data demonstrate that deletion of EP4 effectively reduces cardiac fibrosis in both male and female mice following ISO treatment. This suggests that the protective effect of EP4 knockout on fibrotic remodeling is not sex‐dependent. While sex differences are known to influence various aspects of cardiac physiology,^[^ ^55^ ^]^ our findings highlight a conserved role of EP4 signaling in regulating fibrosis across sexes. However, which sex has more profound beneficial effect warrants further investigation.

Due to an important role of EP4 in the pathogenesis of many human and animal diseases, multiple specific EP4 antagonists have been developed.^[^ ^56^, ^57^ ^]^ Among them, grapiprant is an orally administered EP4 antagonist that has now been proven to have excellent analgesic effects on pets with good safety profile.^[^ ^58^, ^59^, ^60^ ^]^ In the present study, the mice receiving grapiprant treatment prior to or after ISO injection exhibited improved cardiac diastolic function and fibrosis, suggesting grapiprant is likely used for the prevention and treatment of cardiac fibrosis. Besides, grapiprant has also been reported to have anti‐inflammatory effects.^[^ ^60^, ^61^ ^]^ Given the fact that ISO treatment facilitates the release of pro‐inflammatory factors in the heart,^[^ ^62^, ^63^, ^64^ ^]^ it would be important to determine whether grapiprant also exerts its anti‐inflammatory function in ISO‐induced cardiac fibrosis.

In the present study, we utilized the S100A4‐Cre mice to induce cardiac fibroblast EP4 gene deletion, since S100A4‐Cre has been previously found to mediate a robust gene deletion specifically in the CFs.^[^ ^65^ ^]^ We are aware of the possible mild off‐target leakage of this mouse line in a few other cell types such as endothelial cells and macrophages, both of which might contribute to cardiac fibrosis by releasing cytokines and undergoing endothelial‐mesenchymal transition (EndMT).^[^ ^66^, ^67^, ^68^ ^]^ In our study, we observed a large number of tdTomato‐positive cells present in the interstitial areas rather than in the endothelium in the hearts of the S100A4^Cre+^ Roas26‐tdTomato lineage tracing mice, suggesting the ECs might not be the major cell type involved in ISO‐elicited cardiac fibrosis. As previously reported, the inflammation is critical for cardiac remodeling^[^ ^69^ ^]^ and EP4 plays an important roles in the macrophages.^[^ ^70^ ^]^ Therefore, it would be important to elucidate the role of the macrophage EP4 in the pathogenesis of cardiac fibrosis in the future.

In summary, the present study reports an undesirable role of EP4 in ISO‐induced cardiac fibrosis, where EP4 expression is markedly increased. Specific deletion of EP4 in the CMs, CFs or both and pharmacological blockade of EP4 effectively improves cardiac diastolic dysfunction and heart fibrosis. Therefore, EP4 may represent an attractive target for the treatment of cardiac fibrosis and related heart dysfunction.

## 4 Experimental Section

### Animal

Cardiomyocyte‐specific EP4 gene knockout mice (EP4^f/f^‐α‐MyHC^Cre+^) were obtained by crossing the EP4^f/f^ mice with the α‐MyHC^Cre+^ mice (JAX stock #011038) on C57BL/6 background. The EP4^f/f^ mice were also crossed with the S100A4^Cre+^ mice (JAX stock #030644) to obtain cardiac fibroblast‐specific EP4 gene knockout mice (EP4^f/f^‐S100A4^Cre+^)^[^ ^65^ ^]^. The EP4^f/f^‐α‐MyHC^Cre+^ mice were crossed with EP4^f/f^‐S100A4^Cre+^ mice to generate cardiomyocyte (CM)‐ and cardiac fibroblast (CF)‐EP4 gene double knockout mice (EP4^f/f^‐Double^Cre+^). The Rosa26‐tdTomato lineage tracing mice were kindly gifted by Prof. Qingbo Xu (Zhejiang University) and crossed with the S100A4^Cre+^ mice to obtain CF‐specific tracing mice. All conditional knockout mice were verified by genotyping and histology staining. Wild‐type (WT) C57BL/6 mice and neonatal Sprague‐Dawley (SD) rats were obtained from the specific pathogen‐free (SPF) Animal Experiment Center of Dalian Medical University. The mice were bred with the standard diet in a SPF environment with standard temperature, humidity, and 12/12 h light‐dark cycle conditions. All animal care and experimental procedures were reviewed and approved by the Animal Care and Use Review Committee of East China Normal University (m+R20241107). The study conformed to the Guide for the Care and Use of Laboratory Animals published by the US National Institutes of Health.

### Chemicals and Reagents

Primary antibodies against EP4 (sc‐55596) were purchased from Santa Cruz Biotechnology. Smad2 (D43B4) (Cat# 5339S), p‐Smad2 (S465/4667, E8F3R) (Cat# 18338S), Smad3 (C67H9, Cat# 9523S), p‐Smad3 (S423/425, C25A9) (Cat# 9520S), p‐PKA Substrate (Cat# 9621L) antibodies were obtained from Cell Signaling Technology. TGFBRI (Cat# ab235578) antibody was obtained from Abcam. EP4 (Cat# 24895‐1‐AP), TGFBRII (Cat# 66636‐1‐Ig), TGF‐β1 (Cat# 21898‐1‐AP), collagen I (Cat# 14655‐1‐AP), collagen III (Cat# 22734‐1‐AP), β‐AR (Cat# 28323‐1‐AP) antibodies were obtained from Proteintech. Recombinant Human TGF‐β1 (Cat# 100–21) was purchased from Peprotech. H89 (Cat# HY‐15979), PD98059 (Cat# HY‐12028), Adezmapimod (SB 203580, Cat# HY‐10256), SP600125 (Cat# HY‐12041) were purchased from MCE.

### ISO‐Induced Cardiac Fibrosis

The cardiac fibrosis model was established by subcutaneous injection of ISO (Sigma, I5627) as described previously^[^ ^9^ ^]^. In brief, ISO was dissolved in saline (1mg mL^−1^) and 8–10 weeks old mice were subcutaneously injected with ISO (5 mg kg^−1^ day^−1^) for 7 days. Saline (5µL g^−1^) injected mice were served as control groups. For the pharmacological experiment, wild‐type mice were intragastrically administrated with grapiprant (3mg kg^−1^ day^−1^, 1 mg mL^−1^ in corn oil) one day before ISO injection and throughout the ISO treatment. The mice were subjected to echocardiography analysis 7 days after ISO or saline injection. The mice were then sacrificed using overdose of CO_2_ and the hearts were removed and prepared for appropriate analyses. Body weight and tibial length were also recorded for further analysis.

### Echocardiography

Cardiac function was measured using a high‐resolution echography system (Vevo 3100, VisualSonics Inc., Toronto, Canada) in the isoflurane‐anesthetized mice (1–2% isoflurane). For cardiac diastolic function, the apical four‐chamber view was acquired and the peak velocity flow in early diastole and peak velocity flow in late diastole by atrial contraction were measured under Doppler color mode. For cardiac systolic function, parasternal short‐axis images were acquired in B‐mode and in this view, the M‐mode cursor was positioned perpendicular to the maximum LV dimension in end‐diastole and systole, and M‐mode images were acquired. The pictures were used to analyze the E/A ratio, left ventricular (LV) mass, LV wall thickness, LV internal diameter, LV volume, ejection fraction (EF) and fractional shortening (FS) using the Vevo 3100 software (version 1.5.0).

### Histology and Immunostaining for Tissue Sections

The heart tissues were fixed in 4% paraformaldehyde (PFA) for 24 h, then embedded in paraffin and sectioned at 4‐µm intervals. H&E staining, Masson trichrome staining and Sirius red staining were performed according to standard procedures. For immunohistochemistry staining (IHC), paraffin sections were performed dewaxing and rehydration, followed by antigen retrieval and pretreatment of 3% H_2_O_2_. Then, the sections were blocked with bovine serum albumin (BSA) (5%, BioFroxx) for 1 h and incubated with the primary antibodies for EP4 (1:50, Santa Cruz), collagen I (1:250, Proteintech), collagen III (1:250 Proteintech), TGF‐β1 (1:500, Proteintech), or PCNA (1:200, Abcam), respectively at 4 °C overnight. Then, the sections were incubated with appropriate horseradish peroxidase (HRP)‐conjugated secondary antibodies (ZSGB‐BIO) for 1 h at room temperature and visualized with 3,3′‐diaminobenzidine (DAB) staining kit (ZSGB‐BIO) and counterstained with hematoxylin. For immunofluorescence staining (IFC), the hearts were embedded in optimal cutting temperature (OCT) compound (Sakura Fine technical) and stored at −80 °C. OCT‐embedded tissues were cut in 5‐µm serial cross sections. The slices were incubated with the primary antibody against EP4 (1:50, Santa Cruz) and followed by incubation with fluorescence‐conjugated secondary antibody. After immunofluorescence staining, the sections were stained with 4,6‐diamidino‐2‐phenylindole (DAPI) to visualize the nuclei. The images were captured with a microscope (DM4B, Leica, Germany) or confocal microscope (SP8, Leica, Germany).

### Cell Culture

Primary cardiomyocytes and cardiac fibroblasts were isolated from neonatal SD rats as previously described.^[^ ^71^ ^]^ Briefly, 1 to 2‐day old neonatal SD rats were euthanized by swift decapitation with sharp surgical scissors, sterilized with 75% ethanol for 30 s and the hearts were removed and placed in cold phosphate buffer saline (PBS). Then, the hearts were cut into a size of 1mm^3^ and digested with a digestive solution containing trypsin (0.04%, Gibco) and type I collagenase (0.1%, Sigma). After digestion, the cells were cultured on 100‐mm dishes (BIOFIL) in DMEM supplemented with 10% FBS plus 1% penicillin and streptomycin for 2 h. Then, the cardiac fibroblasts were attached to the bottoms and unattached cardiomyocytes were removed to fresh dishes. Cardiomyocytes at passage 0 (P0) and cardiac fibroblasts at P1‐2 were used for the experiments. Both cardiac myocyte and fibroblast cultures were changed to serum‐free DMEM for 12 h prior to further treatment.

### Isolation of Adult Mouse Cardiomyocytes

Adult mouse cardiomyocytes were isolated from the EP4^f/f^ and EP4^f/f^‐α‐MyHC^Cre+^ mice (8–10 weeks old) following the method described in the literature.^[^ ^72^ ^]^ The freshly isolated cardiomyocytes were stimulated with isoproterenol (20µM) for 0.5 h, and the protein was extracted for subsequent Western blot analysis.

### Western Blot

The cells were lysed in a RIPA buffer containing the protease inhibitor cocktail (MedChemExpress). After centrifugation at 12,000 rpm and 4 °C for 15 min, the protein concentration was quantified by a BCA Protein Assay Kit (Thermo Fisher Scientific). The cell lysates were mixed with 6X SDS‐PAGE loading buffer, fractionated with 10% SDS‐PAGE, and transferred onto nitrocellulose filter membranes. The membranes were then incubated with the primary antibodies over night at 4 °C, followed by incubation with secondary antibody for 1 h at room temperature. Finally, the membranes were incubated with the SuperLumia ECL Plus HRP Substrate Reagent and signals from immunoreactive bands were visualized using a Chemiluminescent Imaging System (Tanon 5200, China).

### Quantitative RT‐PCR

Total mRNA was extracted by Trizol reagent, quantified by DanoDrop 2000, and reverse‐transcribed to cDNA. Primers were designed from the known sequences of mouse genes or rat genes listed in Table S1, Supporting Information. 18S was used as an internal standard. SYBR Green (Invitrogen) was used as fluorochrome according to the manufacturer's instructions. The PCR were performed on the ABI 7300 plus system using the reactions of 94 °C for 5 min, and then 35 cycles of 94 °C for 30 s, 59 °C for 30 s, and 72 °C for 30 s, followed by extension at 72 °C for 5 min.

### Cell Immunofluorescence

For cell immunofluorescence staining, the cells were fixed with 4% PFA at room temperature for 15 min and permeabilized with 0.1% Triton X‐100/PBS solution at room temperature for 10 min. Then, the cells were blocked with 5% BSA for 1 h and incubated with the primary antibody against Smad3 (1:250) at 4 °C overnight. Followed by incubation with an Alexa Fluor 488‐conjugated secondary antibody (1:500, Thermos Fisher Scientific), cells were counterstained with DAPI and mounted with glycerol before imaging with confocal microscopy (SP8, Leica, Germany).

### TGF‐β1 Measurement

The cell medium was harvested and immediately frozen in −80 °C. The level of TGF‐β1 in the medium was measured using the ELISA kit (Boster Bioengineering Co., Ltd.).

### PGE_2_ Measurement

The cell supernatant was rapidly collected and centrifuged at 3000 rpm for 10 min at 4 °C to remove particulate matter and polymers. The concentration of PGE_2_ in the medium was measured using the ELISA kit (Jiangsu Meimian Industrial Co., Ltd).

### EdU Incorporation Assay

Cardiac fibroblast proliferation was assessed by 5‐ethynyl‐2′‐deoxyuridine (EdU) incorporation assay (Beyotime) according to the manufacturer's instructions. In brief, cells were incubated with EdU at a final concentration of 10 µM for 2 h, fixed with 4% paraformaldehyde for 15 min, and then treated with 0.3% Triton X‐100 for 15 min. The cellular nuclei were stained with DAPI. EdU‐positive cells were observed under a microscope (DM4B, Leica, Germany). EdU incorporation was finally expressed to evaluate the proportion of proliferating CFs.

### Co‐Immunoprecipitation

Cardiac fibroblasts were washed with pre‐cooled PBS and lysed using co‐IP lysis buffer (PMSF, 1mM), supplemented with protease inhibitors. The suspension was centrifuged for 15 min at 4 °C by 12000 rpm, the supernatants were collected and kept on ice. Protein A/G beads (BEAVER) was incubated with mouse anti‐TGFBRII (5 µg mL^−1^) for 15 min at room temperature. The beads‐antibody complex was incubated with the CFs supernatants overnight at 4 °C. The bound proteins were eluted by boiling with loading buffer and were analyzed by western blot with anti‐EP4 (1:500), anti‐TGFBRII (1:5000), anti‐TGFBRI (1:1000) or anti‐TGF‐β1 (1:1000) antibody, respectively.

### Protein–Protein Interaction Prediction

For TGFBRII (UNPID: P37173), crystal structure of human TGF‐beta type II receptor extracellular domain was obtained from the PDB database (https://www.rcsb.org/) (PDB code: 1M9Z). The crystal structure of Prostaglandin E receptor EP4 (UNPID: P35408) transmembrane domain was also obtained (PDB code: 5YWY). The relaxed conformation of structures from 20 ns MD simulations were used for predicting the protein–protein interaction. The protein‐protein docking process was conducted with Zdock 3.0.2^[^ ^73^ ^]^ and HawkDock.^[^ ^74^ ^]^ For each system, the complex structure with the top‐rank Zdock score in the largest cluster was selected, then re‐rank top10 models by MM/GBSA to do the further MD simulations and structural analysis.

### Molecular Dynamics Simulations

The MD simulations were performed by Gromacs 2021.1 for the whole optimization. The solvated complex of model2 in a cubic TIP3P water box with 1nm distance from the edge was used as the initial structures for 50 ns MD simulations employing the amber99sb force field. After two steps of energy minimization, the temperature of the system was gradually heated to 300K over 100ps to perform the 2ns NVT equilibration. Finally, the 50ns MD simulations were carried out with the LINCS algorithm to restrain the hydrogen positions at their equilibrium distances. Structural analysis was evaluated by MM‐GBSA calculation between 10ns to 50ns simulations using HawkDock server. All molecular graphics and the putative contacts such as hydrophobic interaction and hydrogen bonding between TGFBRII and EP4 were monitored over the course of the entire simulations by the PyMOL educational version and the 2D interaction profiles were generated using LigPlot.^+[^ ^75^ ^]^

### Graphics Drawing

The experimental scheme and graphical abstract were created with MedPeer (medpeer.cn).

### Statistical Analysis

All data are presented as mean ± SEM. Statistical analyses were performed using Graph Pad Prism 10 software. Comparisons between 2 groups were tested by 2‐tailed Student's *t*‐test. Comparisons among multiple groups were made by ANOVA. *p* < 0.05 was considered statistically significant.

## Conflict of Interest

The authors declare no conflict of interest.

## Author Contributions

H.X.: Conceptualization, Methodology, Formal analysis, Writing – Original Draft. X.M.: Validation, Investigation. Y.W.: Methodology, Formal analysis. C.Z., B.L., Y.Z., M.Z., L.Y., M.H., and Y.W.: Investigation. H.L. and Y.Y.: Autodocking. Y.Q.: Resources. L.C., Y.G., and X.Z.: Supervision, Writing – Review & Editing, Project administration. H.X. and X.M. had equal intellectual contribution and their order as co–first authors were determined by the amount of time they contributed. All authors revised the manuscript and gave final approval for publication.

## Supporting information



Supporting Information

## Data Availability

The data that support the findings of this study are available in the supplementary material of this article.
