# Supplementary material for: Targeting the E Prostanoid Receptor EP4 Mitigates Cardiac Fibrosis Induced by β‐Adrenergic Activation
Source: Adv Sci (Weinh). 2025 Feb 7;12(12):2413324. doi: 10.1002/advs.202413324 (PMC11948031; doi:10.1002/advs.202413324)
Supplement: Supplementary file 1 — Supporting Information [file ADVS-12-2413324-s001.pdf]

ADVANCED SCIENCE
Open Access

Supporting Information

for *Adv. Sci.*, DOI 10.1002/advs.202413324

Targeting the E Prostanoid Receptor EP4 Mitigates Cardiac Fibrosis Induced by β-Adrenergic Activation

Hu Xu, Xiuhui Mao, Yali Wang, Chunhua Zhu, Bo Liang, Yihang Zhao, Mengfei Zhou, Lan Ye, Mengting Hong, Huishu Shao, Yashuo Wang, Haonan Li, Yinghui Qi, Yongliang Yang, Lihong Chen, Youfei Guan and Xiaoyan Zhang**

Supporting Information

Targeting the E Prostanoid Receptor EP4 Mitigates Cardiac Fibrosis Induced by β-Adrenergic Activation

Hu Xu, Xiuhui Mao, Yali Wang, Chunhua Zhu, Bo Liang, Yihang Zhao, Mengfei Zhou, Lan Ye, Mengting Hong, Huishu Shao, Yashuo Wang, Haonan Li, Yinghui Qi, Yongliang Yang, Lihong Chen, Youfei Guan, Xiaoyan Zhang*.*

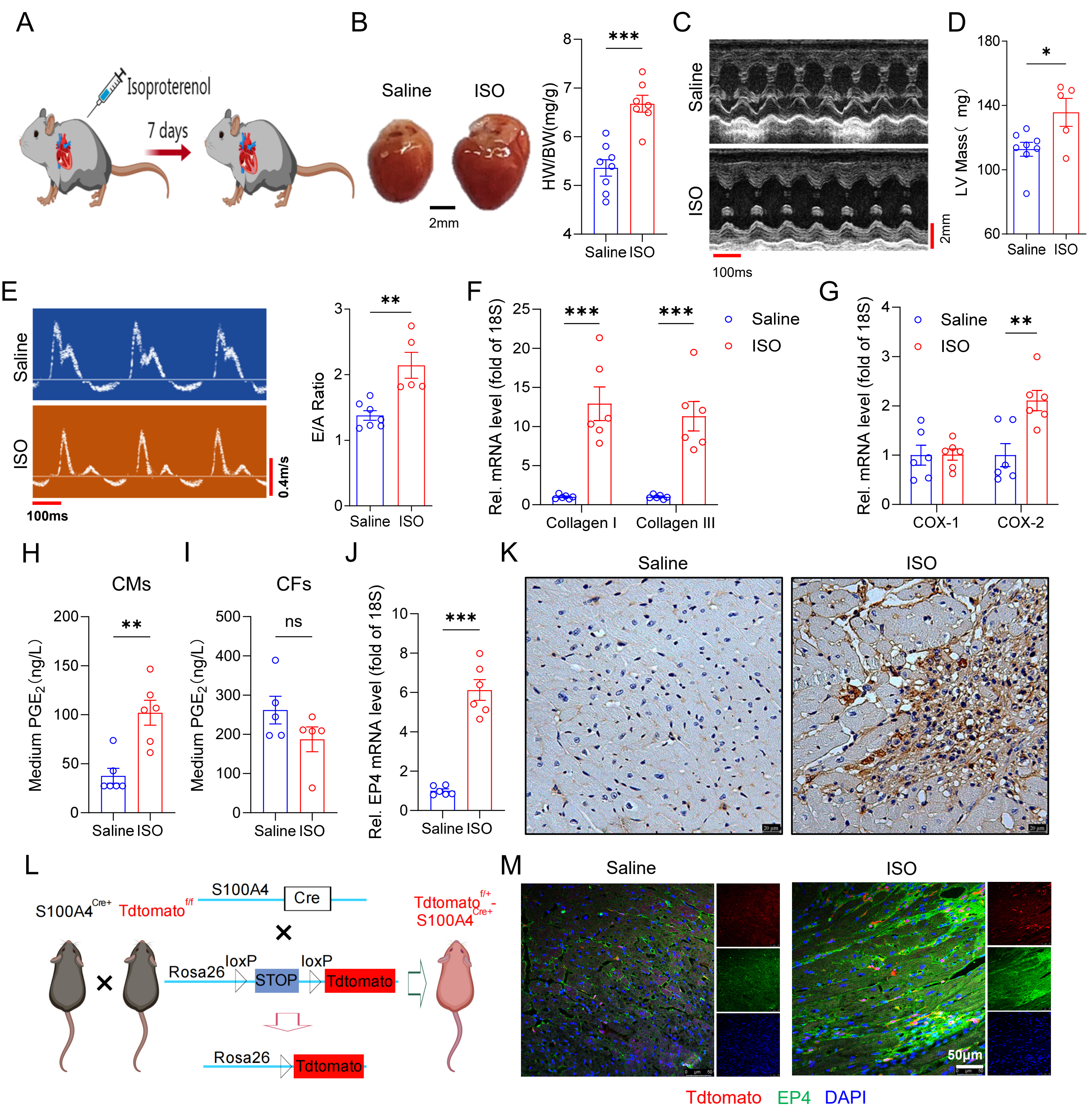

Figure S1. Cardiac EP4 expression is upregulated in response to isoproterenol (ISO) treatment.
A. The experimental scheme showing wild-type male mice were received ISO (5mg/kg/day, sc) for 7 days to induce cardiac fibrosis. **B**. Representative photographs of mouse hearts after treatment. The ratios of heart weight (HW, mg) to body weight (BW, g) were calculated. Scale bar=2mm. n=7-8 mice per group. **C&D**. Representative echocardiographic images and the measurements of left ventricle mass (LV Mass) in mice. Transverse scale bar=100ms. Vertical scale bar=2mm. n=5-8 mice per group. **E**. Representative images of peak velocity flow in early diastole (E, m/s) to peak velocity flow in late diastole by atrial contraction (A, m/s) in mice. The ratio of E/A was calculated. Transverse scale bar=100ms. Vertical scale bar=0.4m/s. n=5-7 mice per group. **F**. qRT-PCR analysis showing increased mRNA expression of collagen I and collagen III in the hearts of mice receiving ISO treatment. n=6 per group. **G**. qRT-PCR analysis demonstrating increased mRNA expression of COX-2 in mouse heart tissues. n=6 per group. **H&I**. The PGE_2 concentration in the culture medium of the cardiomyocytes (CMs) (**H**) and the cardiac fibroblasts (CFs) (**I**) treated with ISO was measured using an ELISA assay. n=5–6. **J**. qRT-PCR analysis showing upregulated mRNA expression of EP4 in the hearts of mice treated with ISO. n=6 per group. **K**. Representative immunohistochemical staining showing that the EP4 protein expression was increased in mouse left ventricles after ISO treatment. Scale bar=20μm. **L**. Schematic showing of the generation of fibroblast-specific tracing mouse line by crossing the Rosa26-tdTomato lineage tracing mouse with the S100A4-Cre mouse. **M**. Representative immunofluorescence assay of heart sections of the $tdTomato^{f/+}$-$S100A4^{Cre+}$ mice after 7 days' ISO treatment. Green, EP4 protein; Red, tdTomato-positive CFs. Scale bar=50μm. Data were presented as mean±SEM. *P<0.05, **P<0.01, ***P<0.001 by two-tailed unpaired t test (B, D, E, H-J) or by two-way ANOVA followed by the Sidak's multiple comparisons test (F and G).

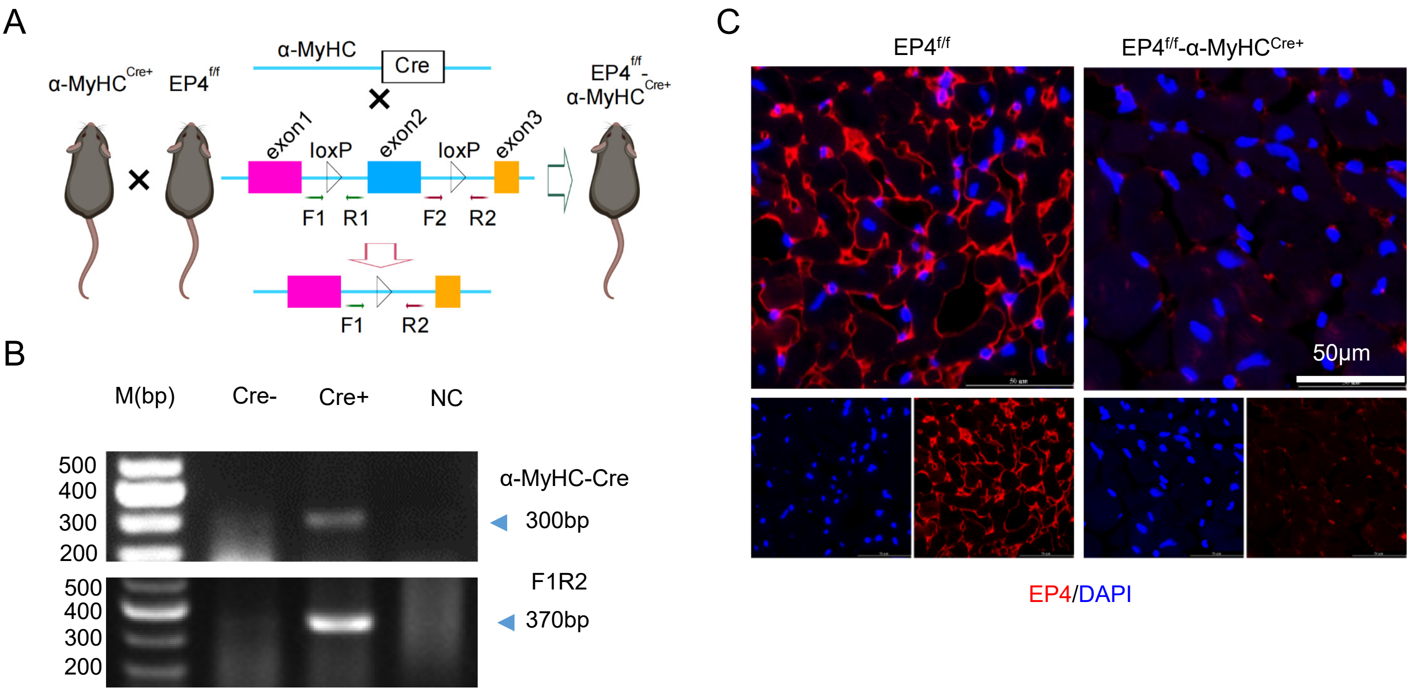

Figure S2. Generation of the cardiomyocyte (CM) EP4 gene specific knockout (EP4$^{f/f}$-α-MyHC^{Cre+}) mice.
A. Schematic showing of the generation of the EP4$^{f/f}$-α-MyHC^{Cre+} mice. The floxed EP4 allele (EP4$^{f/f}$) was modified by α-MyHC-Cre at two loxP sites flanking the exon 2 of the EP4 gene. The F1/F2 and R1/R2 are the primers designed to detect loxP sites. F1 and R2 are the primers designed to check the presence or absence of the exon 2. All primer sequences are shown in Table S2. **B**. Validation of the α-MyHC-Cre recombinase transgene (top, 300bp) and recombined floxed EP4 allele (bottom, 370bp) by PCR using mouse heart DNA. M: marker. NC: negative control. **C**. Immunofluorescence staining showing the EP4 protein (red) was markedly decreased in cardiomyocytes in the heart tissues of the EP4$^{f/f}$-α-MyHC^{Cre+} mice. DAPI stains the nuclei. Scale bar=50μm.

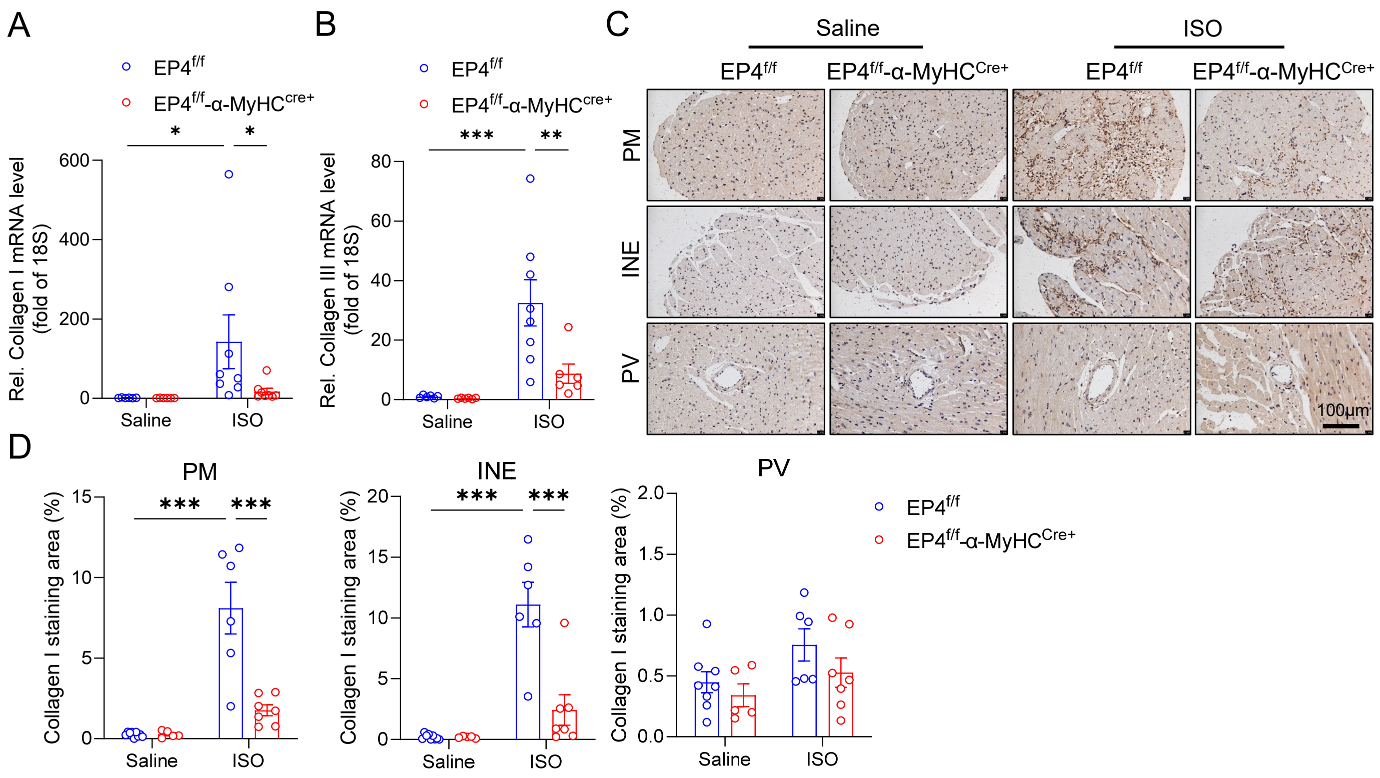

Figure S3. Deficiency of the CM-EP4 reduces ISO-induced collagen production.

A&B. qRT-PCR analysis showing the decreases in the collagen I (**A**) and collagen III (**B**) mRNA levels in the EP4$^{f/f}$-α-MyHC^{Cre+} mice compared to EP4$^{f/f}$ mice after 7 days treatment of ISO. n=6-8 per group. **C&D.** Representative images of immunohistochemical staining of collagen I in the male EP4$^{f/f}$ and EP4$^{f/f}$-α-MyHC^{Cre+} mouse hearts after 7 days' saline or ISO injection (**C**). The papillary muscle (PM) area, interstitial area near the endocardium (INE), and perivascular (PV) area pictures were shown, respectively. Quantitative analysis of collagen I-positive areas was performed by image J software (**D**). Scale bar=100μm. n=5-9 per group. Data were presented as mean±SEM. *P<0.05, **P<0.01, ***P<0.001 by two-way ANOVA followed by the Tukey's multiple comparisons test.

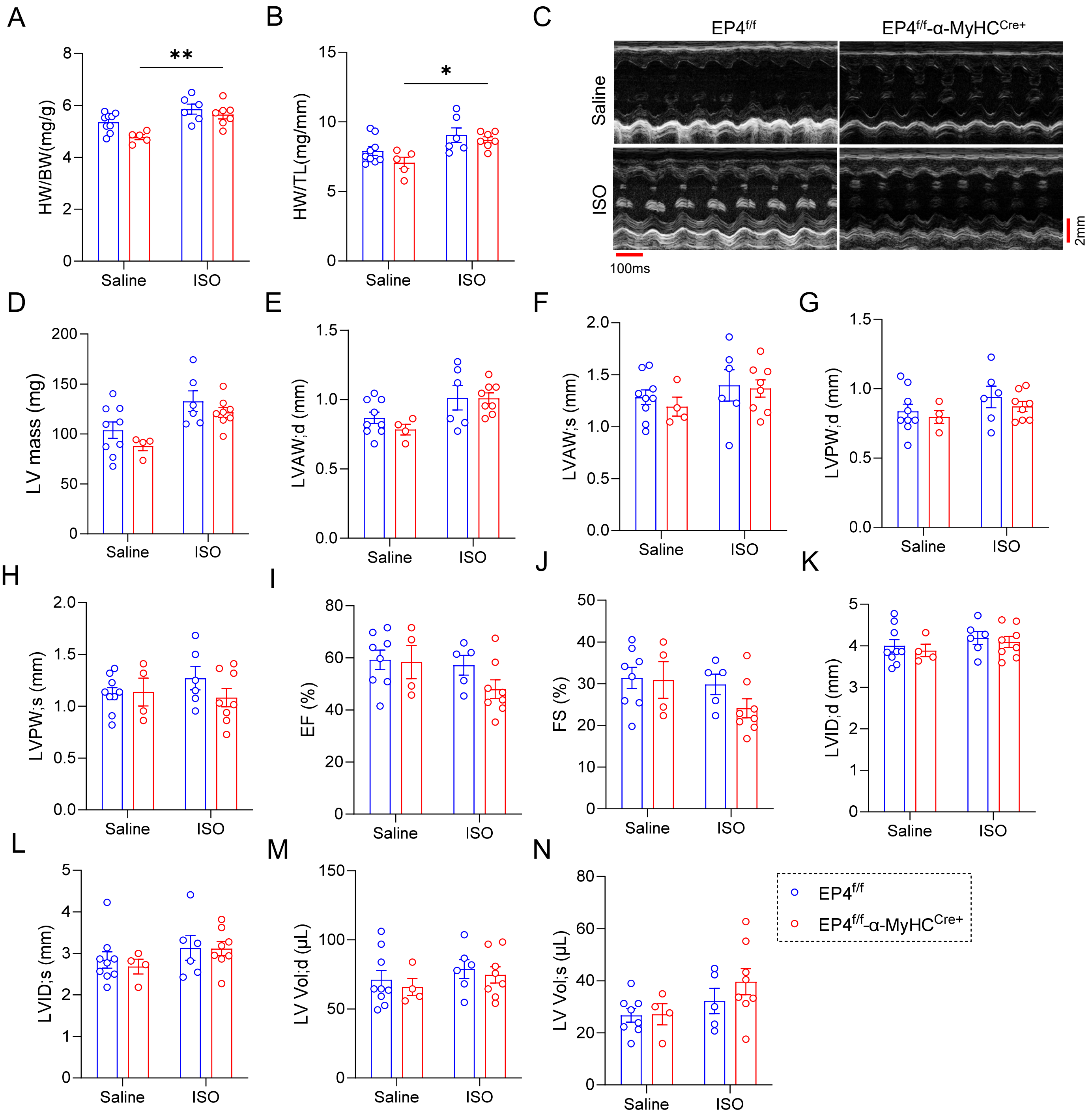

Figure S4. Deletion of the CM-EP4 has no significant impact on cardiac hypertrophy and systolic function. A&B. The heart weight (HW, mg) to body weight (BW, g) ratio (HW/BW) (**A**) and the HW (mg) to tibia length (TL, mm) ratio (HW/TL) (**B**) in male EP4$^{f/f}$ and EP4$^{f/f}$-α-MyHC^{Cre+} mice receiving ISO treatment for 7 days. n=5-9 mice per group. **C**. Representative M-mode echocardiography in male EP4$^{f/f}$ and EP4$^{f/f}$-α-MyHC^{Cre+} mice injected with saline or ISO for 7 days. Transverse scale bar=100ms. Vertical scale bar=2mm. **D-N**. Echocardiography measurements of left ventricular mass (LV mass) (**D**), left ventricular end-diastolic anterior wall thickness (LVAW;d) (**E**), left ventricular end-systolic anterior wall thickness (LVAW;s) (**F**), left ventricular end-diastolic posterior wall thickness (LVPW;d) (**G**), left ventricular end-systolic posterior wall thickness (LVPW;s) (**H**), ejection fraction (EF) (**I**), fractional shortening (FS) (**J**), left ventricular end-diastolic internal diameter (LVID;d) (**K**),left ventricular end-systolic internal diameter (LVID;s) (**L**), left ventricular end-diastolic volume (LV Vol;d) (**M**) and left ventricular end-systolic internal diameter (LV Vol;s) (**N**) in male EP4$^{f/f}$ and EP4$^{f/f}$-α-MyHC^{Cre+} mice injected saline or ISO for 7 days. n=4-9 mice per group. Data were presented as mean±SEM. *P<0.05, **P<0.01 by two-way ANOVA followed by the Tukey's multiple comparisons test.

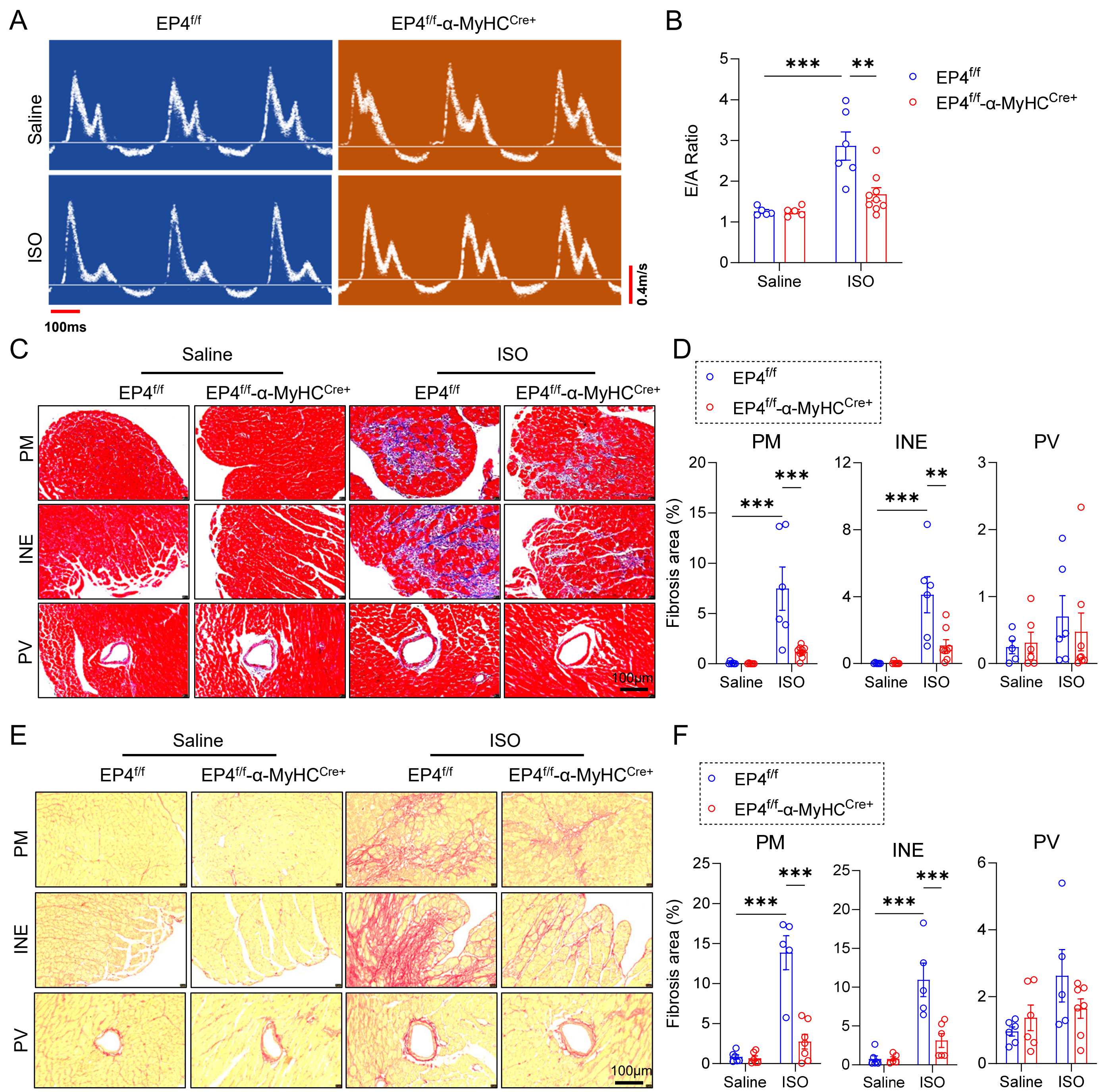

Figure S5. Deficiency of the CM-EP4 improves ISO-induced cardiac diastolic dysfunction and fibrosis in female mice.

A&B. Representative Doppler echocardiography of peak velocity flow in early diastole (E, m/s) to peak velocity flow in late diastole by atrial contraction (A, m/s) in female EP4$^{f/f}$ and EP4$^{f/f}$-α-MyHC^{Cre+} mice injected with saline or ISO for 7 days (**A**). The ratio of E/A was calculated (**B**). Transverse scale bar=100ms. Vertical scale bar=0.4m/s. n=5-9 per group. **C&D.** Representative images of Masson's trichrome staining of the hearts of female EP4$^{f/f}$ and EP4$^{f/f}$-α-MyHC^{Cre+} mice after 7 days' saline or ISO injection. The papillary muscle (PM) area, interstitial area near the endocardium (INE), and perivascular (PV) area were shown, respectively (**C**). Quantitative analysis of fibrotic areas (blue) was performed by the image J software (**D**). Scale bar=100μm. n=6-9 per group. **E&F.** Representative images of Sirius red staining of the hearts of female EP4$^{f/f}$ and EP4$^{f/f}$-α-MyHC^{Cre+} mice receiving ISO treatment for 7 days. The PM area, INE area, and PV area were shown, respectively (**E**). Quantitative analysis of fibrotic areas (red) was performed by the image J software (**F**). Scale bar=100μm. n=5-7 per group. Data were presented as mean±SEM. **P<0.01, ***P<0.001 by two-way ANOVA followed by the Tukey's multiple comparisons test.

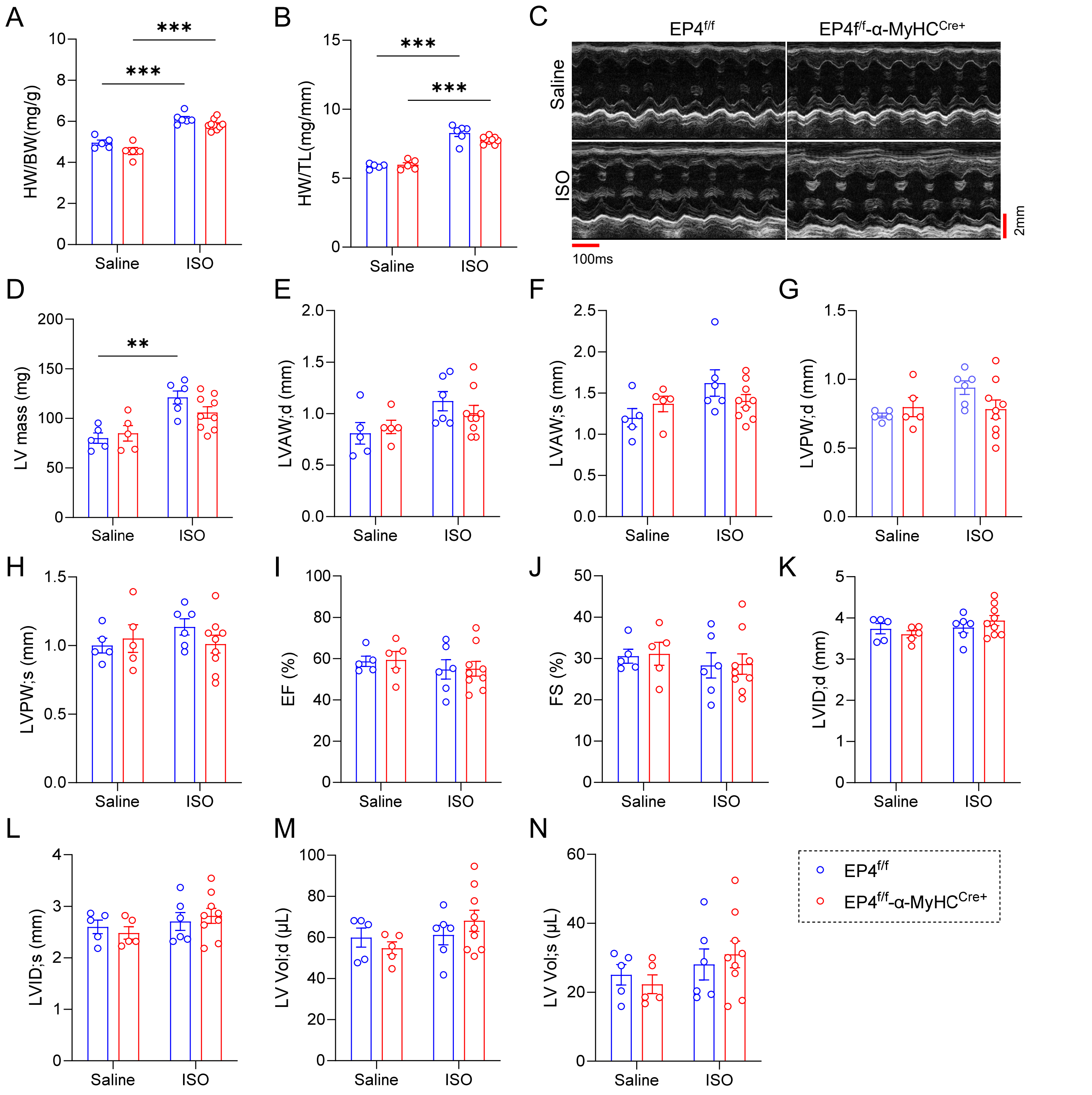

Figure S6. ISO-induced cardiac hypertrophy and systolic function is comparable between female EP4$^{f/f}$ and EP4$^{f/f}$-α-MyHC^{Cre+} mice.

A&B. The heart weight (HW, mg) to body weight (BW, g) ratio (HW/BW) (**A**) and the HW (mg) to tibia length (TL, mm) ratio (HW/TL) (**B**) in female EP4$^{f/f}$ and EP4$^{f/f}$-α-MyHC^{Cre+} mice receiving saline or ISO injection for 7 days. n=5-9 mice per group. **C.** Representative M-mode echocardiography in female EP4$^{f/f}$ and EP4$^{f/f}$-α-MyHC^{Cre+} mice injected with saline or ISO for 7 days. Transverse scale bar=100ms. Vertical scale bar=2mm. **D-N.** Echocardiography measurements of left ventricular mass (LV mass) (**D**), left ventricular end-diastolic anterior wall thickness (LVAW;d) (**E**), left ventricular end-systolic anterior wall thickness (LVAW;s) (**F**), left ventricular end-diastolic posterior wall thickness (LVPW;d) (**G**), left ventricular end-systolic posterior wall thickness (LVPW;s) (**H**), ejection fraction (EF) (**I**), fractional shortening (FS) (**J**), left ventricular end-diastolic internal diameter (LVID;d) (**K**) and left ventricular end-systolic internal diameter (LVID;s) (**L**), left ventricular end-diastolic volume (LV Vol;d) (**M**) and left ventricular end-systolic internal diameter (LV Vol;s) (**N**) in female EP4$^{f/f}$ and EP4$^{f/f}$-α-MyHC^{Cre+} mice after saline or ISO injection for 7 days . n=5-9 mice per group. Data were presented as mean±SEM. **P<0.01, ***P<0.001 by two-way ANOVA followed by the Tukey's multiple comparisons test.

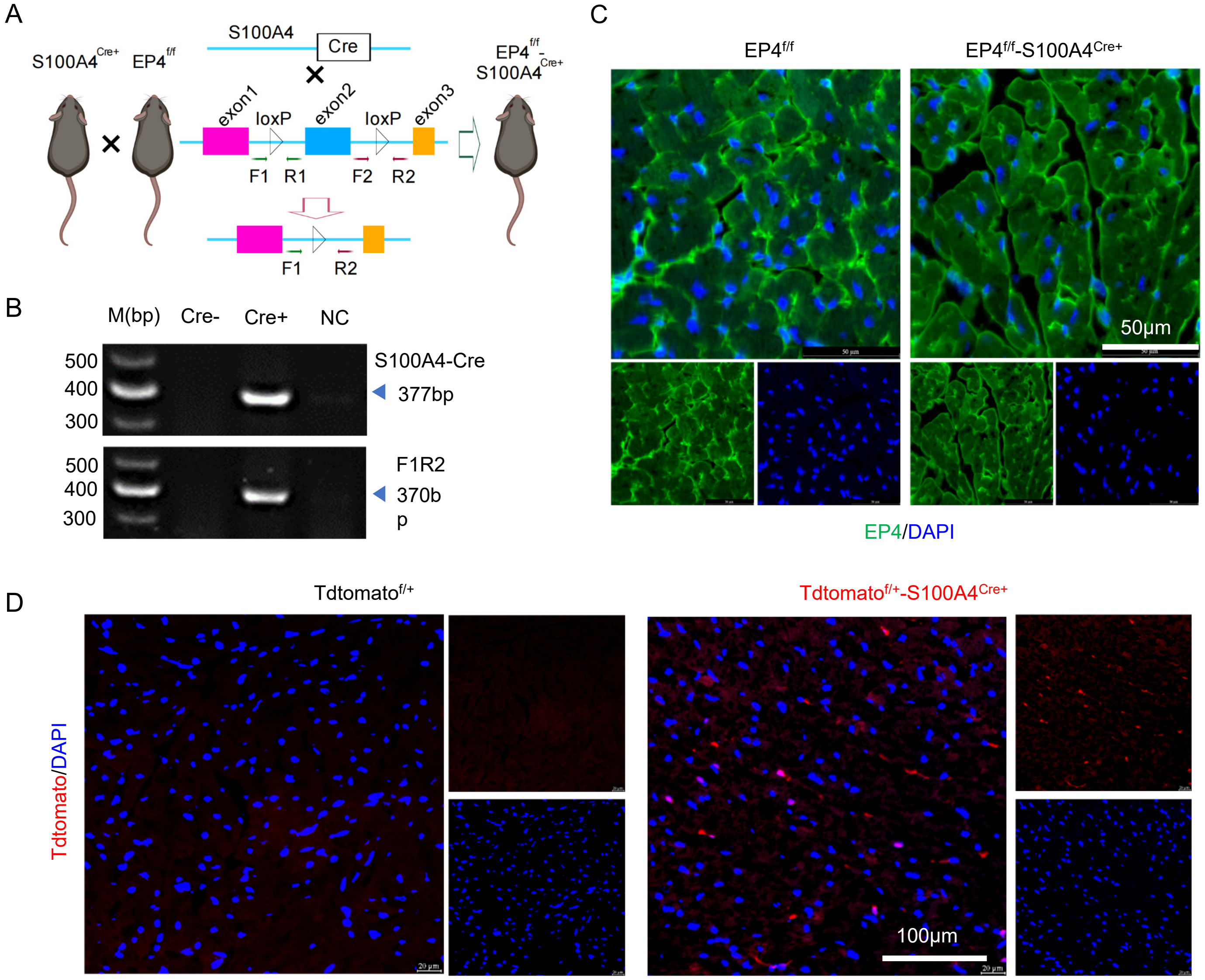

Figure S7. Generation of the EP4$^{f/f}$-S100A4^{Cre+} mice.

A. Schematic showing of the generation of the EP4$^{f/f}$-S100A4^{Cre+} mice. Floxed EP4 allele (EP4$^{f/f}$) was modified by S100A4-Cre at two loxP sites flanking the exon 2 of the EP4 gene. The F1/F2 and R1/R2 are the primers designed to detect loxP sites. F1 and R2 are the primers designed to check the presence or absence of the exon 2. All primer sequences are shown in Table S1. **B**. Validation of the S100A4-Cre recombinase transgene (top, 377bp) and recombined floxed EP4 allele (bottom, 370bp) by PCR using mouse heart DNA. M: marker. NC: negative control. **C**. Immunofluorescence staining showing the EP4 protein (green) was decreased in the interstitial area in the EP4$^{f/f}$-S100A4^{Cre+} mouse heart tissue. DAPI stains the nuclei. Scale bar=50μm. **D**. Representative sections of the tdTomato$^{f/+}$ and tdTomato$^{f/+}$-S100A4^{Cre+} mouse hearts. tdTomato (Red) indicates the CFs. Scale bar=100μm.

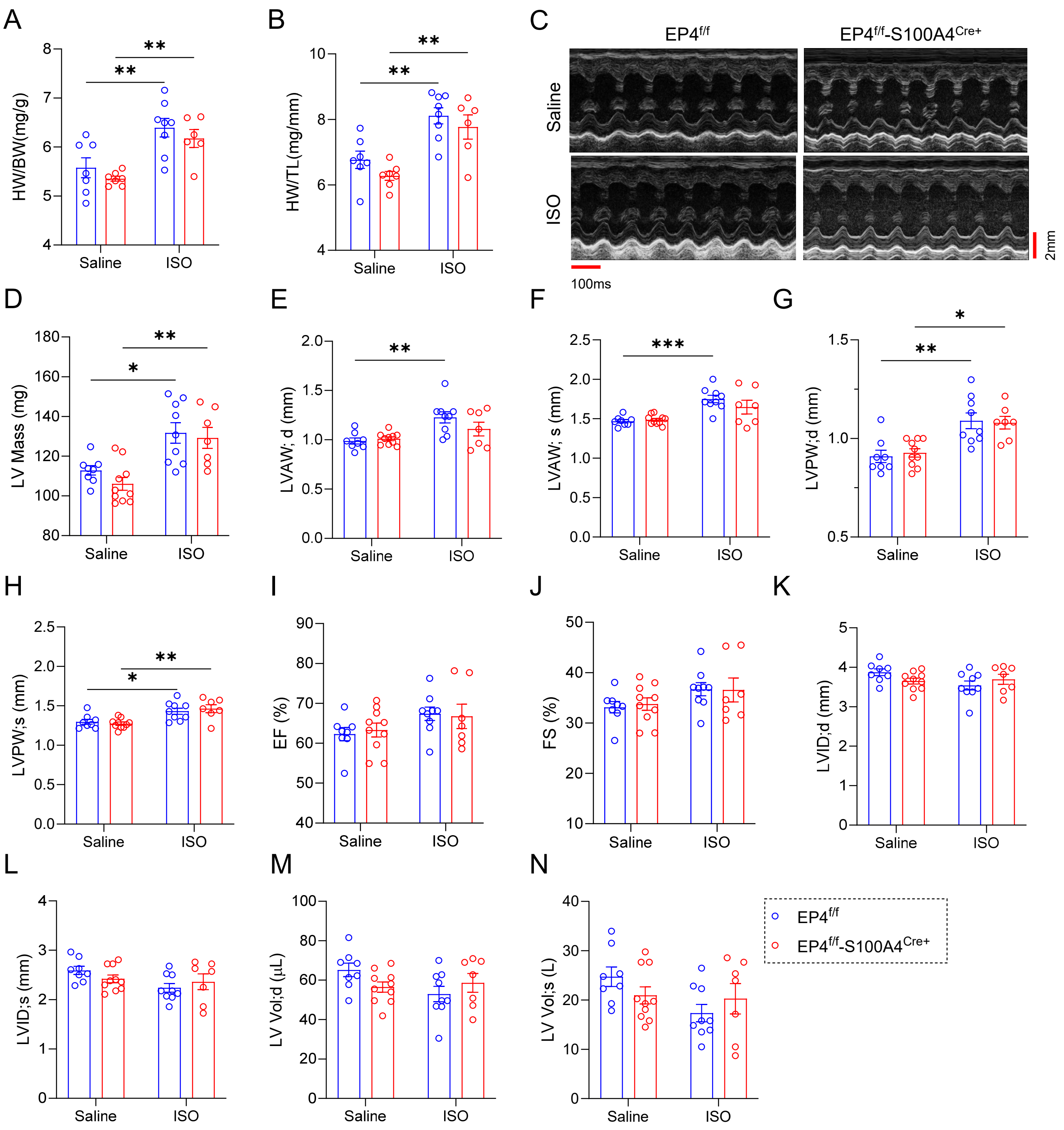

Figure S8. Deletion of the CF-EP4 has no significant impact on cardiac hypertrophy and systolic function.
A&B. The heart weight (HW, mg) to body weight (BW, g) ratio (HW/BW) (**A**) and the HW (mg) to tibia length (TL, mm) ratio (HW/TL) (**B**) were increased in mice receiving ISO treatment for 7 days compared with that in the mice treated with saline. No difference was observed between two genotypes. n=6-8 mice per group. **C.** Representative echocardiographic images in the $EP4^{f/f}$ and $EP4^{f/f}$-$S100A4^{Cre+}$ male mice treated with saline or ISO for 7 days. Transverse scale bar=100ms. Vertical scale bar=2mm. **D-N**. Echocardiography measurements of left ventricular mass (LV mass) (**D**), left ventricular end-diastolic anterior wall thickness (LVAW;d) (**E**), left ventricular end-systolic anterior wall thickness (LVAW;s) (**F**), left ventricular end-diastolic posterior wall thickness (LVPW;d) (**G**), left ventricular end-systolic posterior wall thickness (LVPW;s) (**H**), ejection fraction (EF) (**I**), fractional shortening (FS) (**J**), left ventricular end-diastolic internal diameter (LVID;d) (**K**),left ventricular end-systolic internal diameter (LVID;s) (**L**), left ventricular end-diastolic volume (LV Vol;d) (**M**) and left ventricular end-systolic internal diameter (LV Vol;s) (**N**) in male $EP4^{f/f}$ and $EP4^{f/f}$-α-$MyHC^{Cre+}$ mice injected saline or ISO for 7 days.
n=7-10 mice per group. Data were presented as mean±SEM. *P<0.05, **P<0.01, ***P<0.001 by two-way ANOVA followed by the Tukey's multiple comparisons test.

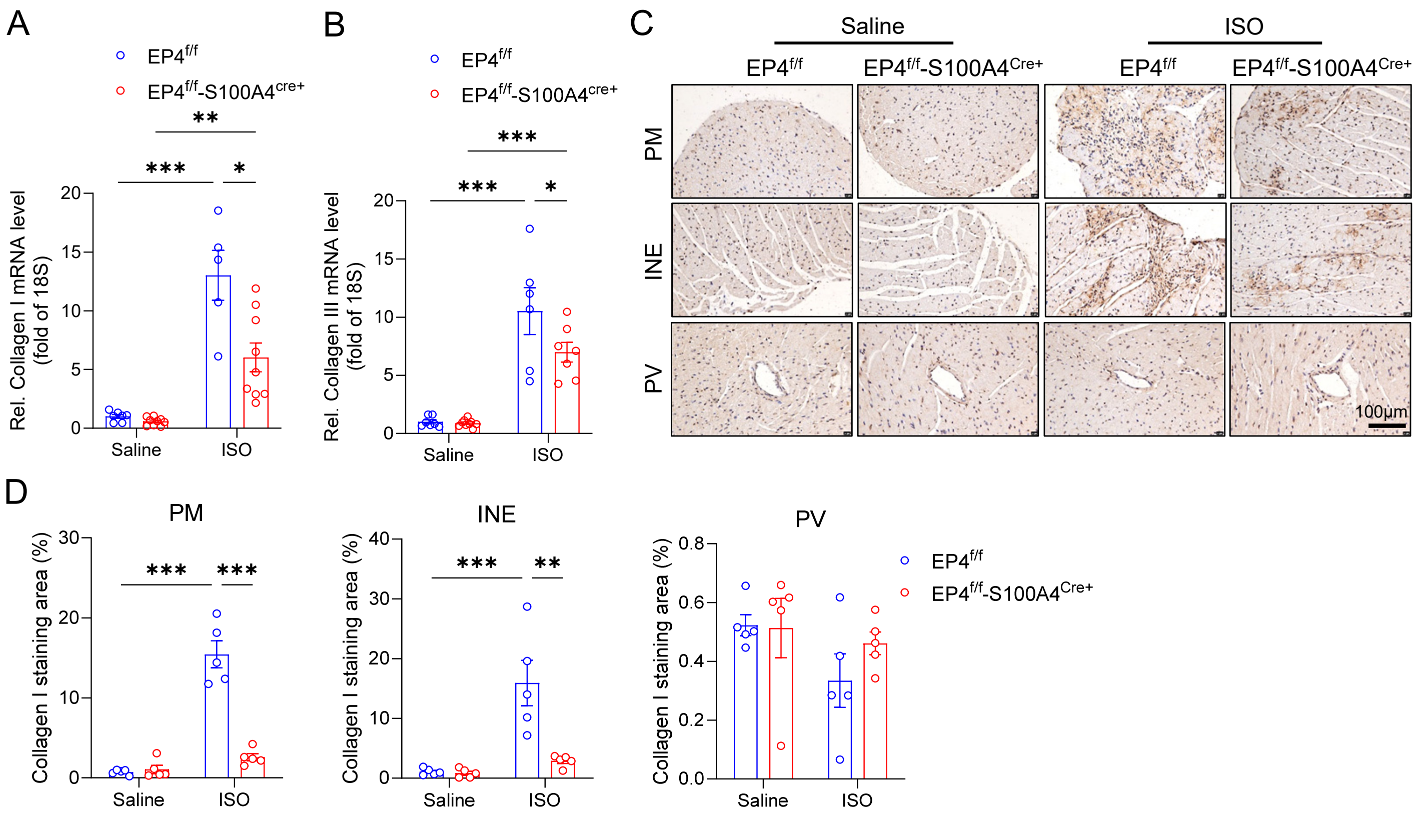

Figure S9. Deletion of the CF-EP4 suppresses ISO-induced collagen synthesis.

A&B. qRT-PCR analysis showing the reduction of the collagen I (**A**) and collagen III (**B**) mRNA levels in the hearts of the male EP4$^{f/f}$-S100A4^{Cre+} mice after 7 days' injection of ISO. n=5-9 per group. **C&D.** Representative images of immunohistochemical staining of collagen I in the hearts of the EP4$^{f/f}$ and EP4$^{f/f}$-S100A4^{Cre+} mice after 7 days' saline or ISO injection (**C**). The papillary muscle (PM) area, interstitial area near the endocardium (INE), and perivascular (PV) area were shown, respectively. Quantitative analysis of collagen I-positive areas was performed by image J software (**D**). Scale bar=100μm. n=5 to 9 per group. Data were presented as mean±SEM. *P<0.05, **P<0.01, ***P<0.001 by two-way ANOVA followed by the Tukey's multiple comparisons test.

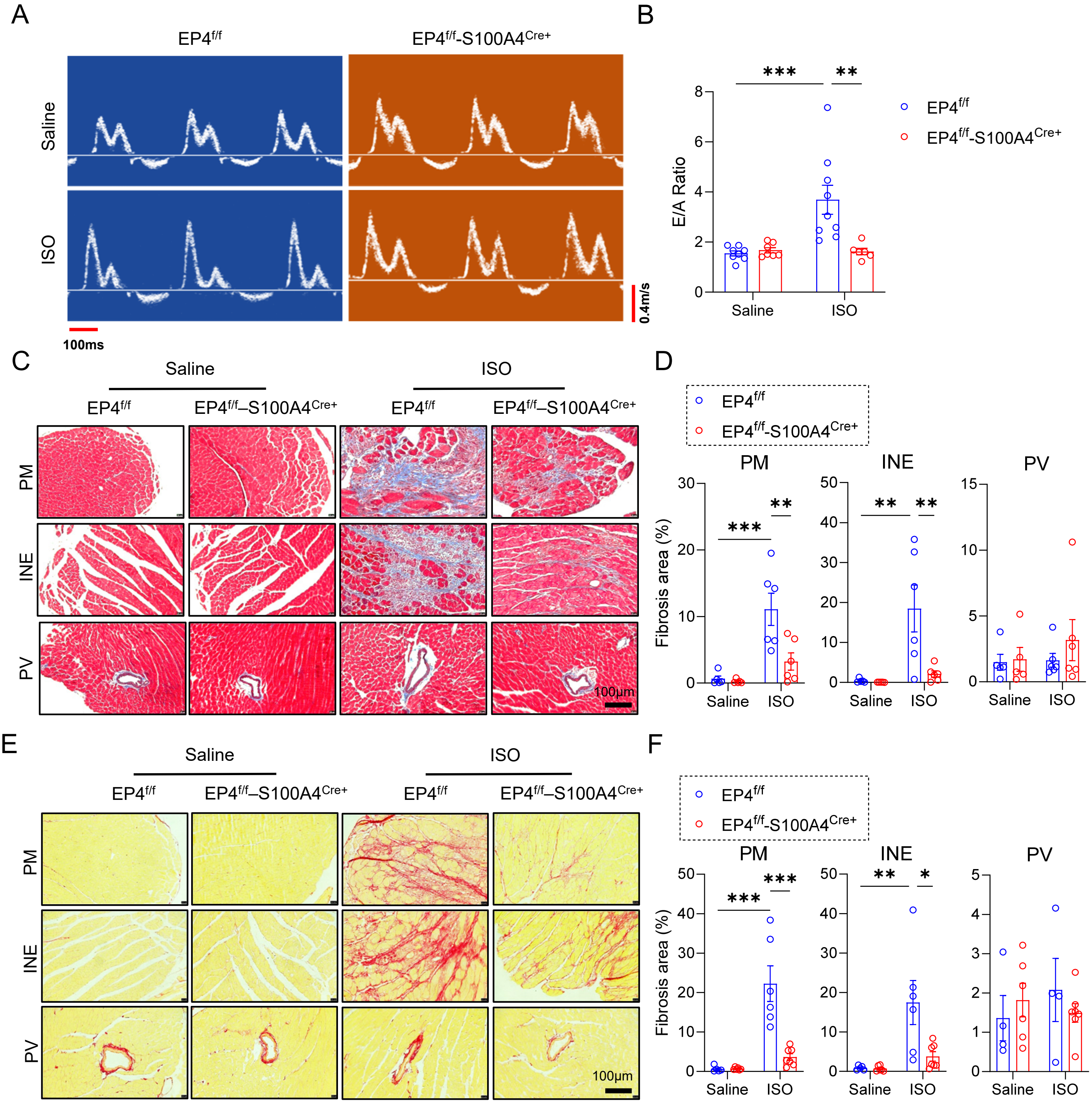

Figure S10. Deletion of the CF-EP4 suppresses ISO-induced cardiac diastolic dysfunction and fibrosis in female mice.

A&B. Representative Doppler echocardiography of peak velocity flow in early diastole (E, m/s) to peak velocity flow in late diastole by atrial contraction (A, m/s) in female $EP4^{f/f}$ and $EP4^{f/f}$-$S100A4^{Cre+}$ mice injected with saline or ISO for 7 days (**A**). The ratio of E/A was calculated (**B**). Transverse scale bar=100ms. Vertical scale bar=0.4m/s. n=6-8 mice per group. **C&D.** Representative images of Masson's trichrome staining of the hearts of female $EP4^{f/f}$ and $EP4^{f/f}$-$S100A4^{Cre+}$ mice receiving 7 days' saline or ISO injection. Papillary muscle (PM) area, interstitial area near the endocardium (INE), and perivascular (PV) area were shown, respectively (**C**). Quantitative analysis of fibrotic areas (blue) was performed by image J software (**D**). Scale bar=100μm. n=5-6 per group. **E&F.** Representative images of Sirius red staining of the hearts of female $EP4^{f/f}$ and $EP4^{f/f}$-$S100A4^{Cre+}$ mice receiving 7 days' saline or ISO injection. PM area, INE, and PV area were shown, respectively (**E**). Quantitative analysis of fibrotic areas (red) was performed by image J software (**F**). Scale bar=100μm. n=5-7 per group. Data were presented as mean±SEM. *$P<0.05$, **$P<0.01$, ***$P<0.001$ by two-way ANOVA followed by the Tukey's multiple comparisons test.

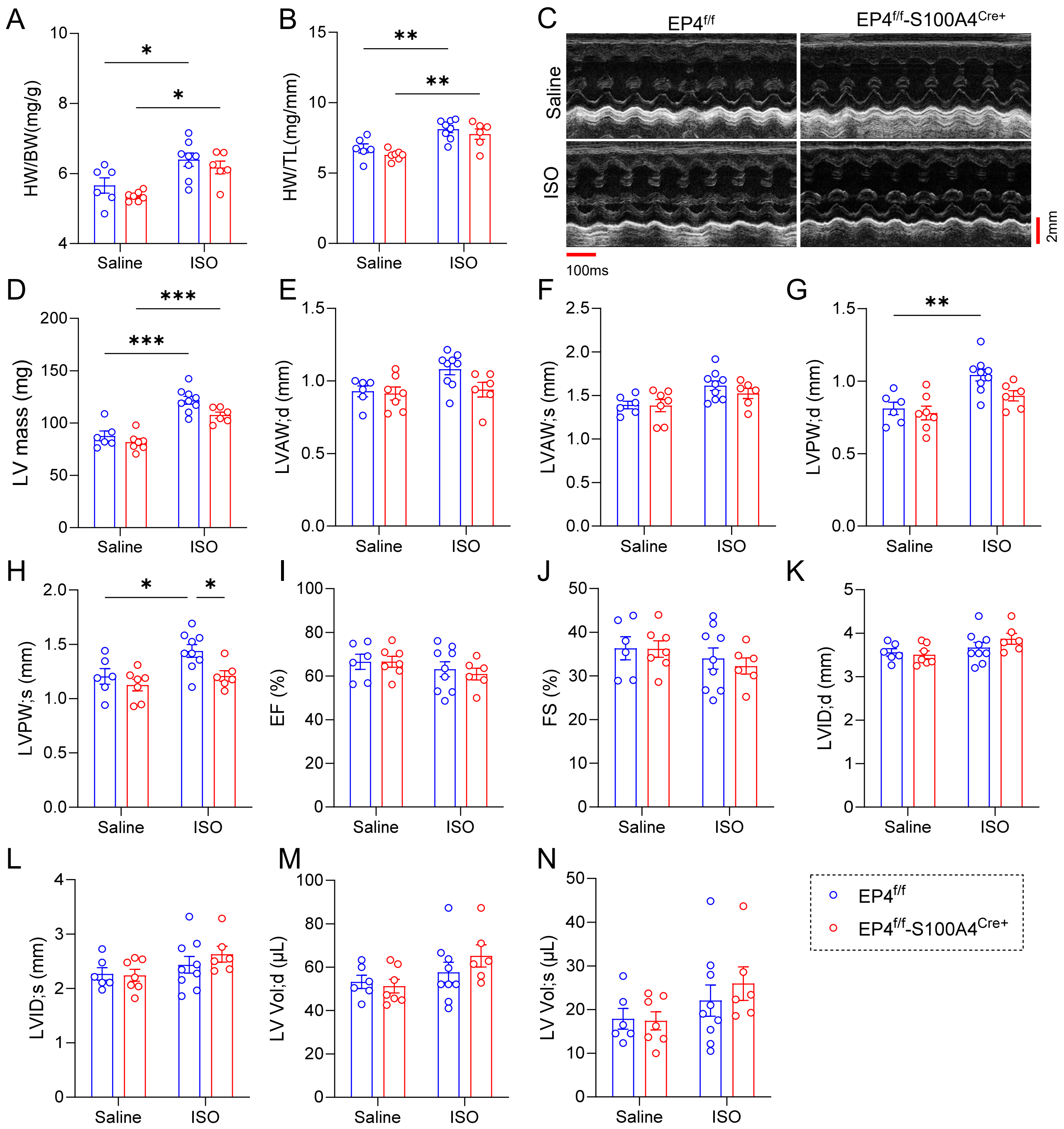

Figure S11. Deletion of the CF-EP4 has no effect on cardiac hypertrophy and systolic function in female mice. A&B. The heart weight (HW, mg) to body weight (BW, g) ratio (HW/BW) (**A**) and the HW (mg) to tibia length (TL, mm) ratio (HW/TL) (**B**) in female $EP4^{f/f}$ and $EP4^{f/f}$-$S100A4^{Cre+}$ mice receiving saline or ISO injection for 7 days. n=6-8 mice per group. **C**. Representative M-mode echocardiography in female $EP4^{f/f}$ and $EP4^{f/f}$-$S100A4^{Cre+}$ mice injected with saline or ISO for 7 days. Transverse scale bar=100ms. Vertical scale bar=2mm. **D-N**. Echocardiography measurements of left ventricular mass (LV mass) (**D**), left ventricular end-diastolic anterior wall thickness (LVAW;d) (**E**), left ventricular end-systolic anterior wall thickness (LVAW;s) (**F**), left ventricular end-diastolic posterior wall thickness (LVPW;d) (**G**), left ventricular end-systolic posterior wall thickness (LVPW;s) (**H**), ejection fraction (EF) (**I**), fractional shortening (FS) (**J**), left ventricular end-diastolic internal diameter (LVID;d) (**K**), left ventricular end-systolic internal diameter (LVID;s) (**L**), left ventricular end-diastolic volume (LV Vol;d) (**M**) and left ventricular end-systolic internal diameter (LV Vol;s) (**N**) in female $EP4^{f/f}$ and $EP4^{f/f}$-$S100A4^{Cre+}$ mice after 7 days' saline or ISO injection. n=6-9 mice per group. Data were presented as mean±SEM. *P<0.05, **P<0.01, ***P<0.001 by two-way ANOVA followed by the Tukey's multiple comparisons test.

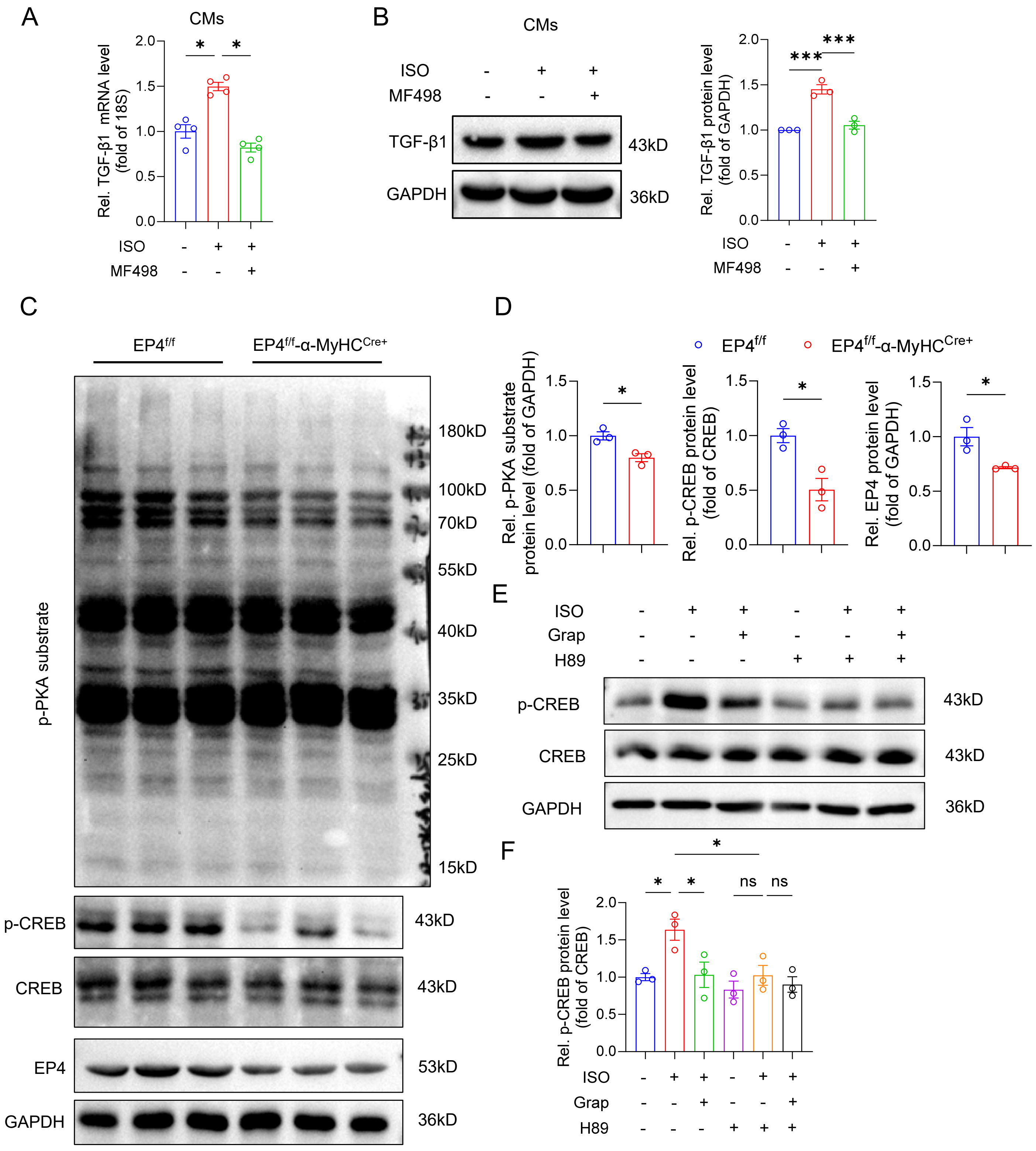

Figure S12. Blockade of EP4 suppresses TGF-β1 expression and inhibits ISO-induced CREB phosphorylation in CMs.

A. qRT-PCR analysis of the mRNA expression of TGF-β1 in primary cultured neonatal rat cardiomyocytes (NRCMs) pretreated with MF498 (0.1μM) for 30 min, followed by ISO (20μM) for 24 hours. n=4. **B**. Western blot assay showing the protein expression of TGF-β1 in NRCMs pretreated with MF498 (0.1μM) for 30 min, followed by ISO (20μM) treatment for 24 hours. The quantitative analysis of TGF-β1 protein level was performed by image J software. n=3. **C&D**. The cardiomyocytes were freshly isolated from $EP4^{f/f}$ and $EP4^{f/f}$-α-$MyHC^{Cre+}$ mice and treated with ISO (20μM) for 30 min. Western blot was performed to evaluate phosphorylated PKA substrate (p-PKA-substrate), phosphorylated CREB (p-CREB), total CREB, and EP4 protein levels. The quantitative analysis of these protein levels was carried out using image J software. n=3. **E&F**. Western blot assay showing the protein expression of p-CREB in NRCMs pretreated with grapiprant (1μM) for 30 min, followed by ISO (20μM) treatment for 30 min in the presence or absence of H89 (20μM). The quantitative analysis of p-CREB protein level was performed by image J software. n=3. Data were presented as mean±SEM. *P<0.05 by two-tailed unpaired t test (D) or by one-way ANOVA followed by the Tukey's multiple comparisons test (A, B, and F).

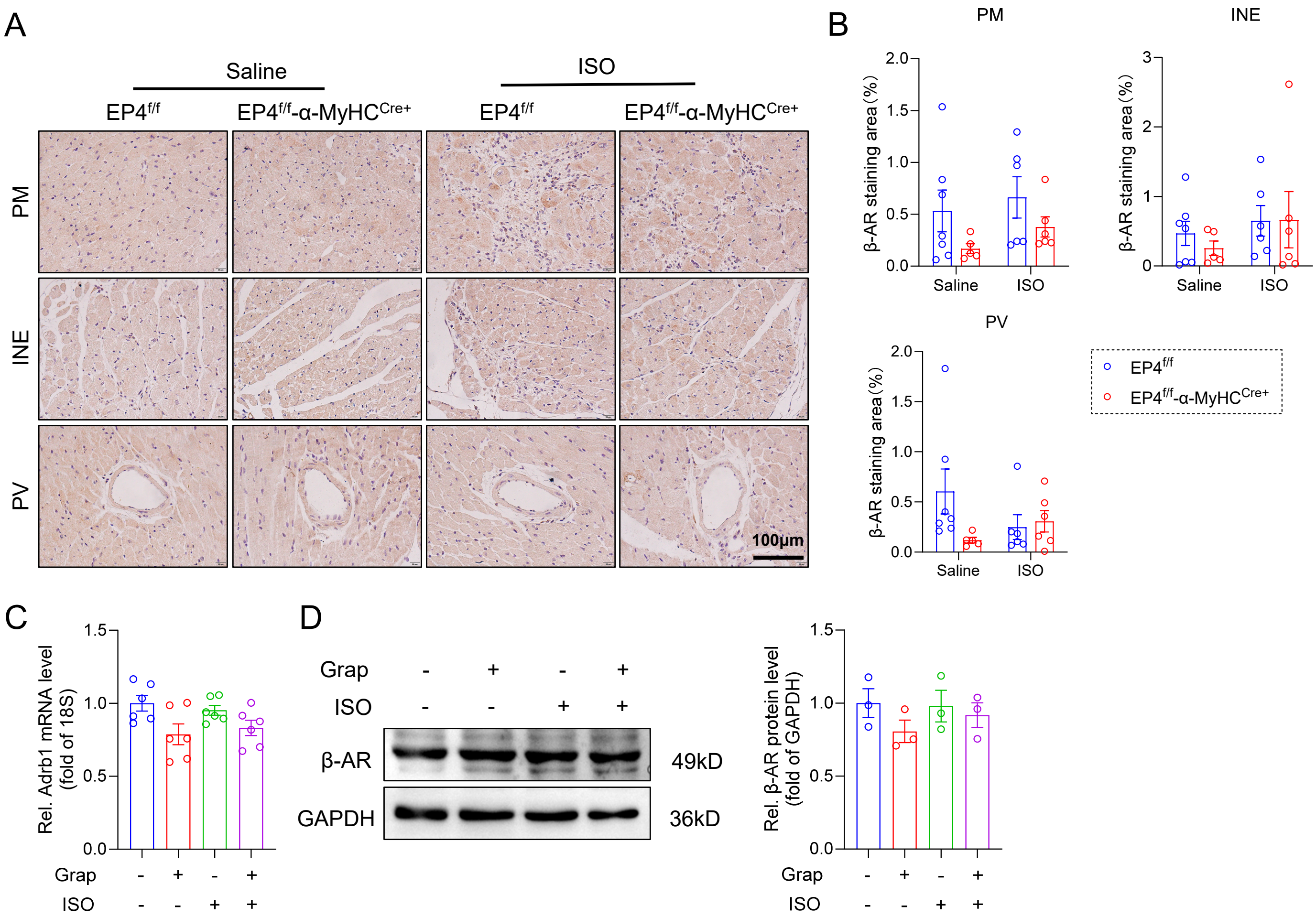

Figure S13. EP4 deletion or inhibition does not impact β-AR expression in CMs.

A&B. Immunohistochemical staining of β-AR in the hearts of the male $EP4^{f/f}$ and $EP4^{f/f}$-α-$MyHC^{Cre+}$ mice after treatment with saline or ISO for 7 days. Papillary muscle (PM) area, interstitial area near the endocardium (INE), and perivascular (PV) area were shown, respectively (**A**). Quantitative analysis of β-AR-positive areas was performed by image J software (**B**). Scale bar=100μm. n=5-7 per group. **C.** qRT-PCR analysis was performed to evaluate the mRNA expression of Adrb1 (the gene encoding β-AR) in NRCMs pretreated with or without grapiprant (1μM) for 30 min, followed by treatment with ISO (20μM) for 6 hours. n=6. **D**. Western blot assay showing the protein expression of β-AR in NRCMs pretreated with grapiprant (1μM) for 30 min, followed by ISO (20μM) treatment for 24 hours. The quantitative analysis of β-AR protein level was performed by image J software. n=3. Data were presented as mean±SEM.

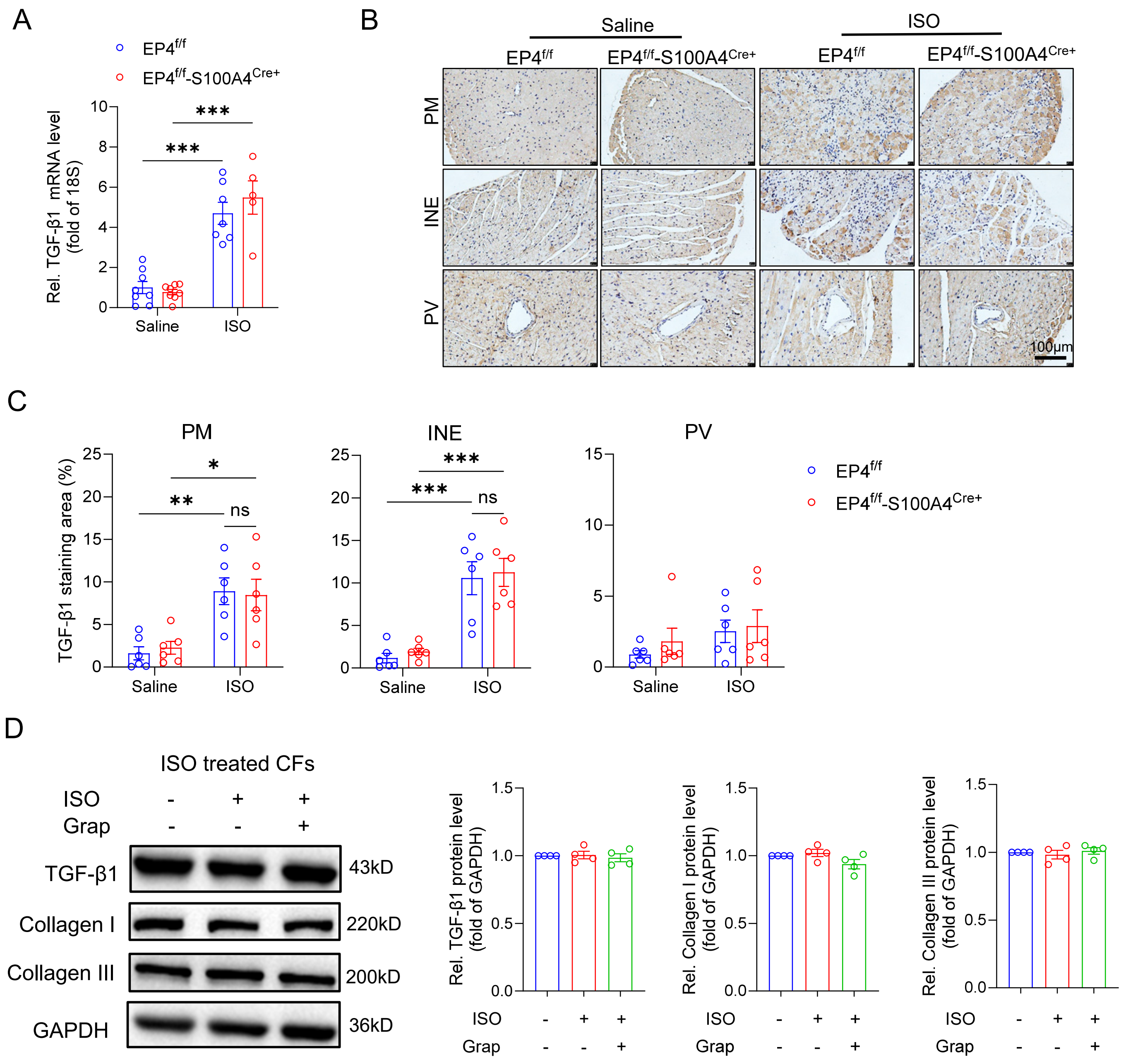

Figure S14. EP4 increases TGF-β1 expression in the CMs rather than in the CFs.

A. qRT-PCR analysis of the mRNA expression of TGF-β1 in the hearts of the male EP4$^{f/f}$ and EP4$^{f/f}$-S100A4^{Cre+} mice after 7 days' ISO injection. n=5-8 per group. **B&C**. Immunohistochemical staining of TGF-β1 in the hearts of the male EP4$^{f/f}$ and EP4$^{f/f}$-S100A4^{Cre+} mice after treatment with saline or ISO for 7 days. Papillary muscle (PM) area, interstitial area near the endocardium (INE), and perivascular (PV) area were shown, respectively (**B**). Quantitative analysis of TGF-β1-positive areas was performed by image J software (**C**). Scale bar=100μm. n=6 per group. **D.** Western blot analysis of the expression of TGF-β1, collagen I and collagen III in the NRCFs pretreated with grapiprant (1μM) for 30 min, followed by ISO for 24 hours. The quantitative analysis of the protein levels was performed by image J software. n=4. Data were presented as mean±SEM. *P<0.05, **P<0.01, ***P<0.001 by two-way ANOVA followed by the Tukey's multiple comparisons test (A and C).

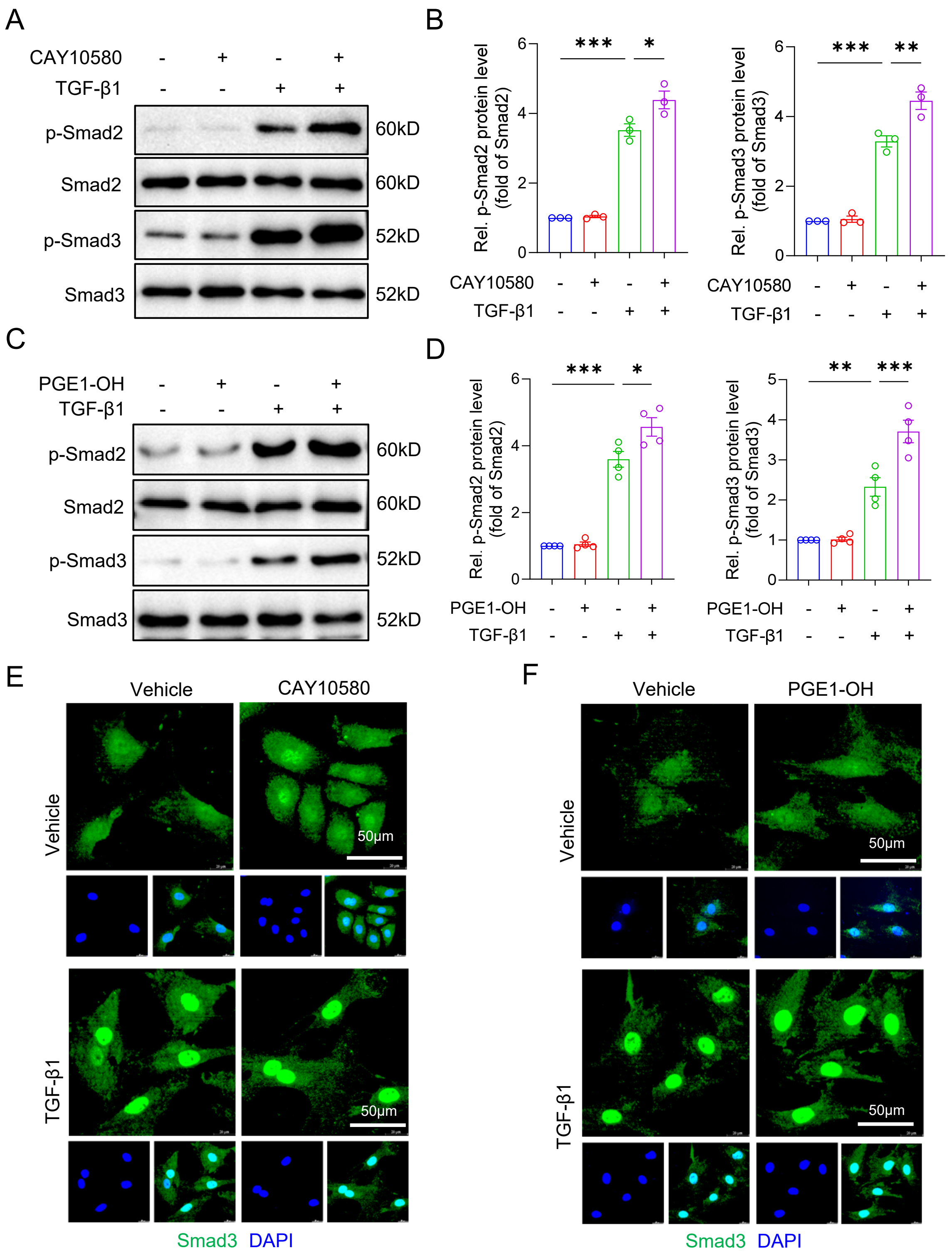

Figure S15. EP4 enhances TGF-β1-triggered phosphorylation of Smad2/3 and nuclear translocation of Smad3. A&B. Western blot analysis of the total and phosphorylated Smad2 and Smad3 levels in primary cultured neonatal rat cardiac fibroblasts (NRCFs) pretreated with or without CAY10580 (1μM) for 30 min, followed by TGF-β1 (5ng/mL) treatment for 3 hours. Quantitative analysis was performed using image J software (**B**). n=3. **C&D**. Western blot analysis of the total and phosphorylated Smad2 and Smad3 levels in the NRCFs pretreated with or without PGE1-OH (1μM) for 30 min, followed by TGF-β1 (5ng/mL) treatment for 3 hours. Quantitative analysis was performed using image J software (**D**). n=4. **E**. Immunofluorescence staining showing the nuclear translocation of Smad3 (green) in primary cultured NRCFs pretreated with CAY10580 (1μM) for 30 min, followed by TGF-β1 (5ng/mL) treatment for 3 hours. DAPI (blue) staining showed the nucleus. Scale bar=50μm. **F**. Immunofluorescence staining showing the nuclear translocation of Smad3 (green) in primary cultured NRCFs pretreated with PGE1-OH (1μM) for 30 min, followed by TGF-β1 (5ng/mL) treatment for 3 hours. DAPI (blue) staining showed the nucleus. Scale bar=50μm. Data were presented as mean±SEM. *P<0.05, **P<0.01, ***P<0.001 by one-way ANOVA followed by the Tukey's multiple comparisons test.

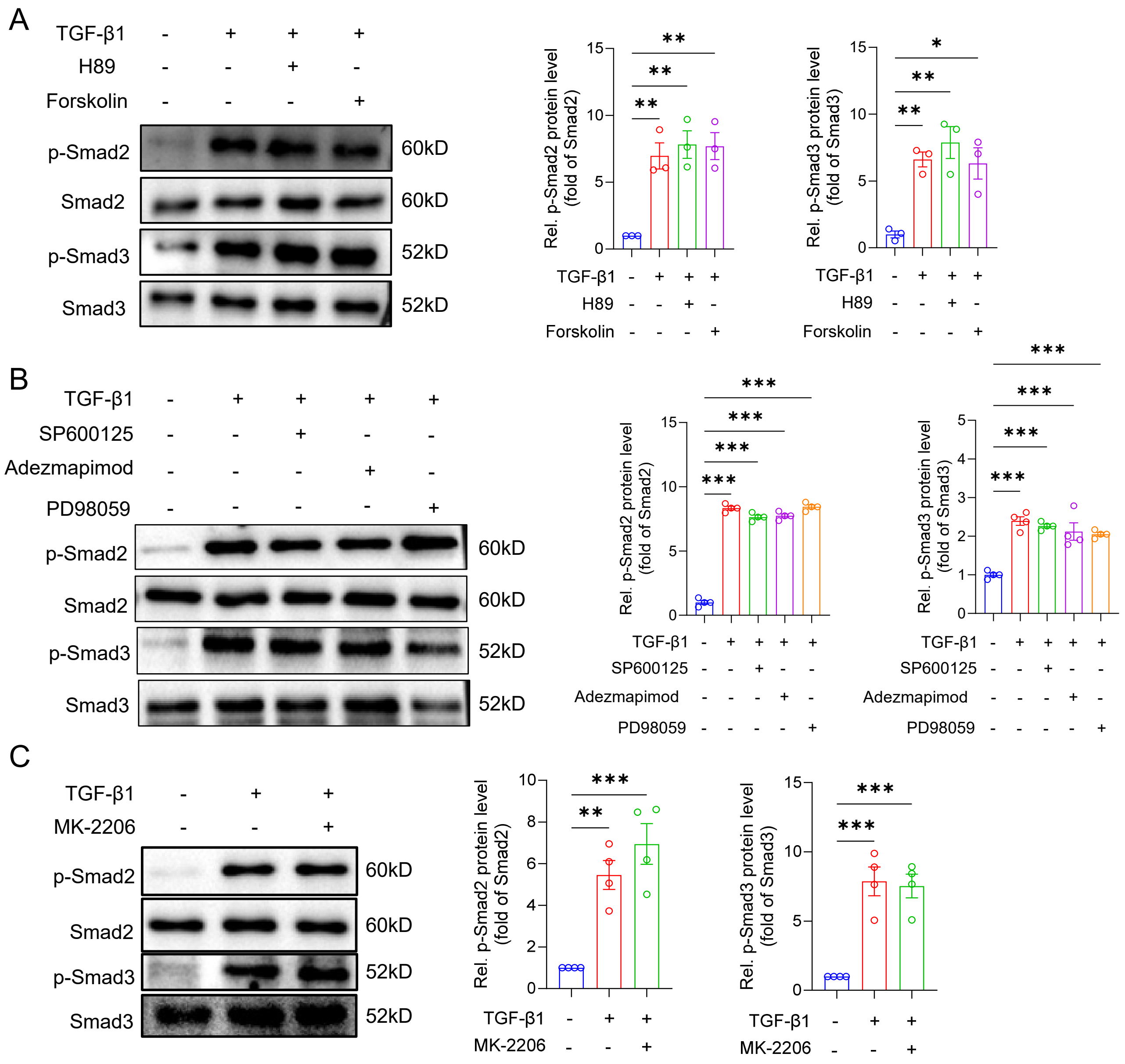

Figure S16. The PKA, MAPK, and AKT signaling pathways have no effect on TGF-β1-induced Smad2/3 activation in the NRCFs.

A. Western blot analysis of the total and phosphorylated Smad2 and Smad3 levels in primary cultured NRCFs pretreated with H89 (20μM) or forskolin (20μM) for 30 min, followed by TGF-β1 (10ng/mL) treatment for 3 hours. Quantitative analysis was performed using image J software. n=3. **B.** Western blot analysis of the total and phosphorylated Smad2 and Smad3 levels in primary cultured NRCFs pretreated with SP600125 (20μM), Adezmapimod (20μM), or PD98059 (10μM) for 30 min and then treated with TGF-β1 (10ng/mL) for 3 hours. Quantitative analysis was performed using image J software. n=4. **C.** Western blot analysis of the total and phosphorylated Smad2 and Smad3 levels in primary cultured NRCFs pretreated with MK-2206 (10μM) for 30 min, followed by TGF-β1 (10ng/mL) treatment for 3 hours. Quantitative analysis was performed using image J software. n=4. Data were presented as mean±SEM. *P<0.05, **P<0.01, ***P<0.001 by one-way ANOVA followed by the Tukey's multiple comparisons test.

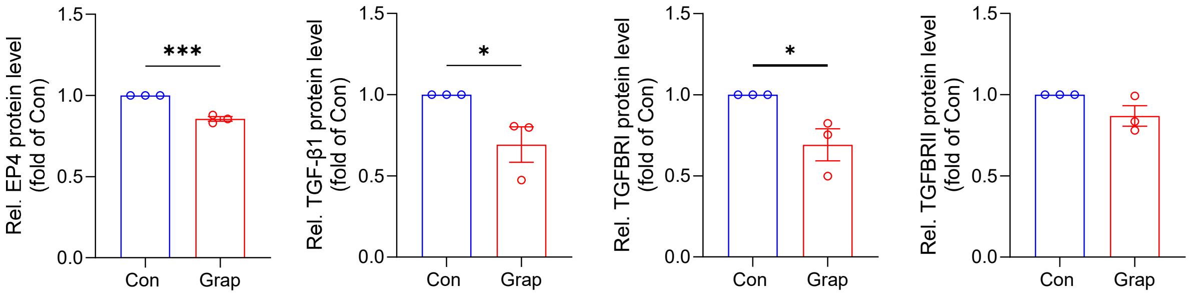

Figure S17. The quantitative analysis of IP blots in Figure 5J.

The Image J software was used to analyze the grayscale values of the bands, with the Control group subjected to normalization. n=3. Data were presented as mean±SEM. *P<0.05, ***P<0.001 by two-tailed unpaired t test.

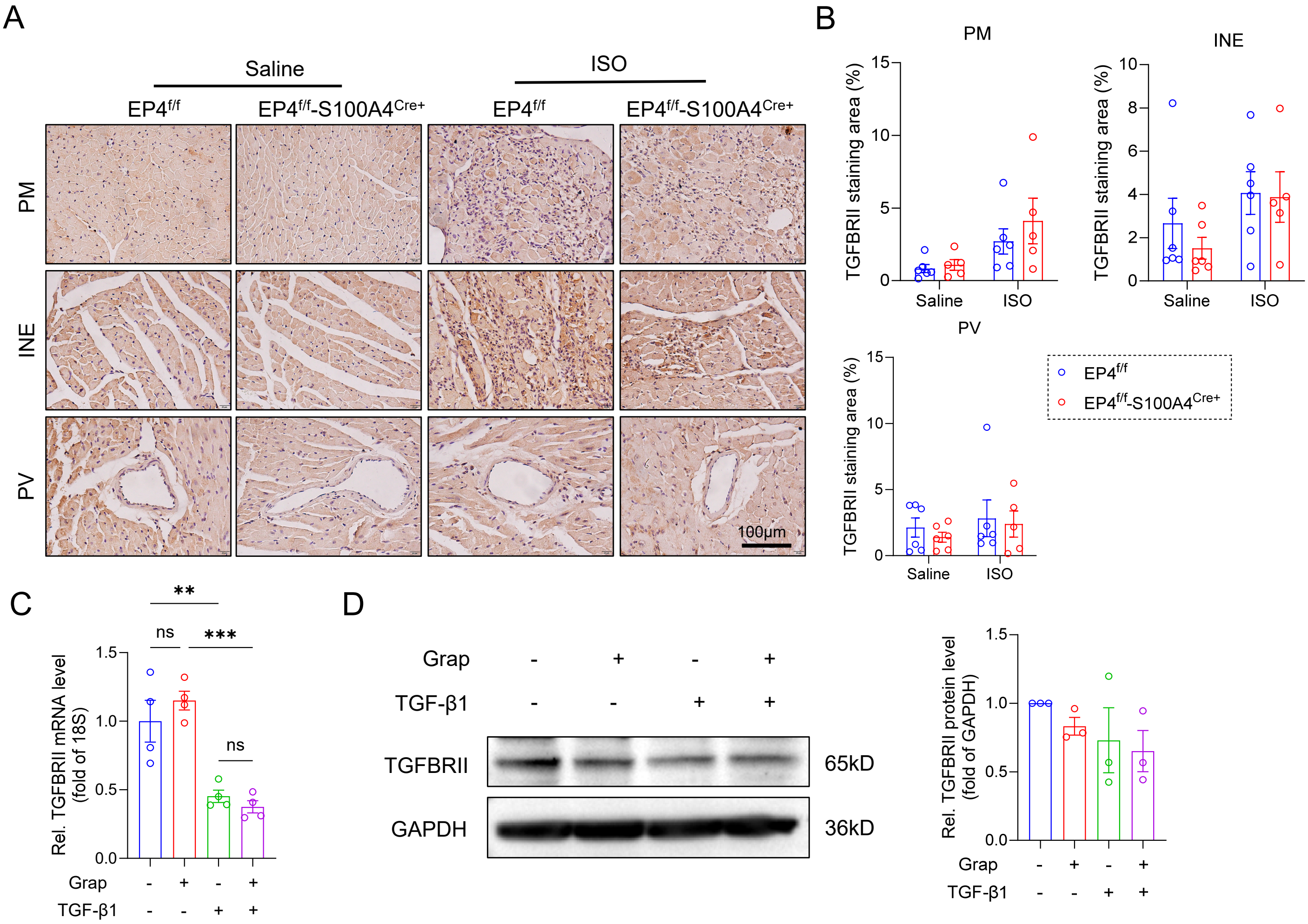

Figure S18. EP4 deletion or inhibition does not affect the expression of TGFBRII in CFs.
A&B. Immunohistochemical staining of TGFBRII in the hearts of the male EP4$^{f/f}$ and EP4$^{f/f}$-S100A4^{Cre+} mice after treatment with saline or ISO for 7 days. Papillary muscle (PM) area, interstitial area near the endocardium (INE), and perivascular (PV) area were shown, respectively (**A**). Quantitative analysis of TGFBRII-positive areas was performed by image J software (**B**). Scale bar=100µm. n=5-6 per group. **C.** qRT-PCR analysis was performed to measure the mRNA expression of TGFBRII in NRCFs pretreated with or without grapiprant (1µM) for 30 min, followed by treatment with TGF-β1 (5ng/mL) for 6 hours. n=4. **D**. Western blot assay was conducted to determine the protein expression of TGFBRII in NRCFs pretreated with grapiprant (1µM) for 30 min, followed by TGF-β1 (5ng/mL) treatment for 24 hours. The quantitative analysis of TGFBRII protein level was performed by image J software. n=3. Data were presented as mean±SEM. **P<0.01, ***P<0.001 by one-way ANOVA followed by the Tukey's multiple comparisons test.

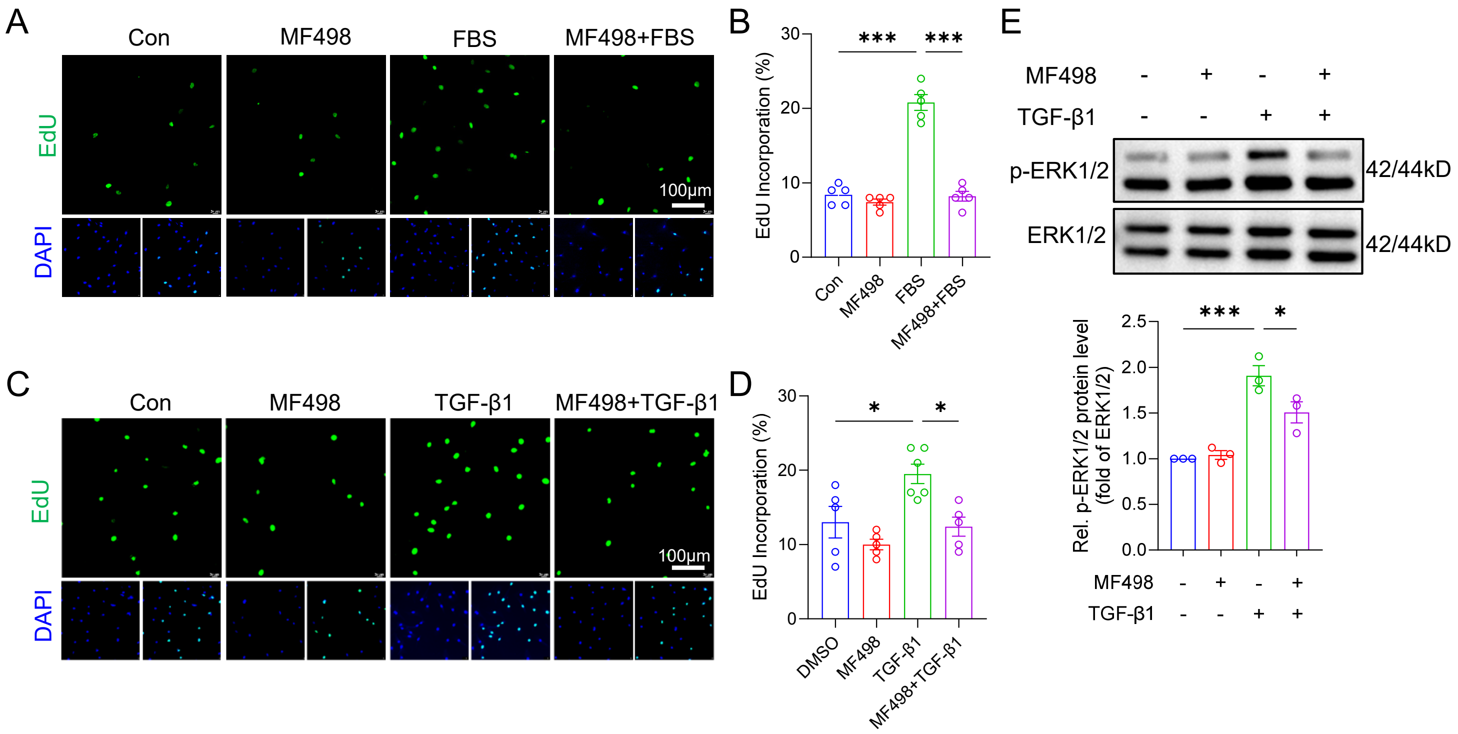

Figure S19. MF498 inhibits FBS- and PDGF-BB-induced CF proliferation.

A&B. EdU incorporation analysis showing the effect of MF498 treatment on FBS-induced CF proliferation. The primary cultured neonatal rat cardiac fibroblasts (NRCFs) were pretreated with or without MF498 (0.1μM) for 30 min, followed by FBS (10%) treatment for 24 hours. The percentage of EdU-positive cell (green) relative to DAPI (blue) was calculated (**B**). Scale bar=100μm. n=5. **C&D**. EdU incorporation analysis showing the effect of MF498 treatment on TGF-b1-induced CF proliferation. The NRCFs were pretreated with or without MF498 (0.1μM) for 30 min, followed by TGF-β1 (10ng/mL) treatment for 24 hours. The percentage of EdU-positive cell (green) relative to DAPI (blue) was calculated (I). Scale bar=100μm. n=5. **E**. Western blot analysis of the total and phosphorylated ERK1/2 and total ERK1/2 levels in the NRCFs. The cells were pretreated with or without MF498 (0.1μM) for 30 min and then with TGF-β1 (10ng/mL) for another 30min. Quantitative analysis was performed using image J software. n=3. Data were presented as mean±SEM. *P<0.05, ***P<0.001 by one-way ANOVA followed by the Tukey's multiple comparisons test.

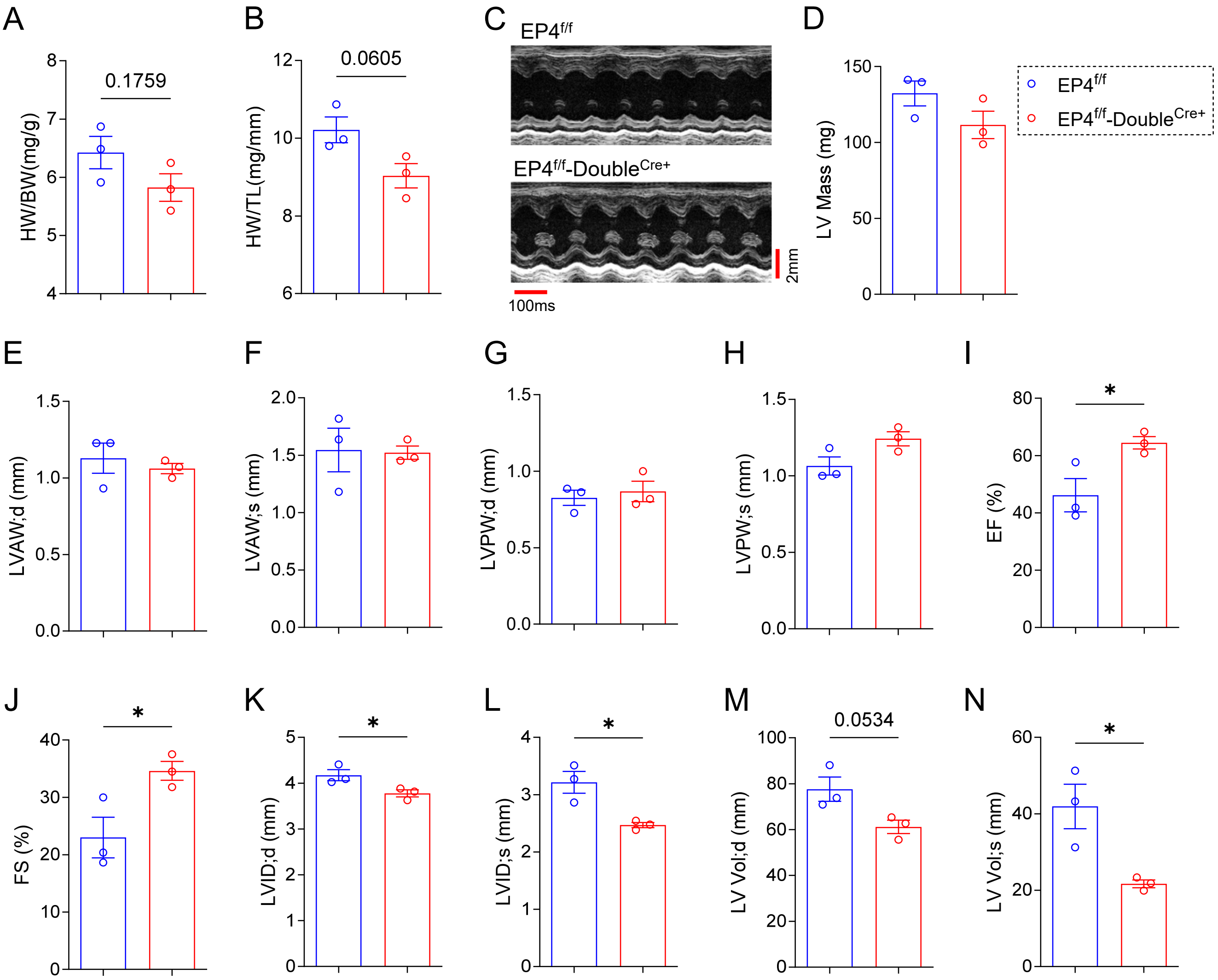

Figure S20. EP4 gene double knockout in the CFs and CMs slightly improves ISO-induced cardiac hypertrophy and systolic function.

A&B. The heart weight (HW, mg) to body weight (BW, g) ratio (HW/BW) (**A**) and the HW (mg) to tibia length (TL, mm) ratio (HW/TL) (**B**) in male EP4$^{f/f}$ and EP4$^{f/f}$-Double^{Cre+} mice receiving 7 days' saline or ISO injection. n=3 mice per group. **C**. Representative M-mode echocardiography in male EP4$^{f/f}$ and EP4$^{f/f}$-Double^{Cre+} mice injected with saline or ISO for 7 days. Transverse scale bar=100ms. Vertical scale bar=2mm. **D-N**. Echocardiography measurements of left ventricular mass (LV mass) (**D**), left ventricular end-diastolic anterior wall thickness (LVAW;d) (**E**), left ventricular end-systolic anterior wall thickness (LVAW;s) (**F**), left ventricular end-diastolic posterior wall thickness (LVPW;d) (**G**) and left ventricular end-systolic posterior wall thickness (LVPW;s) (**H**), ejection fraction (EF) (**I**), fractional shortening (FS) (**J**), left ventricular end-diastolic internal diameter (LVID;d) (**K**) and left ventricular end-systolic internal diameter (LVID;s) (**L**), left ventricular end-diastolic volume (LV Vol;d) (**M**) and left ventricular end-systolic internal diameter (LV Vol;s) (**N**) in male EP4$^{f/f}$ and EP4$^{f/f}$-Double^{Cre+} mice after 7 days saline or ISO injection. n=3 mice per group. Data were presented as mean±SEM. *P<0.05 by two-tailed unpaired t test.

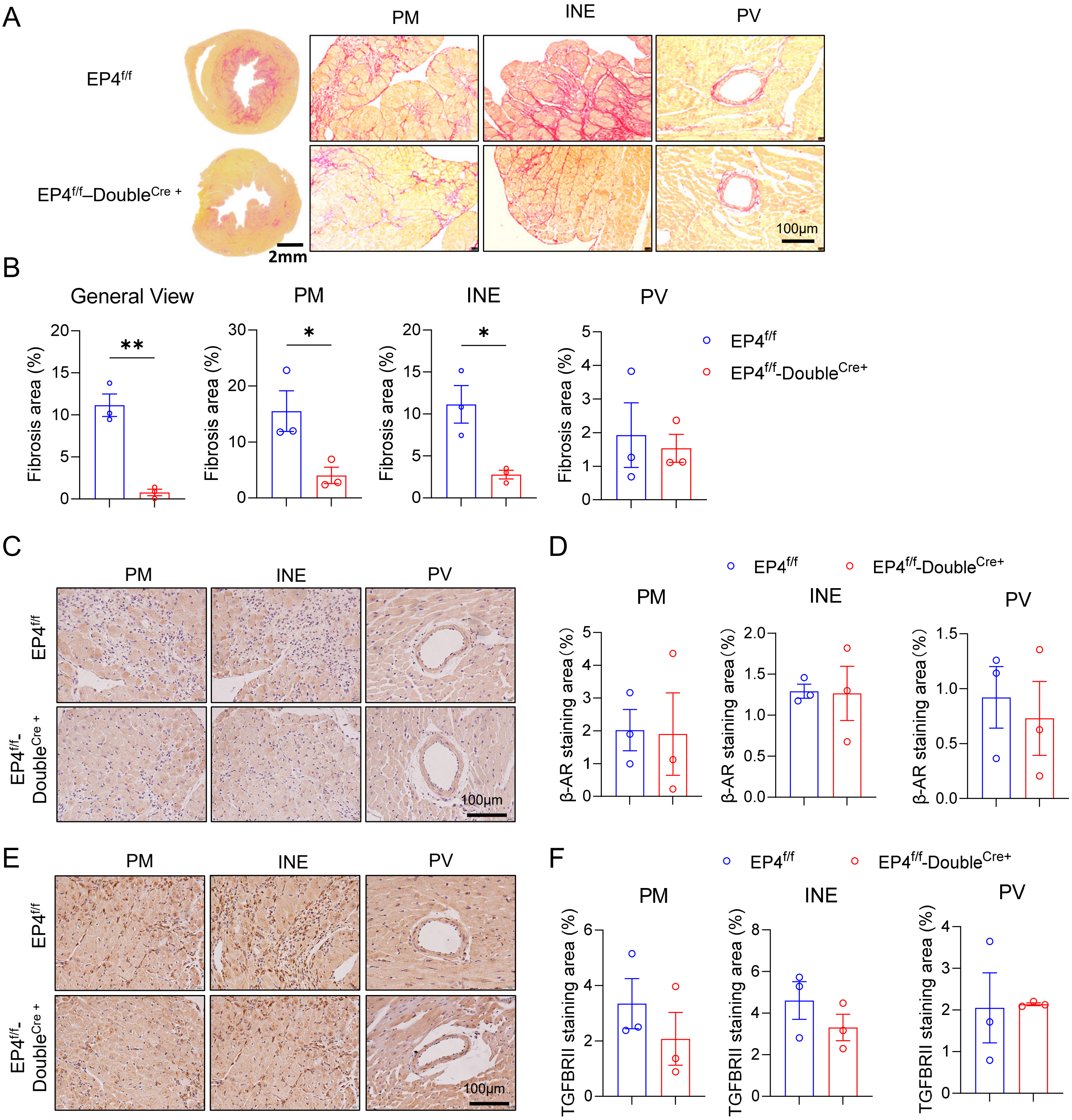

Figure S21. Simultaneous deletion of EP4 in the CFs and CMs improves ISO-induced ECM deposition. A&B. Representative images of Sirius red staining showing the hearts of the male EP4$^{f/f}$ and EP4$^{f/f}$-Double^{Cre+} mice receiving ISO injection for 7 days. General view, papillary muscle (PM) area, interstitial area near the endocardium (INE), and perivascular (PV) area were shown respectively (**A**). Quantitative analysis of fibrotic areas (red) was performed by image J software (**B**). General view scale bar=2mm. Enlarged picture scale bar=100μm. n=3 per group. **C-F**. Representative immunohistochemical staining of β-AR (**C**) and TGFBRII (**E**) in the hearts of the male EP4$^{f/f}$ and EP4$^{f/f}$-Double^{Cre+} mice receiving ISO injection for 7 days. Papillary muscle (PM) area, interstitial area near the endocardium (INE), and perivascular (PV) area were shown, respectively. Quantitative analysis of β-AR-positive areas (**D**) and TGFBRII-positive areas (**F**) was performed by image J software. Scale bar=100μm. n=3 per group. Data were presented as mean±SEM. *P<0.05, **P<0.01 by two-tailed unpaired t test.

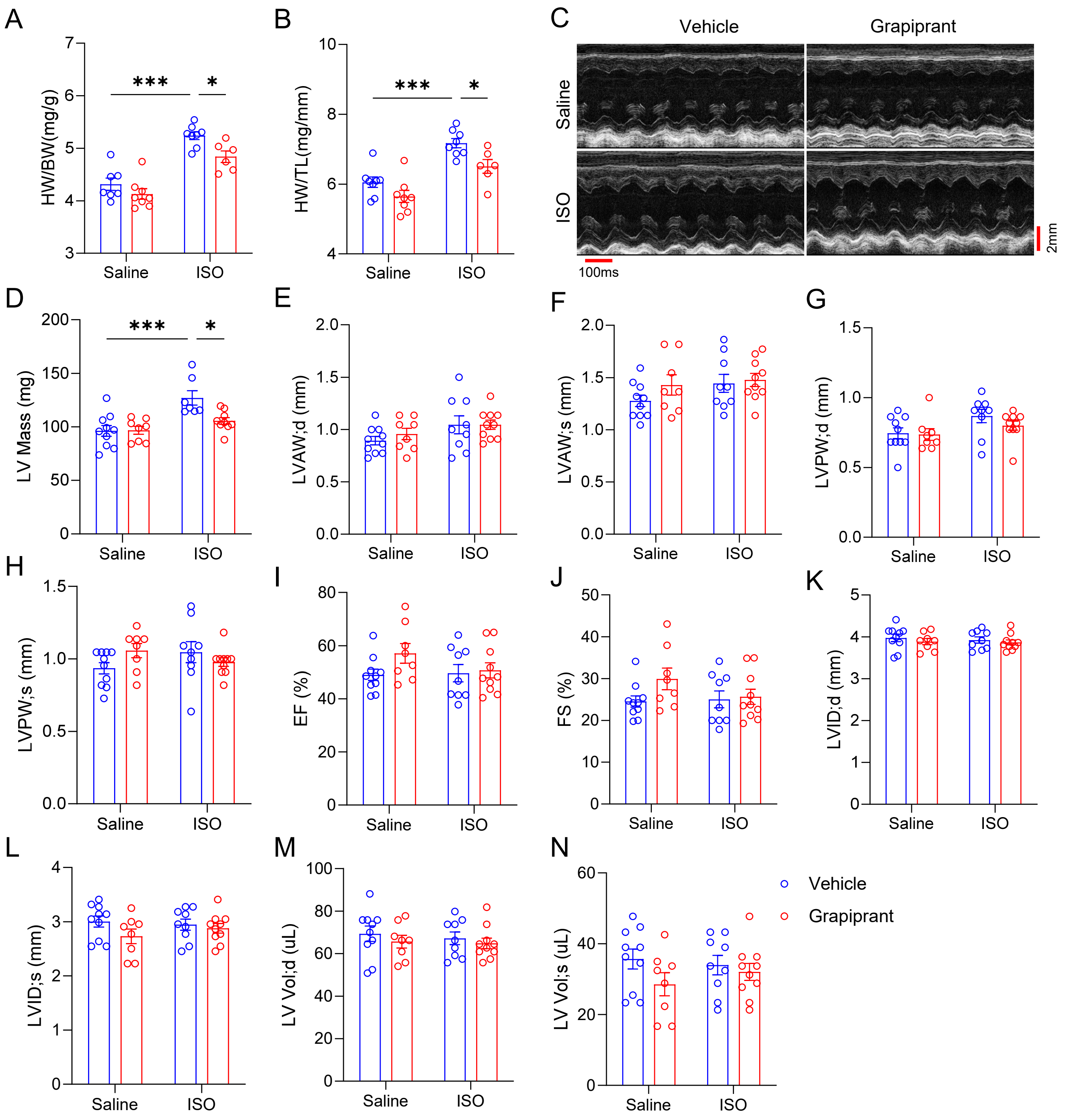

Figure S22. Grapiprant improves ISO-induced cardiac hypertrophy with little impact on cardiac systolic function.

A&B. The heart weight (HW, mg) to body weight (BW, g) ratio (HW/BW) (**A**) and the HW (mg) to tibia length (TL, mm) ratio (HW/TL) (**B**) in male mice, who were pretreated with grapiprant for 1 day, followed by ISO plus grapiprant for 7 days. n=6-8 mice per group. **C**. Representative M-mode echocardiography in mice shown in **A&B**. Transverse scale bar=100ms. Vertical scale bar=2mm. **D-N**. Echocardiography measurements of left ventricular mass (LV mass) (**D**), left ventricular end-diastolic anterior wall thickness (LVAW;d) (**E**), left ventricular end-systolic anterior wall thickness (LVAW;s) (**F**), left ventricular end-diastolic posterior wall thickness (LVPW;d) (**G**), left ventricular end-systolic posterior wall thickness (LVPW;s) (**H**), ejection fraction (EF) (**I**), fractional shortening (FS) (**J**), left ventricular end-diastolic internal diameter (LVID;d) (**K**), left ventricular end-systolic internal diameter (LVID;s) (**L**), left ventricular end-diastolic volume (LV Vol;d) (**M**) and left ventricular end-systolic internal diameter (LV Vol;s) (**N**) in mice shown in **A&B**. n=8-10 mice per group. Data were presented as mean±SEM. *P<0.05, ***P<0.001 by two-way ANOVA followed by the Tukey's multiple comparisons test.

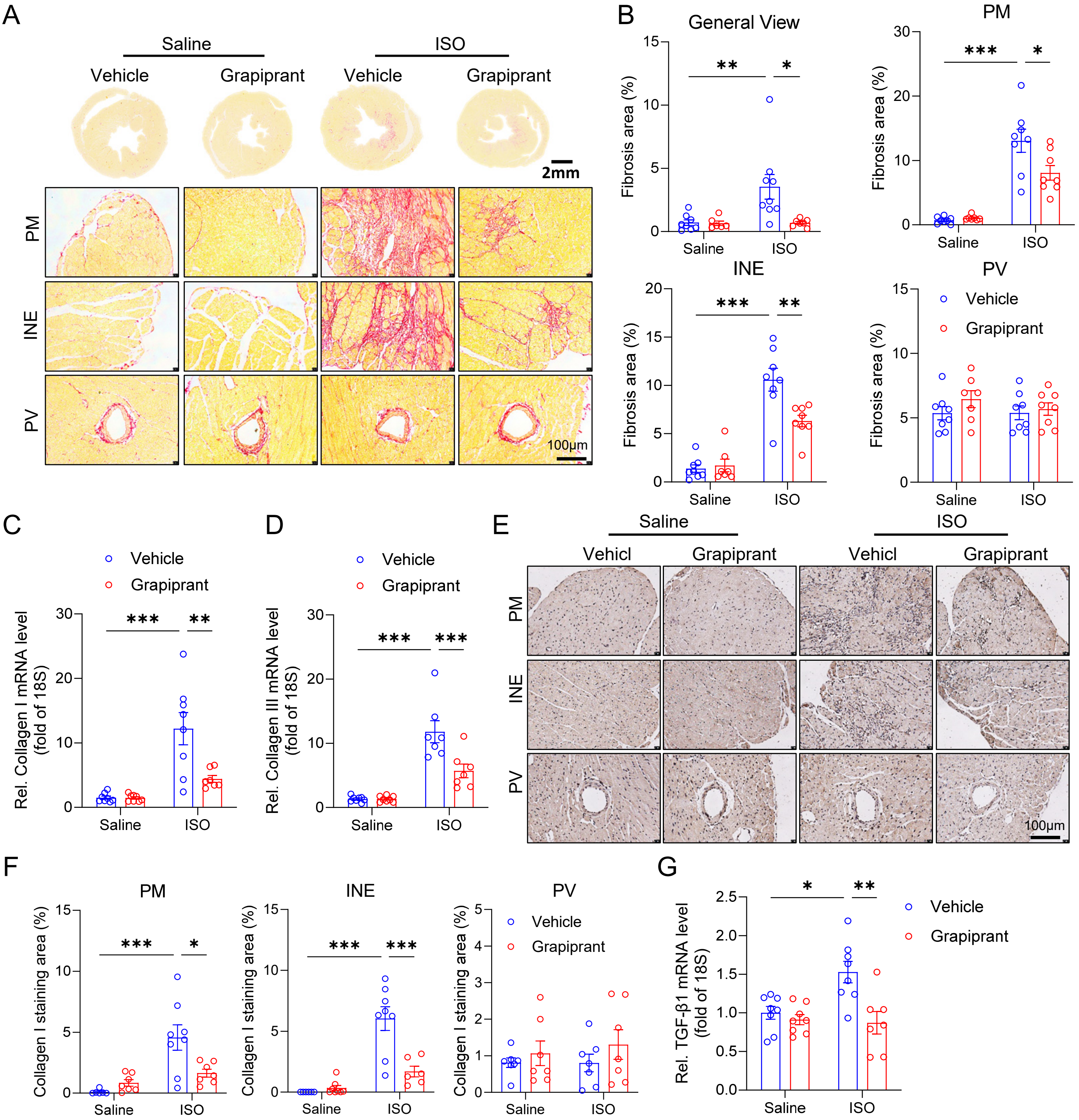

Figure S23. Grapiprant improves ISO-induced cardiac ECM deposition.

A&B. Representative images of Sirius red staining of heart tissues. The wild-type male mice were treated with grapiprant (3mg/kg/day, po) or vehicle from day 0 to day 7 and ISO (5mg/kg/day) from day 1 to day 7. At the end of the experiment, the mice were performed echocardiography before sacrificed. General view, papillary muscle (PM) area, interstitial area near the endocardium (INE), and perivascular (PV) area were shown, respectively. Quantitative analysis of fibrotic areas (red) was performed by image J software. General view scale bar=2mm. Enlarged picture scale bar=100μm. n=6-9 per group. **C&D**. qRT-PCR analysis of the mRNA levels of collagen I (**C**) and collagen III (**D**) in the hearts of mice treated with grapiprant and ISO. n=7-8 per group. **E&F**. Representative immunohistochemical staining of collagen I in the hearts of mice treated with grapiprant and ISO. Papillary muscle (PM) area, interstitial area near the endocardium (INE), and perivascular (PV) area were shown, respectively. Quantitative analysis of Collagen I-positive areas was performed by image J software. Scale bar=100μm. n=6-7 per group. **G**. qRT-PCR analysis of the mRNA levels of TGF-β1 in the hearts of mice receiving grapiprant treatment and ISO treatment. n=7-8 per group. Data were presented as mean±SEM. *P<0.05, **P<0.01, ***P<0.001by two-way ANOVA followed by the Tukey's multiple comparisons test.

Table S1. Primers used for real-time PCR.

Gene Name	Forward primer sequence (5′-3′)	Reverse primer sequence (5′-3′)
Mouse EP4	CCTTCACCACGTTTGGCTGAT	ATGGTCATCTTACTCATCGCCAC
Mouse18s	CCATCCAATCGGTAGTAGCG	GTAACCCGTTGAACCCCATT
Mouse Collagen I	TGGAAACCCGAGGTATGCTT	CTTGGGTCCCTCGACTCCTAC
Mouse Collagen III	TCAAGCCTGAAGGAAACAGCA	CGATGGGTAGTCTCATTGCC
Mouse TGF-β1	ACCGCAACAACGCCATCTAT	TTCCGTCTCCTTGGTTCAGC
Mouse COX-1	GAAGGAGTCTCTCGCTCTGGTT	TGGTAGTTGTCGAGGCCAAAG
Mouse COX-2	AGGTCATTGGTGGAGAGGTG	CCTGCTTGAGTATGTCGCAC
Rat EP4	ATTCTGATGGCCTGCAAATC	GGTGCAGAGATCCAGATGGT
Rat 18s	GTAACCCGTTGAACCCCATT	CCATCCAATCGGTAGTAGCG
Rat Collagen I	GTACATCAGCCCAAACCCCA	TCGCTTCCATACTCGAACTGG
Rat Collagen III	TGGGAAAGGTGAAATGGGTCC	CTTTGCTCCATTCTTGCCCG
Rat TGF-β1	GACCGCAACAACGCAATCT	CACTCAGGCGTATCAGTGGG
Rat Adrb1	CCCAAGTCGGTTAACAGCGA	TGTCGTTCTTCCTCCACACG
Rat TGBFRII	TCCCAAGTCGGTTAACAGCG	TGTCGTTCTTCCTCCACACG

The annealing temperature was 59°C for all genes examined.

Table S2. Primers used for genotyping.

Gene Name	Primer sequence (5′-3′)	Products length (bp)	Annealing temperature
EP4 genotyping (F1 and R2)	F: GGAGTCACTTTTCCCTTGAGAAG<br>R: AGCGAGTCCTTAGGCTTTTAAGT	370(KO)<br>1811(WT)	59℃
Flox genotyping (F2 and R2)	F: TCTGTGAAGCGAGTCCTTAGGCT<br>R: CGCACTCTCTCTCTCCCAGGAA	243(WT)<br>344(flox)	60℃
S100A4-Cre genotyping	F: TGCCACGACCAAGTGACAGCAATG<br>R: ACCAGAGACGGAAATCCATCGCTC	377	60℃
α-MyHC-Cre genotyping	F: ATGACAGACAGATCCCTCCTATCTCC<br>R: CTCATCACTCGTTGCATCATCGAC	300	60℃

Table S3. Binding energy and hydrogen bonding interaction of EP4-TGFBRII complex.

	Model Predictions	Binding Free Energy (MM/GBSA)	Hydrogen Bonding EP4(A)-TGFBRII(B)
1	Model 2	-39.64 kcal/mol	GLN253(A)-GLU94(B); ILE223(A)-SER24(B)
2	Model 3	-36.00 kcal/mol	SER247(A)-ASP44(B); SER1(A)-GLU45(B)
3	Model 6	-34.21 kcal/mol	GLN253(A)- SER24(B); LYS283(A)-ASP44(B)
4	Model 7	-34.02 kcal/mol	ARG273(A)-ASP62(B)
5	Model 9	-28.77 kcal/mol	GLN253(A)-ILE28(B); GLN253(A)-THR26(B); CYS224(A)-ASP7(B)
6	Model 10	-19.50 kcal/mol	ARG273(A)-GLU83(B)
